# LUNGBANK: a novel biorepository strategy tailored for comprehensive multiomics analysis and P-medicine applications in lung cancer

**DOI:** 10.55730/1300-0152.2696

**Published:** 2024-05-28

**Authors:** Dilek DEMİRCAN ÇEKER, Volkan BAYSUNGUR, Serdar EVMAN, İlker KOLBAŞ, Abdurrahim GÖRDEBİL, Sinem M. NALBANTOĞLU, Yusuf TAMBAĞ, Ömer KAÇAR, Ahmet MİDİ, Hatice ASLANOĞLU, Nülüfer KARA, Nilgün ALGAN, Ayberk BOYACIOĞLU, Betül KARADEMİR YILMAZ, Ali ŞAHİN, Hivda ÜLBEĞİ POLAT, Abidin ŞEHİTOĞULLARI, Ali Osman ÇIBIKDİKEN, Mücahit BÜYÜKYILMAZ, İbrahim Berkan AYDİLEK, Abdulkerim ENEŞ, Sevde KÜÇÜKER, Fatih KARAKAYA, İhsan BOYACI, Mahmut GÜMÜŞ, Onur ŞENOL, Merve ÖZTUĞ, Evren SABAN, Ömer SOYSAL, Nur BÜYÜKPINARBAŞILI, Akif TURNA, Mehmet Zeki GÜNLÜOĞLU, Aslı ÇAKIR, Şaban TEKİN, Uygar TAZEBAY, Abdullah KARADAĞ

**Affiliations:** 1 Molecular Oncology Laboratory, Medical Biotechnology Research Group, VPLS, TÜBİTAK Marmara Research Center, Kocaeli, Turkiye; 2 Department of Molecular Biology and Genetics, Gebze Technical University, Kocaeli, Turkiye; 3 Department of Thoracic Surgery, Faculty of Medicine, University of Health Sciences, İstanbul, Turkiye; 4 Department of Thoracic Surgery, Süreyyapaşa Training and Research Hospital, İstanbul, Turkiye; 5 Software Technologies Research Institute, TÜBİTAK Informatics and Information Security Research Center, Ankara, Turkiye; 6 Department of Pathology, Faculty of Medicine, Bahçeşehir University, İstanbul, Turkiye; 7 Division of Biochemistry, Department of Basic Medical Sciences, Faculty of Medicine, Marmara University, İstanbul, Turkiye; 8 Genetic and Metabolic Diseases Research and Investigation Center (GEMHAM), Marmara University, İstanbul, Turkiye; 9 Department of Thoracic Surgery, Faculty of Medicine, Sakarya University, Sakarya, Turkiye; 10 Department of Computer Sciences and Engineering, KTO Karatay University, Konya, Turkiye; 11 Ecolarium Digital Technologies Inc., İstanbul, Turkiye; 12 Department of Computer Engineering, Faculty of Engineering, Harran University, Şanlıurfa, Turkiye; 13 Department of Internal Medicine, Faculty of Medicine, İstanbul Medipol University, İstanbul, Turkiye; 14 Department of Internal Medicine, Faculty of Medicine, İstanbul Medeniyet University, İstanbul, Turkiye; 15 Department of Analytical Chemistry, Faculty of Pharmacy, Atatürk University, Erzurum, Turkiye; 16 TÜBİTAK National Metrology Institute, Kocaeli, Turkiye; 17 Department of Thoracic Surgery, Faculty of Medicine, Bezmialem Vakıf University, İstanbul, Turkiye; 18 Department of Pathology, Faculty of Medicine, Bezmialem Vakıf University, İstanbul, Turkiye; 19 Department of Thoracic Surgery, Faculty of Medicine, İstanbul University-Cerrahpaşa, İstanbul, Turkiye; 20 Department of Thoracic Surgery, Faculty of Medicine, İstanbul Medipol University, İstanbul, Turkiye; 21 Department of Pathology, Faculty of Medicine, İstanbul Medipol University, İstanbul, Turkiye; 22 Division of Medical Biology, Department of Basic Medical Sciences, Faculty of Medicine, University of Health Sciences, İstanbul, Turkiye; 23 Institute of Biotechnology, Gebze Technical University, Kocaeli, Turkiye

**Keywords:** Lung cancer, biorepository, biobank, P-medicine, integrative multiomics

## Abstract

**Background/aim:**

LUNGBANK was established as part of Project LUNGMARK, pioneering a biorepository dedicated exclusively to lung cancer research. It employs cutting-edge technologies to streamline the handling of biospecimens, ensuring the acquisition of high-quality samples. This infrastructure is fortified with robust data management capabilities, enabling seamless integration of diverse datasets. LUNGBANK functions not merely as a repository but as a sophisticated platform crucial for advancing lung cancer research, poised to facilitate significant discoveries.

**Materials and methods:**

LUNGBANK was meticulously designed to optimize every stage of biospecimen handling, from collection and storage to processing. Rigorous standard operating procedures and stringent quality control measures guarantee the integrity of collected biospecimens. Advanced data management protocols facilitate the efficient integration and analysis of various datasets, enhancing the depth and breadth of research possibilities in lung cancer.

**Results:**

LUNGBANK has amassed a comprehensive collection of biospecimens essential for unraveling the intricate molecular mechanisms of lung cancer. The integration of state-of-the-art technologies ensures the acquisition of top-tier data, fostering breakthroughs in translational and histological research. Moreover, the establishment of patient-derived systems by LUNGBANK underscores its pivotal role in personalized medicine approaches.

**Conclusion:**

The establishment of LUNGBANK marks a significant milestone in addressing the critical challenges of lung cancer research. By providing researchers with high-quality biospecimens and advanced research tools, LUNGBANK not only supports Project LUNGMARK’s objectives but also contributes extensively to the broader landscape of personalized medicine. It promises to enhance our understanding of lung cancer initiation, progression, and therapeutic interventions tailored to individual patient needs, thereby advancing the field towards more effective diagnostic and therapeutic strategies.

## 1. Introduction

Lung cancer is one of the most common and deadliest forms of cancer worldwide (Sung et al., 2021). It can be divided into two main types based on clinical presentation and histological features. Small cell lung cancer (SCLC) tends to grow and spread more quickly. Nonsmall cell lung cancer (NSCLC) is the most common type of lung cancer, accounting for about 85% of all cases. There are three main subtypes of NSCLC: adenocarcinoma, squamous cell carcinoma, and large cell carcinoma. The prognosis of lung cancer depends on the stage at which it is diagnosed. Although early-stage lung cancer has a better prognosis, approximately 70%–75% of patients are diagnosed at an advanced stage when tumor cells have spread, which prevents effective treatment and reduces overall survival rates (Lemjabbar-Alaoui et al., 2015; Schabath and Cote, 2019). There is an urgent need for a comprehensive lung cancer screening and early diagnosis system that includes in-depth molecular profiling at the DNA, RNA, protein, and metabolite levels (Toumazis et al., 2020). In addition, novel and potent therapeutics could be developed by identifying target molecules and pathways.

Biorepositories stand as essential resources in tackling worldwide health challenges attributed to aging populations and a rising prevalence of chronic diseases (Rutten-Jacobs et al., 2018). These repositories symbolize the intersection of cutting-edge technologies, carefully curated biological samples, and related clinical and research data (Harris et al., 2012). Biobanks play a key role in advancing scientific and medical research, offering a wealth of phenotypic information that includes diagnoses, risk factors, and diverse clinical parameters (Coppola et al., 2019). Leveraging advanced methods like DNA sequencing and genotyping, biobanks enable extensive genomic studies, providing large datasets and substantial sample collections. However, they face challenges in managing data and handling complexities associated with nonrandom genetic variant associations and high-dimensional data (Chattopadhyay and Lu, 2019; Jimmy Juang et al., 2020; Sakaue et al., 2020). Furthermore, these initiatives collectively advance the field of precision medicine, opening avenues for personalized healthcare solutions (Khoury et al., 2016; Tarkkala et al., 2019). However, to guarantee inclusive representation in genetic studies and alleviate biases, global collaboration becomes indispensable. Biobanks, instrumental in conducting expansive genomic studies with substantial datasets and sample sizes, encounter challenges that highlight the constraints of singular genomics approaches (Lazareva et al., 2022). Acknowledging these limitations emphasizes the increasing significance of embracing integrative multiomics strategies and adopting personalized medicine approaches in genomics studies (Lu et al., 2022). The evolving landscape underscores the need for a holistic and personalized grasp of genetic information to propel advancements in healthcare tailored to individual needs.

Biorepositories play a vital role in modern biomedical research by overseeing the acquisition, preservation, handling, and organization of biological specimens and clinical information derived from human participants (Day and Stacey, 2008; Kinkorová and Topolčan, 2020). They also leverage advanced technologies such as omics data, bioinformatics, artificial intelligence, and systems biology to enhance their value, ultimately contributing to the expedited understanding of diseases, the advancement of treatments, and the support of precision medicine initiatives, particularly as we approach the era of smart healthcare systems (Han et al., 2014; Carey et al., 2016; Kinkorová, 2016; Nalbantoglu and Karadag, 2019; Kinkorová and Topolčan, 2020). The integration of comprehensive biological profiles is crucial for revealing disease-related patterns, mechanisms, and biomarkers, supporting targeted screening, diagnostics, therapeutics, and early risk evaluation (Bayat 2002; Horgan and Kenny, 2011; Goossens et al., 2015; Coppola et al., 2019; Biswas and Chakrabarti, 2020; Bhardwaj et al., 2022; Poetsch and Li, 2023). Furthermore, biorepositories with longitudinal data facilitate disease monitoring, treatment assessment, and relapse prediction, while advancements in P-medicine enable the customization of therapies to an individual’s biological profile (Annaratone et al., 2021; Kim, 2021).

Project LUNGMARK was initiated to achieve our objective, encompassing the incorporation of an advanced bioinformatics-supported integrative multiomics approach, alongside the utilization of high-throughput technologies such as next-generation sequencing (NGS) and mass spectrometry coupled with liquid chromatography (LC-MS/MS). The involvement of patient samples was critical, ensuring accurate and meticulous collection, processing, storage, and data management. We designed a lung-cancer-specific biorepository system, comprising two essential elements: an extensive sample collection and a comprehensive data management infrastructure to streamline and enhance these efforts. The primary goals of the project encompassed three key aspects; recording comprehensive patient information, maintaining detailed records of collected samples, and documenting both raw and processed data from integrative multiomics studies. Integrating existing knowledge enhanced the dataset, offering a comprehensive perspective of the disease. The project’s goal was to enhance our understanding of the disease and foster improvements in diagnostic, screening, monitoring, and therapeutic methods by establishing connections between the molecular profile and both clinical, histological, and imaging data.

Our advanced biorepository efficiently collected samples from both lung cancer patients and normal donors, carefully organizing units for collection, processing, storage, and management. A dedicated clinical team skillfully acquired various specimens, including normal lung tissue, primary lung tumors, reactive stroma, lymph nodes, blood/serum/plasma, urine, saliva, sputum, and stool. Solid samples were promptly either snap-frozen or preserved in suitable solutions, depending on their intended application, and then subjected to thorough processing in accordance with precise protocols. Clinical information was securely stored within a meticulously barcoded database called LUNGBASE, expertly managed by LUNGSOFT. Rigorous assessment of DNA/RNA quality and quantity ensured the provision of high-quality samples for NGS and LC-MS/MS analysis. We also prepared frozen sections and rigorously verified sample identities through hematoxylin and eosin (H&E) staining, alongside the hospital’s pathological assessment of the patients. Moreover, our approach has incorporated patient-derived cell (PDC), patient-derived organoid (PDO), and patient-derived xenograft (PDX) models, and advanced exosome isolation techniques, enhancing the capabilities of personalized lung cancer research.

## 2. Materials and methods

### 2.1. Lung-cancer-specific biorepository, ethical considerations, institutional review board, and recruitment

**Figure 1:**
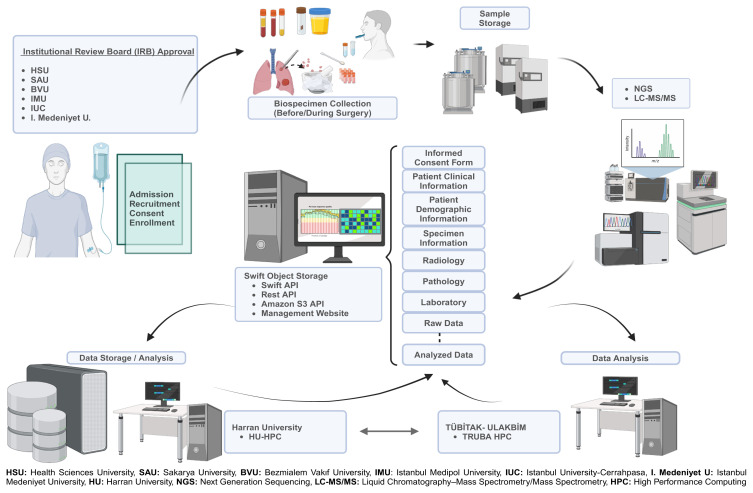
The figure visually represents the flow of samples throughout the entire biorepository workflow, spanning from the initial recruitment, enrollment, collection, and processing stage to subsequent storage and utilization. Compliance with ethical standards was upheld through IRB approval and the administration of consent forms. Collected biological specimens were transferred, processed, and stored in specialized facilities, including freezers and liquid nitrogen tanks under sterile and RNAse-free conditions. A dedicated database, together with associated software tailored for the biorepository, streamlined data management and ensured data security with geographically dispersed backups. Only authorized researchers were granted access, while stringent QC measures and regulatory adherence were consistently maintained.

The lung-cancer-specific biorepository system integrates various elements, including ethical considerations, patient recruitment protocols, a 96-terabyte data repository, specialized database management software, and sample acquisition, characterization, and quality control (QC) assessments (Figure 1). We gained valuable insights for establishing the biobank by referencing the BBMRI-ERIC directory on processes employed by European biobanks (Mayrhofer et al., 2016). We adhered to various guidelines, emphasizing clear objectives, adherence to standards, and protection of privacy (Network, 2007; Troyer, 2008; Vaught and Henderson, 2011; Harati et al., 2019). Privacy was addressed through ethical guidelines like the Declaration of Helsinki and Declaration of Taipei (World Medical Association, 2013; Dhai, 2016; Ballantyne, 2019). Our ethical recruitment, approved by the İstanbul Medipol University Ethics Committee (approval number: [10840098-01]), involved informed consent, privacy emphasis, and data deidentification. Institutional review board (IRB) approval and informed consent upheld ethical standards, ensuring privacy (Network, 2007; Troyer, 2008; Vaught and Henderson, 2011; Harati et al., 2019). Data were sourced from patient records by clinical team ensuring accuracy and minimizing bias, while our analysis considered factors like age, sex, histological subtype, and disease stage/grade for representative and clinically relevant findings. Patients received informed consent forms detailing study objectives, risks, benefits, and rights. We adhered to a rigorous recruitment process, enrolling stage I–IIIA NSCLC patients across all subtypes, excluding those with prior treatments, incomplete medical records, or significant comorbidities.

### 2.2. Sample collection, processing, and storage procedures

**Figure 2:**
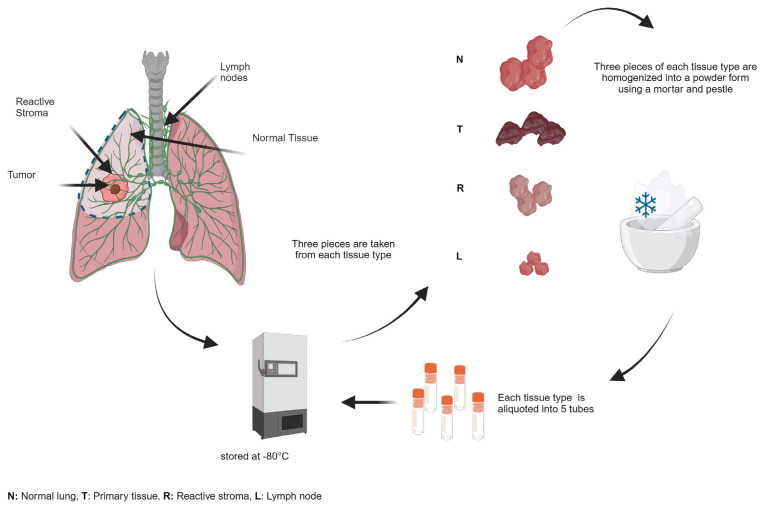
Figure illustrates the homogenization procedure for solid tissue specimens.

**Figure 3:**
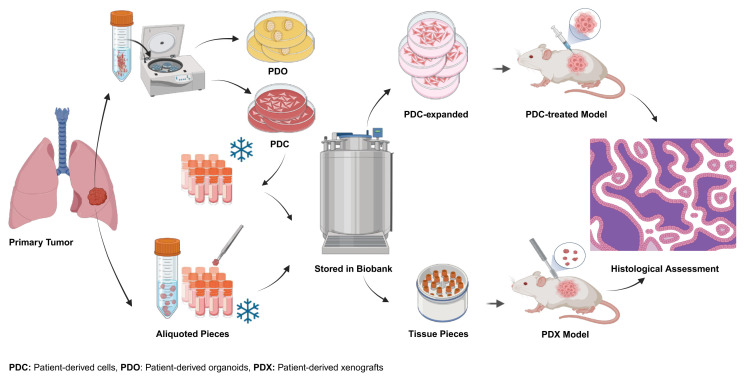
Figure illustrates the multifaceted procedures involved in lung cancer. Tumor cell isolation for patient-derived cell (PDC) and patient-derived organoid (PDO) models, as well as the storage of tumor tissue for patient-derived xenograft (PDX) models and exosome isolation. The process commences with the collection of tumor specimens during surgery, followed by cell isolation to establish PDC and PDO models. A fraction of the surgically obtained tumor tissue is carefully preserved for PDX models in specialized storage units. Additionally, exosomes are isolated from these surgically acquired tumor samples.

Our commitment to preserving the integrity of the biological specimens collected from lung cancer patients was firm and detailed. We maintained a close collaboration with healthcare professionals, ensuring the highest standards of quality and reliability in specimen collection, processing, and storage. Snap-frozen solid tissue samples underwent thorough homogenization following strict sterile and RNAse-free protocols, aiming to minimize potential bias in subsequent omics studies (Figure 2). The same rigorous standards were applied to blood samples, using a two-step centrifugation process to isolate plasma and serum while preventing hemolysis. We extended this careful approach to urine, saliva, sputum, bronchoalveolar lavage, and stool samples. We carefully preserved samples designated for PDC, PDO, and PDX models by immersing them in a freezing solution (90% FBS + 10% DMSO) and then storing in liquid nitrogen tanks (Figure 3). Exosomes were isolated with precision, resuspended in PBS, and stored at −20 °C, with continuous temperature monitoring and backup power systems ensuring their preservation. Our storage strategy involved systematic organization, barcoding, and creating multiple aliquots, all designed to minimize the potential impact of freeze-thaw cycles on the specimens.

### 2.3. QC measurements

Maintaining top-notch QC in our biorepository was crucial, especially for ensuring the suitability of specimens in various omics studies, including whole genome sequencing (WGS), transcriptomics analyses via NGS, and proteomics and metabolomics investigations employing LC-MS/MS. We rigorously implemented a set of stringent QC measures to guarantee the utmost reliability and accuracy of the data derived from our biorepository samples.

#### 2.3.1. Nucleic acids QC measures

QC procedures for nucleic acids were fundamental in safeguarding the integrity and precision of genetic material in our specimens. Three primary instruments for nucleic acid QC were the Thermo Fisher Scientific’s Qubit fluorometer and NanoDrop spectrophotometer, and Agilent’s Bioanalyzer. The Qubit fluorometer played a vital role in quantifying nucleic acids (DNA and RNA), utilizing specialized Qubit assay kits with fluorescent dyes. NanoDrop spectrophotometry, using UV absorbance, was used to assess DNA and RNA concentrations and to evaluate purity through ratios. The Bioanalyzer, a microfluidics-based platform, was used to comprehensively assess the nucleic acid quality and integrity. It utilized electrophoresis to examine size distribution and generate electropherograms and gel-like images. This advanced tool excelled in assessing RNA integrity and identifying size variations. Together, these instruments ensured reliable genetic material for the project, enhancing data accuracy while minimizing biases.

#### 2.3.2. Proteomics and metabolomics QC measures

We understood the crucial role of matrix blanks in our workflow, using them to create inclusion/exclusion lists that eliminated background noise and improved annotation precision. Additionally, blank samples were instrumental in identifying potential carry-over during chromatographic separation. We incorporated internal standards to maintain continuous oversight of instrument performance and promptly identify any discrepancies in sample preparation and analysis, enabling us to verify the quality of feature integration through software tools like XCMS or MZmine. Calibration standards with well-defined concentrations played a crucial role in achieving quantitative accuracy. We consistently analyzed QC samples in order to gauge the LC-MS/MS system’s reproducibility. This approach standardized instrument sensitivity across the entire analysis and helped filter out unexpected features.

Enhancing our quantitative proteomics workflow, we incorporated TMT reagents (Thermo Scientific, USA) for improved accuracy and efficiency. This multiplexing method enables simultaneous analysis of four tissue samples from a single patient, enhancing our quantitative proteomics. We also established a systematic QC process for evaluating proteomics data, involving three key stages: 1) examination of raw data, 2) identification, and 3) quantification quality evaluation. These stages encompass various metrics to capture variations arising from sample handling, nanoLC-MS performance, and data processing.

### 2.4. Histological verification

Supplementing the hospital’s pathology report, we introduced a detailed histological validation procedure to confirm each solid tissue type and provide a visual depiction of their structural integrity and composition. This involved creating histological frozen sections from solid tissue specimens, which then underwent H&E staining. Furthermore, 35 immunohistochemistry and 15 in situ hybridization sections from each tissue were also prepared for the future studies.

In summary, our sample collection, processing, and storage procedures as well as QC measurements adhered to stringent protocols designed to ensure the integrity and quality of biological specimens. These meticulous measures were instrumental in generating reliable data for our lung cancer research, contributing to the credibility and robustness of our findings. All the standard operation procedures (SOPs) are provided through Supplementary Data-1.

## 3. Results

The biorepository collected a wide range of biological resources from multiple hospitals, which encompassed institutions such as İstanbul Süreyyapaşa Chest Diseases and Chest Surgery Training and Research Hospital, the Department of Thoracic Surgery at İstanbul Bezmialem Vakıf University Medical School, the Department of Thoracic Surgery at İstanbul University-Cerrahpaşa Medical School, the Department of Thoracic Surgery and Department of Internal Medicine at İstanbul Medipol University Medical School, the Department of Thoracic Surgery at Sakarya University Medical School, and the Department of Internal Medicine at İstanbul Medeniyet University Medical School.

### 3.1. Quantitative data analysis: unveiling the numeric patterns

**Figure 4:**
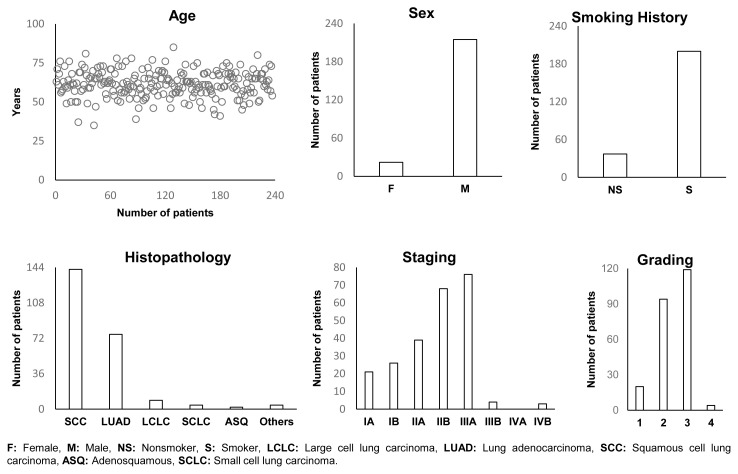
Quantitative data shows sex distribution, smoking history, age distribution, staging, grading, and distribution of lung cancer types.

LUNGBANK was established exclusively for the LUNGMARK project, which aimed to investigate five distinct tissue types sourced from 50 patients diagnosed with NSCLC. A total of 237 lung cancer patients who underwent surgery granted their consent to participate in the project. To uphold rigorous QC standards, patient enrollment for specimen collection was intentionally increased beyond the required number. This measure was implemented because the study encompasses no fewer than 20 distinct sample and analysis types. If any of these types failed to meet the QC standards, the corresponding patient was excluded from the study. Descriptive statistics, key epidemiological data, histological classifications, and tumor stage/grade categorizations are presented in Figure 4 and Supplementary Data-2.

### 3.2. Data collection, integration, and management

Precision in collecting, integrating, and managing data played a pivotal role in the lung cancer project to ensure the commencement of an integrated multiomics study with uniform starting materials for each individual omics investigation. We established detailed protocols to access patient health records, clinical assessments, and rich data sources, covering patient demographics, medical histories, and outcomes. We integrated diverse omics data types into a unified database, bridging molecular and clinical aspects.

#### 3.2.1. Database and software

LUNGBASE, an integral part of our research infrastructure, provided a sturdy foundation with an impressive 96 terabytes of storage capacity, as previously detailed (Cibikdiken et al., 2018). Archiving a comprehensive range of information, LUNGBASE encompassed patient profiles, clinic records, laboratory reports, pathology findings, and detailed staging and grading information. It also securely stored crucial PET-CT imaging data, providing invaluable insights into the disease. Furthermore, LUNGBASE was instrumental in storing comprehensive sample information, ensuring effortless tracking and accessibility for the research team. We seamlessly integrated the user-friendly LUNGSOFT data management platform to boost data management efficiency. This platform supported severe data security measures, ensuring the confidentiality of patient information. Our data management system also incorporated an advanced barcoding system, streamlining organization and retrieval. Each data entry was uniquely tagged with a barcode, enhancing data management. Additionally, a robust data backup system securely stored a duplicate database on a geographically remote server, ensuring data redundancy and preserving our invaluable dataset’s integrity and availability.

#### 3.2.2. Data management

A robust data management infrastructure was crucial to safeguard data integrity and security. Our comprehensive dataset resided on the dedicated secure LUNGBASE server equipped with robust access controls and encryption measures to prevent unauthorized access. We regularly conducted automated data backups as a precautionary measure against data loss. A rigorous QC framework was implemented at every stage of data collection and integration in order to maintain data quality.

#### 3.2.3. Radiomics and imaging data

Positron emission tomography–computed tomography (PET-CT) images were collected alongside biological samples, enriching our biobank’s comprehensiveness. The integration of imaging data with biospecimens allowed us to explore the intricate relationship between imaging characteristics and biological attributes, potentially enhancing both diagnosis and treatment strategies for lung cancer.

In summary, our approach to data collection, integration, and management demonstrates a commitment to rigorous standards, transparency, and ethical conduct. By harmonizing diverse data sources and implementing robust data management practices, we unlocked valuable insights into lung cancer, contributing to advancements in the development of screening, diagnosis, monitoring, and therapeutic systems.

### 3.3. Medical admission, recruitment, and enrollment

We obtained approval from the IRB and participant consent before securing funding for the LUNGMARK project. Our patient management workflow began with recruitment and the collection of medical histories and clinical information, emphasizing informed consent. Collaboration among healthcare experts ensured accurate biospecimens collection and labeling. Detailed records were stored in LUNGBASE. Biospecimens were handled with care, including real-time aliquoting under cold chain conditions, followed by preservation in liquid nitrogen or freezers at −20 °C/−80 °C. Comprehensive quality assessments involved qualitative and quantitative molecular characterizations, employing advanced techniques like gel electrophoresis, NanoDrop, Qubit, and ultrahigh throughput technologies. Our integrated workflow upheld ethical standards, ensuring data and biospecimen quality.

### 3.4. Collection, processing, and storage of specimens

SOPs were crafted to ensure the systematic and precise collection of diverse specimen types, as extensively detailed in Supplementary Data-1. All procedures were executed under stringent sterile conditions using sterile and RNAse-free equipment. During surgical procedures, specialized punchers were employed to obtain a minimum of 5 solid tissue samples, each approximately the size of a chickpea. These tissue specimens were subsequently placed into separate cryotubes. Tissue samples earmarked for omics studies were rapidly frozen in liquid nitrogen to maintain their structural integrity and subsequently stored at −80 °C in ultradeep freezers upon being transported to the laboratory on dry ice. Samples intended for histological verification were also collected and preserved in cryotubes on dry ice. Samples were immersed in a suitable tissue culture medium and transported through a controlled cold chain to maintain viability for creating PDC, PDO, and PDX models, as well as for exosome isolation. The tumor tissue was initially cleansed with penicillin/streptomycin in DPBS and then sliced into small fragments using a sterile scalpel. These minced tumor pieces were subsequently preserved in a freezing solution consisting of 90% fetal bovine serum (FBS) and 10% dimethyl sulfoxide (DMSO) if not needed immediately. When needed, the stored tumor tissue was thawed and centrifuged to remove the DMSO. The resulting pellet was then resuspended using the Miltenyi Tumor Dissociation Kit, human (130-095-929), following the specific cell isolation protocol outlined in the SOP. A portion of the solid tissue samples were dissected into 1.5–3-mm-thick pieces, then divided into aliquots in freezing solution, and finally preserved in liquid nitrogen. These preparations were earmarked for future experiments, including the establishment of PDX models and the isolation of exosomes. Liquid samples and stool specimens were collected according to established protocols, while bronchoalveolar lavage fluid was obtained during surgical procedures. Regardless of the sample type, each underwent careful processing and was divided into multiple containers to ensure redundancy and maintain sample quality. Solid tissue samples underwent an additional step, being homogenized into fine powder using sterile and RNAse-free equipment. These powdered tissue samples were then distributed into a minimum of 5 tubes, each designated for specific omics analysis, and stored at −80 °C until use.

**Figure 5:**
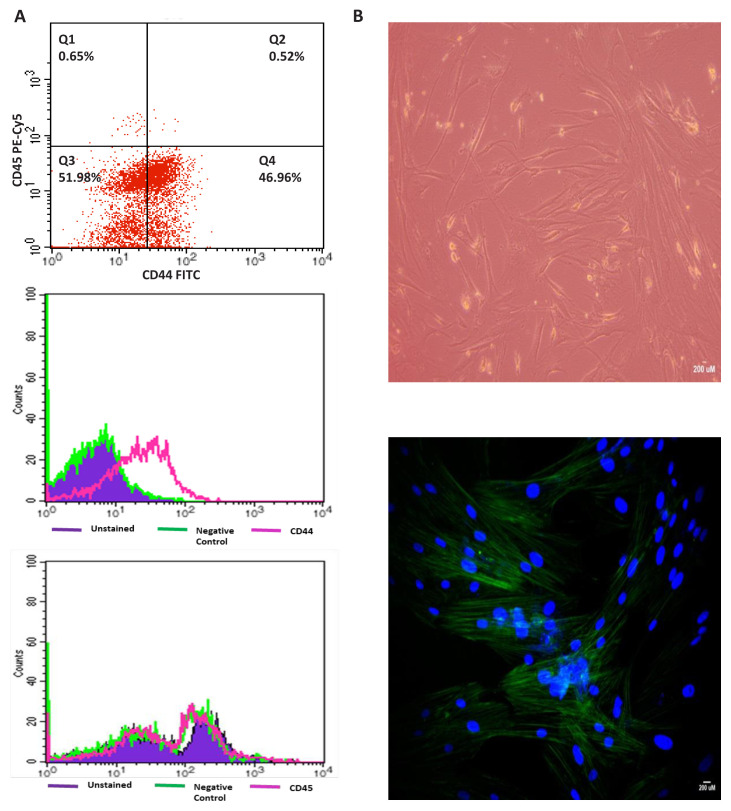
Characterization of PDC model. Lung cancer cells were isolated from a patient with squamous cell lung cancer, which expresses surface marker of CD44, but not CD45. A: CD44 positive and CD45 negative results by flow cytometry. B: Top panel shows image of cells through invert microscopy and down panel image of cells stained with F actin cytoskeletal dye and DAPI through fluorescent microscope for cell morphology (bar: 200 μm).

The biorepository incorporates exosomes that harbor valuable data comprising DNA, RNA, proteins, as well as metabolites and lipids (Kalluri and LeBleu, 2020). Exosomes play a central role in cell-to-cell communication and have been implicated in cancer progression, including lung cancer (Yin et al., 2021; Khan et al., 2022). Moreover, our biorepository offers PDC, PDO, and PDX models (Figure 5). These innovative models enable researchers to culture cells and tissues directly derived from patient samples. PDCs faithfully preserve the genetic and molecular characteristics of the original tumor, rendering them invaluable for drug testing and personalized treatment strategies (Yu et al., 2023). PDOs, on the other hand, accurately recapitulate the three-dimensional architecture of tumors and serve as a versatile platform for high-throughput drug screening (Botti et al., 2021). These models bridge the gap between traditional cell lines and in vivo animal models, offering a more faithful representation of human lung cancer biology (Kim et al., 2019a). Samples from our biorepository also serve as a valuable resource for the development of PDX models. PDCs or human tumor tissue is engrafted into immunodeficient mice, effectively replicating the patient’s tumor within an in vivo setting, as detailed by Kita et al. ( 2019). These PDX models offer a powerful platform for testing novel therapeutics and exploring treatment responses, closely mimicking the patient’s tumor environment (Kita et al., 2019). A total of 50 frozen sections (both for immunohistochemistry (IHC) and in situ hybridization (ISH) were prepared from all solid tissue samples, including normal lung tissue, primary tumor tissue, tumor microenvironment, and lymph node tissue for the confirmation of the tumor and tissue ID provided by pathology reports from the hospital. The resulting images and findings have been documented in Supplementary Data-3 following H&E staining.

### 3.5. QC and characterization

**Figure 6:**
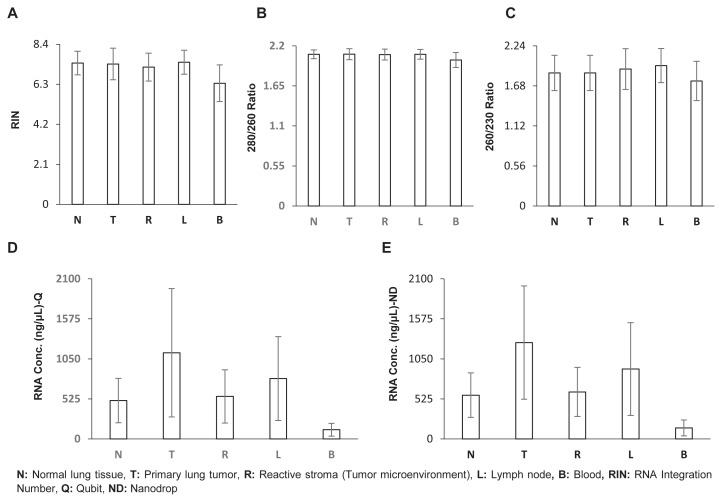
Illustration of QC measurements. A. RNA integration number, B. 280/260 ratio, C. 260/230 ratio, D. concentration by Qubit fluorometer, E. concentration by NanoDrop spectrophotometer.

The DNA and RNA extracted from the powdered aliquots, designated for WGS and RNA-Seq studies, underwent a rigorous and comprehensive characterization procedure. This involved precise quantification using Qubit fluorometry, assessment of purity via NanoDrop spectrophotometry, and evaluation of integrity through Bioanalyzer. These rigorous QC steps ensured that our genetic materials were free from contaminants, accurately quantified, and structurally intact. Descriptive statistics and values for all individual samples are shown in Figure 6 and supplementary Data-4. Furthermore, appropriate QC measures were applied before proteomics and metabolomics analysis through LC-MS/MS (data not shown). We assessed raw data quality using various metrics in the initial stage and found that total ion chromatograms exhibited a coefficient of variation of 5%–20%, while in the second stage, the standard filter-aided sample preparation (FASP) method outperformed in-solution digestion methods in terms of peptide and protein group identifications, highlighting the importance of data quality and effective normalization strategies in proteomics workflows.

## 4. Discussion

The establishment of a specialized biorepository dedicated to lung cancer marks a significant milestone in our efforts to unravel the intricate biological complexities of this disease. This repository offers an extraordinary array of sample types, expanding beyond conventional boundaries to encompass not only standard specimens like normal lung tissue and primary tumor tissue but also critical components such as adjacent tumor microenvironment tissue and lymph nodes, which are essential factors in comprehending cancer metastasis (Yu et al., 2015). Additionally, this repository plays a dual role, functioning not just as a storage facility for biological specimens but also as a comprehensive data repository, enriching physical samples with invaluable insights (Bondy, 2014; Rao et al., 2017). It systematically compiles pathology data, including highly detailed histological examinations, immunohistochemical assessments, and in situ hybridization analyses (Paskal et al., 2018). Moreover, the incorporation of radiologic imaging data provides a noninvasive perspective into the structural and functional aspects of lung tumors, enhancing our understanding of disease progression (Pinker et al., 2018; Sollini et al., 2020). Lastly, laboratory data delves deep into the molecular details of the samples, exploring gene expression profiles, proteomic landscapes, and metabolomic signatures (Liu and Pollard, 2015; Sears and Mazzone, 2020). Collectively, this comprehensive biorepository equipped us with an unparalleled resource to decode the multifaceted nature of lung cancer.

Our dedication to data management within the biorepository goes beyond clinical boundaries, encompassing an exhaustive catalog of sample data that not only intricately documents sample types and quantities but also provides detailed insights into the specific characteristics within the lung. This precision empowers accurate and targeted analyses. We curate both raw and analyzed multiomics data, including epi/genomic, epi/transcriptomic, epi/proteomic, and metabololipidomics data to capture the full spectrum of molecular complexity. Each layer of data provides a unique perspective into the complex biology of lung cancer, enriching our understanding of this intricate disease. This diverse range of sample types and accompanying data provided us with the tools to thoroughly investigate the molecular and genetic diversity present in lung cancer (Cai et al., 2021).

Biobanks, both general and disease-specific, stand as crucial pillars in scientific research, collecting and preserving diverse biological samples to support a spectrum of endeavors (Malsagova et al., 2020). General biobanks, with their broad approach, aim to cater to various research needs but often lack the specific focus required for in-depth understanding of particular pathologies, as they may not provide detailed molecular and clinical data (Washetine et al., 2018). In contrast, disease-specific biobanks, whether devoted to breast cancer or pancreatic cancer, navigate a nuanced landscape, revealing both shared strategies and unique approaches in sample collection and data integration (Balarajah et al., 2016; Bruna et al., 2016). These specialized repositories focus on specific diseases, offering a targeted research approach (Riegman et al., 2008). Transitioning to a broader context, biobanks emerge as transformative entities in health research, particularly within the realm of personalized medicine. Their significance is underscored by their role in targeted prevention, advanced diagnostics, and cost-effective treatment development, achieved by amalgamating biomarkers and clinical data to enrich health profiles. Within the field of personalized medicine in oncology, biobanks play a vital role in guiding optimal treatment strategies for specific cancer subtypes, utilizing comprehensive datasets that combine genetic and clinical information (Gambardella et al., 2020). As these biorepositories continue to evolve, their impact on advancing medical research and improving patient outcomes remains substantial, emphasizing the need for ongoing exploration and strategic utilization of their wealth of data. The collaborative efforts of general and disease-specific biobanks, through partnerships and shared initiatives, can further enhance their collective potential, fostering a comprehensive and impactful research environment. However, recognizing and addressing inherent challenges, such as selection bias, ethical considerations, and the need for sustained support and infrastructure, is vital to ensure the continued success and reliability of these invaluable resources.

LUNGBANK, distinguished by its exclusive dedication to lung cancer, exhibits a compelling advantage in its focused pursuit of unravelling the intricate biological nuances of this specific disease. This superiority is evident in its disease-specific orientation, employing a comprehensive multiomics approach that spans genomics, transcriptomics, proteomics, and metabolomics, providing a holistic understanding of lung cancer’s molecular landscape. Integrating detailed clinical data with molecular insights, LUNGBANK emerges as a robust foundation for advancing personalized medicine in lung cancer, allowing the identification of predictive biomarkers and treatment response indicators. This tailored approach holds the potential to optimize treatment efficacy while minimizing adverse effects, marking a significant stride in enhancing patient care. Additionally, LUNGBANK’s forward-looking strategy includes the integration of liquid biopsy materials, broadening its investigative scope and enabling a more comprehensive exploration of lung cancer dynamics. The seamless integration with classical pathology procedures further enhances LUNGBANK’s capabilities, bridging classic and contemporary research approaches for a more nuanced understanding of lung cancer. In essence, LUNGBANK’s prominence lies in its disease-specific focus, methodological richness, personalized medicine initiatives, incorporation of liquid biopsy materials, and harmonious integration with classical pathology procedures, positioning it as a pioneering force in advancing our understanding of lung cancer biology and improving the prospects for innovative and tailored therapeutic interventions.

While LUNGBANK’s specialized, project-specific orientation undoubtedly contributes depth to the field of lung cancer research, it is not immune to certain limitations concerning scope, diversity, and broad applicability when juxtaposed with the more expansive purview of both general and disease-specific biobanks. LUNGBANK’s potential shortcomings may encompass a restricted scope of research topics, a potential reduction in the diversity of data collected, and limitations in its applicability to broader medical contexts compared to the more generalized biobanks. Collaborative initiatives and strategic alliances can play a critical role in alleviating some of these weaknesses, fostering a more inclusive, diverse, and impactful research environment. Concerns regarding the limited scope may arise due to the exclusive focus on lung cancer, potentially inhibiting exploration into broader cancer research themes. Moreover, the reduced diversity in data might be a consequence of the specific patient population targeted by LUNGBANK. To address these challenges, collaborative efforts with other biobanks, both general and disease-specific, could prove beneficial in enriching the dataset and expanding the scope of research inquiries. Additionally, potential limitations such as a perceived overemphasis on molecular data, restricted comparative analyses, and challenges in securing adequate funding and support must be acknowledged. Strategic partnerships, interdisciplinary collaborations, and diversified funding sources could serve as effective mechanisms to address these challenges, ensuring LUNGBANK’s sustained relevance, and augmenting its contributions to lung cancer research and, potentially, to broader oncological investigations.

The sole lung cancer-specific biobank identified seeks to propel progress in lung cancer research, showcasing its substantial impact on early diagnosis within the realm of lung cancer (Patz et al., 2010). LUNGBANK, on the other hand, distinguishes itself as a project-specific biobank, utilizing a comprehensive multiomics approach to concurrently investigate genomics, transcriptomics, proteomics, and metabolomics. This distinctiveness is highlighted in its establishment for a specific project with a primary focus on unravelling the intricacies of lung cancer biology (Chen et al., 2023). The simultaneous examination of multiomics data derived from identical patient samples constructs a comprehensive panorama of lung cancer biology. This ample approach opens the door to the discovery of complex molecular signatures, innovative biomarkers, and promising therapeutic targets that might otherwise remain concealed when investigating each omics layer in isolation. Therefore, the accumulation of a substantial number of specimens was imperative to facilitate the investigation of various omics and related studies, all originating from identical source materials. This fundamental framework empowered us to scrutinize variations in tumors, the tumor microenvironment, and even potential metastatic processes, all in direct comparison to normal samples from the same patient. Additionally, it allowed for the assessment of these alterations in blood and other liquid biopsy materials (Rolfo et al., 2021). Moreover, the biorepository stands as a cornerstone of personalized medicine (Liu and Pollard, 2015). Armed with a wealth of clinical and molecular data, we can pinpoint predictive biomarkers and treatment response indicators that guide personalized therapeutic decisions. This individualized approach holds the promise of optimizing treatment efficacy while minimizing adverse effects (Schilsky, 2010).

In addition to its advanced capabilities, the biorepository seamlessly complements classical pathology procedures (Annaratone et al., 2021). It effortlessly facilitates the preparation of frozen and paraffin sections, revitalizing the time-honored techniques of examining tissue morphology, histology, immunohistochemistry, and in situ hybridization (Vaught and Henderson, 2011; Mohamadkhani and Poustchi, 2015; Lommen et al., 2020). These classical methodologies remain indispensable for diagnosing and characterizing lung cancer, continuing to exert a profound influence over clinical decisions. Samples from our biorepository serve as a valuable resource for the development of PDC, PDO, and PDX models for the in vitro and in vivo analyses of lung cancer biology, as well as personalized therapeutic screening (Ibarrola-Villava et al., 2018; Kim et al., 2019a; Kim et al., 2019b; Kita et al., 2019; Liu et al., 2023). These cultured entities not only epitomize the versatility and diversity within biobanking practices but also stand as a considerable reservoir of living biobanks, offering invaluable insights and opportunities for research across a spectrum of medical and scientific domains. This multifaceted approach not only reflects the dynamic nature of biobanking but also underscores the potential contributions of these cultured specimens to advancements in personalized medicine, drug discovery, and a deeper understanding of the intricacies of various diseases (Broutier et al., 2017; Ibarrola-Villava et al., 2018; Calandrini et al., 2020).

In summary, the establishment of this lung-cancer-specific biorepository implies a significant milestone in our ongoing battle against this relentless disease. It provides an exceptional resource in our endeavor to understand the intricacies of lung cancer biology, improve patient care, and develop innovative treatments. We must consistently uphold the highest standards of sample quality, ethical integrity, collaborative teamwork, and adaptability to the evolving landscape of lung cancer research to optimize its potential impact. The future holds the promise of translating discoveries from this biorepository concept into practical clinical applications, ultimately bringing hope to lung cancer patients worldwide.

Future directions can be outlined as follows. Several pivotal strategies emerge, promising to amplify LUNGBANK’s influence on lung cancer research and personalized medicine. Firstly, broadening the spectrum of collected samples, encompassing diverse patient demographics and disease subtypes, will fortify the repository, ensuring a more exhaustive comprehension of lung cancer. Collaborative endeavors with fellow biobanks hold the potential to streamline the exchange of knowledge and resources, fostering a global network dedicated to advancing lung cancer research. Strategic investments in cutting-edge technologies, such as single-cell sequencing and advanced imaging techniques, will empower LUNGBANK to delve more profoundly into the molecular intricacies of lung cancer, unveiling novel biomarkers and therapeutic targets. Aligning with real-world clinical data and outcomes will effectively bridge the divide between research findings and tangible clinical applications, ultimately enhancing patient outcomes. Proactive engagement with the broader scientific community and stakeholders will guarantee continual support, sustainability, and adherence to ethical standards. LUNGBANK’s steadfast commitment to transparency and open science can serve as an exemplary model for other biobanks, nurturing a collaborative ecosystem that expedites progress in the fight against lung cancer. Lastly, perpetual adaptation to evolving technologies and methodologies will position LUNGBANK at the vanguard of innovation, enabling it to adeptly tackle emerging challenges and make substantial contributions to the dynamic landscape of lung cancer research. In summation, these strategic directions are poised to propel LUNGBANK towards a future where its contributions transcend conventional boundaries, shaping the landscape of lung cancer research and personalized medicine.

## Supplementary Information
