# Supplementary material for: LUNGBANK: a novel biorepository strategy tailored for comprehensive multiomics analysis and P-medicine applications in lung cancer
Source: Turk J Biol. 2024 May 28;48(3):203–17. doi: 10.55730/1300-0152.2696 (PMC11265891; doi:10.55730/1300-0152.2696)
Supplement: Supplementary file 1 [file Supplementary_Data-1-SOPs.docx]

**Standard Operating Procedures (SOPs)**

**Standard Operating Procedure for Collecting Blood Samples**

**Objective:** This SOP outlines the procedure for collecting blood samples for whole genome sequencing (WGS) and total transcriptomics studies to analyse molecular markers and biomolecules in the bloodstream.

**Materials and Equipment:** Sterile blood collection tubes with anticoagulant (e.g. purple top tubes with EDTA), clean disposable gloves, permanent markers and labels, antiseptic swabs (alcohol or iodine-based), biohazard bags, ice packs or coolers, zip-lock bags or containers for transport, and phlebotomy equipment, including needles and a tourniquet.

**Procedure:**

1. Preparation: Wear clean, disposable gloves to prevent contamination. Ensure that all blood collection tubes are sterile and within their expiration dates. Label each blood collection tube with a unique identifier, date, and participant information.

2. Participant and Site Preparation: Inform participants about the blood collection procedure and obtain their informed consent. Conduct a brief medical history review and confirm that participants meet the study's inclusion criteria. Set up a clean and sterile phlebotomy area. Use antiseptic swabs to disinfect the venipuncture site, allowing it to dry before blood collection.

3. Collection: Identify a suitable vein (usually in the antecubital fossa) for venipuncture. Apply a tourniquet proximal to the venipuncture site to facilitate vein visualization. Insert a sterile needle into the vein and allow blood to flow directly into the purple top tube with EDTA. Collect an appropriate volume of blood as required for the study (e.g., 3-5 mL). Ensure that the tube is properly capped and mixed gently by inverting several times.

4. Seperation: After obtaining whole blood in the blood tube, the process of aliquoting smaller quantities can be performed directly.

5. Aliquoting: Aliquot the collected blood into sterile cryotubes (1000 µL, 500 µL, 200 µl, 100 µL, 50 µL) to accommodate different omics studies (e.g., WGS, transcriptomics).

6. Storage and Transport: Label each sterile cryotube with the participant's unique identifier and the date. Place the containers in biohazard bags to prevent potential leakage or contamination. Use ice packs or coolers to maintain proper sample temperatures during transportation (e.g., +4℃).

7. Documentation: Record the date and time of blood collection, along with relevant participant information. Maintain a comprehensive record of all collected samples, assigning a unique identifier to each one.

8. Storage at the Laboratory: Upon arrival at the laboratory, store blood samples at the -80℃ ultra-deep freezer.

9. Sample Processing: Follow the laboratory's specific protocols for the DNA and RNA isolation.

10. Reporting: Document all findings and data generated during omics analysis for future reference and publication.

11. Quality Control: Implement quality control measures throughout the process to maintain the integrity of the samples and data.

**Standard Operating Procedure for Collecting Serum Samples**

**Objective:** This SOP outlines the procedure for collecting serum samples for proteomics and metabolomics studies as well as measurement of specific molecules.

**Materials and Equipment:** Sterile clot activator serum tubes (e.g. red-top tubes), clean disposable gloves, permanent markers and labels, antiseptic swabs (alcohol or iodine-based), biohazard bags, ice packs or coolers, zip-lock bags or containers for transport, and phlebotomy equipment, including needles and a tourniquet.

**Procedure:**

1. Preparation: Wear clean, disposable gloves to prevent contamination. Ensure that all serum collection tubes are sterile and within their expiration dates. Label each serum collection tube with a unique identifier, date, and participant information.

2. Participant and Site Preparation: Inform participants about serum collection procedure and obtain their informed consent. Conduct a brief medical history review and confirm that participants meet the study's inclusion criteria. Set up a clean and sterile phlebotomy area. Use antiseptic swabs to disinfect the venipuncture site, allowing it to dry before serum collection.

3. Collection: Identified a suitable vein (usually in the antecubital fossa) for venipuncture. Applied a tourniquet proximal to the venipuncture site to facilitate vein visualization. Inserted a sterile needle into the vein and allowed blood to flow directly into the red-top tube without any anticoagulants. Collected an appropriate volume of blood as required for the study (e.g., 3-5 mL). Ensured that the red-top tube was properly capped.

4. Clotting and Separation: Allow the collected blood to clot by leaving the red-top tubes undisturbed at room temperature for about 5 minute. After clotting, centrifuge the tubes at 4000xg speed and 7 minutes duration to separate the serum from the clot. Transfer the serum portion into a clean, labeled tube.

5. Aliquoting: Aliquot the collected serum into sterile cryotubes (1000 µL, 500 µL, 200 µl, 100 µL, 50 µL) to accommodate different omics studies (e.g., proteomics, metabolomics).

6. Storage and Transport: Label each sterile cryotube with the participant's unique identifier and the date. Place the containers in biohazard bags to prevent potential leakage or contamination. Use ice packs or coolers to maintain proper sample temperatures during transportation (e.g., +4℃).

7. Documentation: Record the date and time of serum collection, along with relevant participant information. Maintain a comprehensive record of all collected samples, assigning a unique identifier to each one.

8. Storage at the Laboratory: Upon arrival at the laboratory, store serum samples at the appropriate temperature according to the lab's specific storage protocols (e.g., -80℃).

9. Sample Processing: Follow the laboratory's specific protocols for serum sample preparation and proteomics/metabolomics analysis.

10. Reporting: Document all findings and data generated during omics analysis for future reference and publication.

11. Quality Control: Implement quality control measures throughout the process to maintain the integrity of the samples and data.

**Standard Operating Procedure for Collecting Plasma Samples**

**Objective:** This SOP outlines the procedure for collecting plasma samples for proteomics and metabolomics studies as well as measurement of specific molecules.

**Materials and Equipment:** To ensure safe and efficient plasma collection, the following equipment is required: sterile plasma collection tubes with anticoagulant (e.g. green top tubes with heparin), clean disposable gloves, permanent markers and labels, antiseptic swabs (alcohol or iodine-based), biohazard bags, ice packs or coolers, zip-lock bags or containers for transport, and phlebotomy equipment, including needles and a tourniquet.

**Procedure:**

1. Preparation: Wear clean, disposable gloves to prevent contamination. Ensure that all plasma collection tubes are sterile and within their expiration dates. Label each plasma collection tube with a unique identifier, date, and participant information.

2. Participant and Site Preparation: Inform participants about the plasma collection procedure and obtain their informed consent. Conduct a brief medical history review and confirm that participants meet the study's inclusion criteria. Set up a clean and sterile phlebotomy area. Use antiseptic swabs to disinfect the venipuncture site, allowing it to dry before plasma collection.

3. Collection: Identify a suitable vein (usually in the antecubital fossa) for venipuncture. Apply a tourniquet proximal to the venipuncture site to facilitate vein visualization. Insert a sterile needle into the vein and allow blood to flow directly into the green top tube with heparin. Collect an appropriate volume of blood as required for the study (e.g., 5-10 mL). Ensure that the tube is properly capped and mixed gently by inverting several times.

4. Seperation: Centrifuge the green-top tube at 4000xg speed and 7 minutes duration to separate the plasma from the cellular components. Transfer the plasma portion into a clean, labeled tube, taking care to avoid any cell debris or red blood cell contamination.

5. Aliquoting: Aliquot the collected plasma into sterile cryotubes (1000 µL, 500 µL, 200 µl, 100 µL, 50 µL) to accommodate different omics studies (e.g., proteomics, metabolomics).

6. Storage and Transport: Label each sterile cryotube with the participant's unique identifier and the date. Place the containers in biohazard bags to prevent potential leakage or contamination. Use ice packs or coolers to maintain proper sample temperatures during transportation (e.g., +4℃).

7. Documentation: Record the date and time of plasma collection, along with relevant participant information. Maintain a comprehensive record of all collected samples, assigning a unique identifier to each one.

8. Storage at the Laboratory: Upon arrival at the laboratory, store plasma samples at the appropriate temperature according to the lab's specific storage protocols (e.g., -80℃).

9. Sample Processing: Follow the laboratory's specific protocols for plasma sample preparation and proteomics/metabolomics analysis.

10. Reporting: Document all findings and data generated during omics analysis for future reference and publication.

11. Quality Control: Implement quality control measures throughout the process to maintain the integrity of the samples and data.

**Standard Operating Procedure for Collecting Urine Samples**

**Objective:** This SOP outlines the procedure for collecting urine samples for the future omics studies to analyse molecular markers and biomolecules present in urine.

**Materials and Equipment:** To ensure safe and efficient urine collection, the following equipment is required: clean disposable gloves, sterile urine collection containers, labels and pens for marking, biohazard containers, ice packs or coolers, zip-lock bags or containers for transport.

**Procedure:**

1. Preparation: Wear clean, disposable gloves to prevent contamination. Ensure that all urine collection containers are sterile and labeled with a unique identifier, date, and participant information.

2. Participant Preparation: Participants are provided with explicit instructions on how to collect the urine sample, including precise details on the timing of collection and any necessary dietary or activity restrictions if applicable, ensuring that they fully comprehend the entire process.

3. Collection: Participants collect the urine sample in a sterile container according to the provided instructions. Instruct participants to avoid collecting the first urine of the day (first morning void) and to collect a midstream urine sample for better representation. Collect an appropriate volume of urine as required for the study (e.g., 30-50 mL). Ensure that the container is tightly sealed to prevent leakage or contamination.

4. Storage and Transport: Place each urine collection container in a biohazard bag to prevent any potential leakage or contamination. For transportation, place the biohazard bag inside a zip-lock bag or a secondary container. Use ice packs or coolers to maintain the samples at a cool temperature during transportation (e.g., +4℃).

5. Documentation: Record the date and time of urine collection, along with relevant participant information. Maintain a detailed record of the samples collected, including unique identifiers for each sample.

6. Storage at the Laboratory: Upon arrival at the laboratory, store urine samples at the appropriate temperature according to the lab's specific storage protocols (e.g., -80℃).

7. Sample Processing: Follow the laboratory's specific protocols for urine sample preparation and omics analysis, whether it's genomics, transcriptomics, proteomics, or metabolomics.

8. Reporting: Document all findings and data generated during omics analysis for future reference and publication.

9. Quality Control: Implement quality control measures throughout the process to maintain the integrity of the samples and data.

**Standard Operating Procedure for Collecting Sputum Samples for Omics Studies**

**Objective:** This SOP outlines the procedure for collecting sputum samples for omics studies, including genomics, transcriptomics, proteomics, or metabolomics to analyze molecular markers and biomolecules present in sputum.

**Materials and Equipment:** To ensure safe and efficient sputum collection, the following equipment is required: clean and disposable gloves**,** sterile sputum collection containers**,** labels and permanent markers**,** biohazard bags**,** saline solution (0.9% sterile saline)**,** sputum induction equipment (if needed)**,** ice packs or coolers**,** zip-lock bags or containers for transportation.

**Procedure:**

1. Preparation: Wear clean, disposable gloves to prevent contamination. Ensure that all sputum collection containers are sterile and labeled with a unique identifier, date, and participant information.

2. Participant Instructions: Provide participants with clear instructions on how to collect the sputum sample. Include information on the timing of collection and any dietary or activity restrictions if applicable. Ensure they understand the process.

3. Collection: Participants collect the sputum sample according to the provided instructions. If necessary, sputum induction equipment is used following the manufacturer's instructions. Sterile saline solution (0.9%) is administered via a nebulizer to facilitate sputum production. Collect an appropriate volume of sputum as required for the study (e.g., 1-2 mL). Ensure that the sputum collection container is tightly sealed to prevent leakage or contamination.

4. Storage and Transport: Placed each sputum collection container in a biohazard bag to prevent any potential leakage or contamination. If necessary for transportation, placed the biohazard bag inside a zip-lock bag or a secondary container. Used ice packs or coolers with ice packs to maintain the samples at a cool temperature during transportation (e.g., +4℃).

5. Documentation: Record the date and time of sputum sample collection, along with relevant participant information. Maintain a detailed record of the samples collected, including unique identifiers for each sample.

6. Storage at the Laboratory: Upon arrival at the laboratory, store sputum samples at the appropriate temperature according to the lab's specific storage protocols (e.g., -80℃).

7. Sample Processing: Follow the laboratory's specific protocols for sputum sample preparation and omics analysis, whether it's genomics, transcriptomics, proteomics, or metabolomics.

8. Reporting: Document all findings and data generated during omics analysis for future reference and publication.

9. Quality Control: Implement quality control measures throughout the process to maintain the integrity of the samples and data.

**Standard Operating Procedure for Collecting Saliva Samples for Metagenomics Studies**

**Objective:** This SOP outlines the procedure for collecting saliva samples for metagenomics studies to analyze the microbial composition of the oral microbiome.

**Materials and Equipment:** To ensure safe and efficient saliva collection, the following equipment is required: clean and disposable gloves**,** saliva collection tubes or containers with stabilizing solution**,** labels and permanent markers**,** timer or clock**,** biohazard bags**,** ice packs or coolers, zip-lock bags or containers for transportation.

**Procedure:**

1. Preparation: Wear clean, disposable gloves to prevent contamination. Ensure that all saliva collection containers are sterile and labeled with a unique identifier, date, and participant information.

2. Participant Instructions: Provide participants with clear instructions on how to collect the saliva sample. Include information on the timing of collection and any dietary or activity restrictions if applicable. Ensure they understand the process.

3. Collection: Participants follow the provided instructions to collect the saliva sample. Instruct participants to avoid eating, drinking, or using mouthwash at least 30 minutes before collection. Ask participants to drool directly into the collection tube or container with the stabilizing solution, making sure not to touch the inside of the container with their lips or tongue. Collect an appropriate volume of saliva as required for the study (e.g., 1-2 mL). Ensure that the saliva collection tube or container is tightly sealed to prevent leakage or contamination

4. Timer: Monitor the time to ensure that participants collected saliva for the specified duration (e.g., 5 minutes).

5. Storage and Transport: Placed each saliva collection container in a biohazard bag to prevent any potential leakage or contamination. If necessary for transportation, placed the biohazard bag inside a zip-lock bag or a secondary container. Used ice packs or coolers with ice packs to maintain the samples at a cool temperature during transportation (e.g., +4℃).

6. Documentation: Record the date and time of saliva sample collection, along with relevant participant information. Maintain a detailed record of the samples collected, including unique identifiers for each sample.

7. Storage at the Laboratory: Upon arrival at the laboratory, store saliva samples at the appropriate temperature according to the lab's specific storage protocols (e.g., -80℃).

8. Sample Processing: Follow the laboratory's specific protocols for saliva sample preparation and metagenomics or metatranscriptomics analysis.

9. Reporting: Document all findings and data generated during omics analysis for future reference and publication.

10. Quality Control: Implement quality control measures throughout the process to maintain the integrity of the samples and data.

**Standard Operating Procedure for Collecting Saliva Samples for Omics Studies**

**Objective:** This SOP outlines the procedure for collecting saliva samples for omics studies, including genomics, transcriptomics, proteomics, or metabolomics to analyze molecular markers and biomolecules present in saliva.

**Materials and Equipment:** To ensure safe and efficient saliva collection, the following equipment is required: clean and disposable gloves**,** saliva collection tubes or containers with stabilizing solution**,** labels and permanent markers**,** timer or clock**,** biohazard bags**,** ice packs or coolers, zip-lock bags or containers for transportation.

**Procedure:**

1. Preparation: Wear clean, disposable gloves to prevent contamination. Ensure that all saliva collection containers are sterile and labeled with a unique identifier, date, and participant information.

2. Participant Instructions: Provide participants with clear instructions on how to collect the saliva sample. Include information on the timing of collection and any dietary or activity restrictions if applicable. Ensure they understand the process.

3. Collection: Participants follow the provided instructions to collect the saliva sample. Instruct participants to avoid eating, drinking, or brushing their teeth at least 30 minutes before collection. They rinse their mouth twice with water and wait 30 minutes. Ask participants to spit directly into the collection tube or container, making sure not to touch the inside of the container with their lips or tongue. Collect an appropriate volume of saliva as required for the study (e.g., 1-2 mL). Ensure that the saliva collection tube or container is tightly sealed to prevent leakage or contamination.

4. Timer: Monitor the time to ensure that participants collected saliva for the specified duration (e.g., 5 minutes).

5. Storage and Transport: Placed each saliva collection container in a biohazard bag to prevent any potential leakage or contamination. If necessary for transportation, placed the biohazard bag inside a zip-lock bag or a secondary container. Used ice packs or coolers with ice packs to maintain the samples at a cool temperature during transportation (e.g., +4℃).

6. Documentation: Record the date and time of saliva sample collection, along with relevant participant information. Maintain a detailed record of the samples collected, including unique identifiers for each sample.

7. Storage at the Laboratory: Upon arrival at the laboratory, store saliva samples at the appropriate temperature according to the lab's specific storage protocols (e.g., -80℃).

8. Sample Processing: Follow the laboratory's specific protocols for saliva sample preparation and omics analysis, whether it's genomics, transcriptomics, proteomics, or metabolomics.

9. Reporting: Document all findings and data generated during omics analysis for future reference and publication.

10. Quality Control: Implement quality control measures throughout the process to maintain the integrity of the samples and data.

**Standard Operating Procedure for Collecting Bronchoalveolar Lavage (BAL) Fluid during Surgery for Omics Studies**

**Objective:** This SOP outlines the procedure for collecting bronchoalveolar lavage (BAL) fluid during surgery for omics studies, including genomics, transcriptomics, proteomics, or metabolomics to analyze molecular markers and biomolecules in the respiratory system.

**Materials and Equipment:** To ensure safe and efficient BAL fluid collection, the following equipment is required: **c**lean and disposable gloves**,** sterile collection containers for BAL fluid**,** sterile saline solution (0.9% sodium chloride)**,** labels and permanent markers**,** specimen cups for aliquoting**,** biohazard bags**,** ice packs or coolers**,** zip-lock bags or containers for transportation**,** surgical equipment and drapes**,** endotracheal tube and ventilator**,** anesthesia equipment and medications**.**

**Procedure:**

1. Pre-Surgery Preparation: Wear clean and disposable gloves to prevent contamination. Ensure that all BAL fluid collection containers are sterile and labeled with a unique identifier, date, and participant information. Verify the availability of all necessary surgical equipment.

2. Participant Preparation: Inform participants about the surgical procedure, risks and benefits, and obtaining their informed consent. Conduct a thorough preoperative assessment to ensure participant suitability for the procedure.

3. Anesthesia and Intubation: Administer anesthesia as per the anesthesia team's protocol. Place the participant under general anesthesia and ensure endotracheal intubation and mechanical ventilation to maintain oxygenation during the surgery.

4. Surgical Procedure: Conduct the surgery as per the surgical team's protocol. At the appropriate surgical phase, introduce a sterile bronchoscope through the endotracheal tube. Instill a specified volume (e.g., 50-100 mL) of sterile saline solution (0.9% sodium chloride) through the bronchoscope into the target bronchus. Aspirate the BAL fluid, containing cells and secretions, back into a sterile collection container. Repeat the lavage as needed to obtain the desired volume of BAL fluid.

5. Aliquoting: Aliquot the collected BAL fluid into sterile specimen cups or containers as needed for different omics studies (e.g., genomics, transcriptomics, proteomics, or metabolomics).

6. Storage and Transport: Placed each BAL fluid collection container in a biohazard bag to prevent any potential leakage or contamination. If necessary for transportation, placed the biohazard bag inside a zip-lock bag or a secondary container. Used ice packs or coolers with ice packs to maintain the samples at a cool temperature during transportation (e.g., +4℃).

7. Documentation: Record the date and time of BAL fluid sample collection, along with relevant participant information. Maintain a detailed record of the samples collected, including unique identifiers for each sample.

8. Storage at the Laboratory: Upon arrival at the laboratory, store BAL fluid samples at the appropriate temperature according to the lab's specific storage protocols (e.g., -80℃).

9. Sample Processing: Follow the laboratory's specific protocols for BAL fluid sample preparation and omics analysis, whether it's genomics, transcriptomics, proteomics, or metabolomics.

10. Reporting: Document all findings and data generated during omics analysis for future reference and publication.

11. Quality Control: Implement quality control measures throughout the process to maintain the integrity of the samples and data.

**Standard Operating Procedure for RNAse-Free Homogenization of Lung Tissue Samples for Omics Studies Using a Mortar and Pestle**

**Objective:** This SOP outlines the procedure for homogenizing lung tissue samples into a powder format suitable for omics studies, including genomics, transcriptomics, proteomics, and metabolomics using a mortar and pestle in a sterile and RNAse-free environment.

**Materials and Equipment:** To ensure safe and efficient lung tissue sample collection, the following equipment is required: clean and disposable gloves, mortar and pestle (sterile and pre-treated for RNAse-free conditions), liquid nitrogen (for cryogenic homogenization), lab coat, safety goggles, face mask (for personal protection), tubes or containers for collecting the powdered tissue samples, permanent markers (RNAse-free) RNAse-free pipettes and tips, RNAse-free microcentrifuge tubes, biohazard bags, ice packs or coolers.

**Procedure:**

1. Preparation: Wear appropriate personal protective equipment (PPE), including a lab coat, safety goggles, and a face mask, in a designated RNAse-free workspace (e.g., a laminar flow hood or RNAse-free laboratory). Ensure that the mortar and pestle, as well as all other equipment, are RNAse-free and properly treated or sterilized. Prepare the RNAse-free homogenization buffer or solution required for specific omics study. All solutions are prepared using RNAse-free reagents and containers.

2. Sample Retrieval: Retrieve the lung tissue samples from storage at the appropriate temperature (e.g., -80℃) in the RNAse-free environment. Place the samples in RNAse-free containers in liquid nitrogen to maintain their integrity during the homogenization process.

3. Cryogenic Homogenization: Add a small amount of liquid nitrogen to the RNAse-free mortar to pre-chill it. Place a small portion of RNAse-free lung tissue into the mortar while still keeping the remaining tissue in liquid nitrogen. Use the RNAse-free pestle to crush the tissue while adding liquid nitrogen as needed to maintain a powder-like consistency. Repeat steps 2 and 3 until all RNAse-free tissue samples are processed into a fine powder.

5. Collection of Powdered Tissue: Transfer the RNAse-free powdered tissue into labeled ice-cold RNAse-free cryotubes.

6. Homogenization Buffer/Solution: Add an appropriate volume of RNAse-free homogenization buffer or solution to the powdered tissue samples as required. Use RNAse-free pipettes and tips for this step.

7. Storage and Transportation: Label each RNAse-free cryotube with a unique identifier, date, and study information using RNAse-free labels and permanent markers. Place the RNAse-free cryotubes in liquid nitrogen tank for transportation. Store the RNAse-free samples at the appropriate temperature or as specified for specific omics analysis (e.g., -80℃) in the RNAse-free environment.

8. Documentation: Record the date, time, and details of the RNAse-free homogenization process, including the volume of RNAse-free homogenization buffer or solution used.

9. Sample Processing: Follow the lab's specific protocols for further sample processing and omics analysis, whether it's genomics, transcriptomics, proteomics, or metabolomics or in the RNAse-free environment.

10. Reporting: Document all findings and data generated during omics analysis for future reference and publication.

11. Quality Control: Implement quality control measures throughout the process to maintain the integrity of the samples and data.

**Standard Operating Procedure for Characterizing DNA for WGS Studies Using Qubit Fluorometer,** **Nanodrop Spectrophotometer, and Bioanalyzer**

**Objective:** This SOP outlines the procedure for characterizing DNA obtained from solid or liquid specimens for omics studies using three different methods: Qubit fluorometer and nanodrop spectrophotometer.

**Materials and Equipment:** To ensure safe and efficient lung tissue samples collection, the following equipment is required: DNA samples**,** qubit fluorometer and qubit DNA assay kit**,** nanodrop spectrophotometer**,** clean and disposable gloves**,** DNA-free microcentrifuge tubes and pipette tips**,** ice packs or coolers.

**Procedure:**

1. Preparation: Ensure that all equipment and consumables are clean and properly calibrated. Wear clean, disposable gloves to prevent contamination. Prepare DNA samples in DNAse-free microcentrifuge tubes.

2. DNA Concentration Measurement using Qubit Fluorometer: Set up the qubit fluorometer according to the manufacturer's instructions. Prepare the working solution of the qubit DNA assay kit. Label two DNAse-free microcentrifuge tubes as "Standard 1" and "Standard 2." Add 190 μL of the working solution to the "Standard" tube. Load 10 μL of each Standard sample into separate microcentrifuge tubes. Add 198 μL of the working solution to the "Sample" tubes and load 2 μL of each DNA sample, ensuring no contamination between samples. Mix the "Sample" tube gently by vortexing and allow it to incubate for 2 minutes. Measure the DNA concentration using the qubit fluorometer and record the results.

3. Checking DNA purity using a Nanodrop Spectrophotometer: Place 1 μL of the DNA sample on a specialized pedestal or sample holder within the nanodrop spectrophotometer. DNA molecules in the sample absorb ultraviolet (UV) light at a specific wavelength, typically around 260 nanometers (nm). The instrument measures the amount of light absorbed by the sample at this wavelength. The spectrophotometer's software calculates the concentration of DNA in the sample based on the amount of UV light absorbed. Record the DNA purity levels as 280/260 and 260/230 values.

4. Documentation: Record all data from the Qubit and Nanodrop analyses, including DNA concentrations and purity details.

5. Storage and Transportation: Store DNA samples at the appropriate temperature and conditions to maintain their integrity. If necessary for transportation, place the samples in DNA-free microcentrifuge tubes inside a container with ice packs or coolers.

6. Reporting: Report the DNA concentration and purity details, for each sample.

7. Quality Control: Implement quality control measures throughout the process to ensure the accuracy and reliability of the DNA characterizations and sequencing.

**Standard Operating Procedure for RNA Characterization for Transcriptomics Studies Using Qubit Fluorometer, Nanodrop Spectrophotometer, and Bioanalyzer in an RNAse-Free Environment**

**Objective:** This SOP outlines the procedure for characterizing RNA obtained from solid or liquid specimens for omics studies using three different methods: Qubit fluorometer, Nanodrop spectrophotometer, and Bioanalyzer electrophoresis in an RNAse-free environment.

**Materials and Equipment:** To ensure safe and efficient lung tissue samples collection, the following equipment is required: RNA samples**,** qubit fluorometer and qubit RNA assay kit**,** nanodrop spectrophotometer**,** bioanalyzer instrument and RNA analysis kit**,** clean and disposable gloves**,** RNAse-free microcentrifuge tubes and pipette tips**,** ice packs or coolers, RNAse-free laboratory setup (e.g., laminar flow hood, RNAse-free reagents).

**Procedure:**

1. Preparation in an RNAse-Free Environment: Wear clean and disposable gloves in a designated RNAse-free workspace. Ensure that all equipment, consumables, and reagents are RNAse-free and properly treated or sterilized. Prepare RNA samples in RNAse-free microcentrifuge tubes using RNAse-free pipette tips and reagents.

2. RNA Concentration Measurement using Qubit Fluorometer : Set up the Qubit fluorometer according to the manufacturer's instructions. Prepare the working solution of the Qubit RNA assay kit. Label two RNAse-free microcentrifuge tubes as "Standard 1" and "Standard 2." Add 190 μL of the working solution to the "Standard" tube. Load 10 μL of each Standard sample into separate microcentrifuge tubes. Add 198 μL of the working solution to the "Sample" tubes and load 2 μL of each RNA sample, ensuring no contamination between samples. Mix the "Sample" tube gently by vortexing and allow it to incubate for 2 minutes. Measure the RNA concentration using the Qubit fluorometer and record the results.

3. RNA Quality Check using Bioanalyzer: Set up the instrument according to the manufacturer's instructions. Prepare RNA analysis chips and gel-dye solution from the RNA analysis kit. Before opening the chip, load 2 μL of each RNA sample into separate microcentrifuge tubes and store them on ice. First, open the chip, then add 9 μL of gel-dye solution to the first G area. Following that, use a syringe according to the manufacturer's instructions to load it into each of the other G areas on the chip. Load 5 μL of reference solution into each separate bioanalyzer chip. Load 1 μL of each RNA sample into each separate Bioanalyzer chip. Add 1 μL marker into the ladder area. Run the Bioanalyzer analysis according to the manufacturer's instructions. Evaluate the RNA quality based on the electropherogram and record the results.

4. Checking RNA purity using a Nanodrop Spectrophotometer: Place 1 μL of the RNA sample on a specialized pedestal or sample holder within the Nanodrop spectrophotometer. RNA molecules in the sample absorb ultraviolet (UV) light at a specific wavelength, typically around 260 nanometers (nm). The instrument measures the amount of light absorbed by the sample at this wavelength. The spectrophotometer's software calculates the concentration of RNA in the sample based on the amount of UV light absorbed. Record the RNA purity levels as 280/260 and 260/230 values.

5. Documentation: Record all data from the Qubit, Bioanalyzer, and Nanodrop analyses, including RNA concentrations, quality assessments, and purity details.

6. Storage and Transportation: Store RNA samples at the appropriate temperature and conditions to maintain their integrity. Place the samples in RNA-free microcentrifuge tubes inside a container with ice packs or coolers.

7. Reporting: Report the RNA concentration and quality results, along with purity details, for each sample.

8. Quality Control: Implement quality control measures throughout the process to ensure the accuracy and reliability of RNA characterizations and sequencing in the RNAse-free environment.

**Standard Operating Procedure for Characterizing the Suitability of Tissue Specimens for Proteomics and Phosphoproteomics Studies Using LC-MS/MS**

**Objective:** This SOP outlines the procedure for assessing the suitability of solid or liquid tissue specimens for proteomics and phosphoproteomics studies through Liquid Chromatography-Mass Spectrometry (LC-MS/MS). It includes criteria for sample quality, quantity, and pre-processing steps.

**Materials and Equipment:** To ensure safe and efficient lung tissue samples collection, the following equipment is required: tissue specimens (powder form)**,** clean and disposable gloves**,** precision balance**,** liquid nitrogen, protein extraction buffer**,** phosphatase inhibitor (if studying phosphoproteomics)**,** microcentrifuge tubes**,** labels and permanent markers, FASP protein digestion kit, probe sonicator, Qubit 4 Fluorometer, NanoDrop Spectrophotometer, LC-MS/MS instrument**,** ice packs or coolers.

**Procedure:**

1. Sample Preparation: Retrieve the lung tissue powder samples from storage at the appropriate temperature (e.g., -80℃). Place the samples in liquid nitrogen to maintain their integrity during weighing.

2. Sample Quantity Assessment: Weigh the tissue homogenate using an appropriate balance. Assess whether the tissue powder amount meets the necessary quantity for proteomics or phosphoproteomics analysis. After determining the appropriate amount, aliquot the samples into sterile cryotubes as specified.

3. Protein Extraction: Begin the procedure immediately after removing the powdered tissue samples that have been aliquoted from the freezer. Add 500 µL of lysis buffer to the tissue powder. Apply probe sonication while maintaining the sample on ice. Set the probe sonicator parameters as follows: 3 cycles of 15 seconds "On" and 5 seconds "Off" at 40% power. After sonication, centrifuge the sample at 14,000 rpm for 20 minutes to separate the pellet. Carefully transfer the supernatant containing the extracted proteins to a new tube, taking care not to disturb the pellet. Determine the protein concentration using a Qubit 4 Fluorometer. Ensure that all steps are performed on ice or at low temperatures to prevent protein degradation. Properly label all tubes and samples to avoid cross-contamination.

4. Enzymatic Digestion: The preparation and processing of protein extracts for LC-MS/MS analysis necessitate trypsin digestion. To generate peptides, employ the FASP^TM^ Protein Digestion Kit from Abcam (ab270519), following the manufacturer's protocol. Digest the protein through an overnight incubation at 37°C in the presence of sequencing-grade trypsin, with a protein-to-enzyme ratio of 25:1. Assess the peptide concentration using a NanoDrop Spectrophotometer at A280 and transfer an adequate quantity of the samples into vials for the LC-MS/MS analysis.

5. Documentation: Record all data, including powder weight and quality assessment.

6. Storage and Transportation: Store suitable tissue powder specimens at the appropriate temperature (e.g.,-80℃) for long-term storage. If necessary for transportation, place suitable specimens in microcentrifuge tubes inside a container with ice packs or coolers.

7. Reporting: Document the suitability assessment results and report them as part of the sample metadata for downstream proteomics or phosphoproteomics analyses

8. Quality Control: Implement quality control measures throughout the process to ensure the integrity of the tissue specimens and data.

**Standard Operating Procedure for Characterizing the Suitability of Tissue Specimens for Metabolomics and Lipidomics Studies Using Q-TOF LC-MS/MS**

**Objective:** This SOP outlines the procedure for assessing the suitability of solid or liquid tissue specimens for metabolomics and lipidomics studies through Time of Flight Liquid Chromatography-Mass Spectrometry (Q-TOF LC-MS/MS). It includes criteria for sample quality, quantity, and pre-processing steps.

**Materials and Equipment:** To ensure safe and efficient lung tissue samples collection, the following equipment is required: tissue specimens (powder form)**,** clean and disposable gloves**,** precision balance**,** liquid nitrogen**,** metabolite extraction solution**,** lipid extraction solution**,** microcentrifuge tubes**,** labels and permanent markers**,** Q-TOF LC-MS/MS instrument**,** ice packs or coolers.

**Procedure:**

1. Sample Preparation: Retrieve the lung tissue powder samples from storage at the appropriate temperature (e.g., -80℃). Place the samples in liquid nitrogen to maintain their integrity during weighing.

2. Sample Quantity Assessment: Weigh the tissue homogenate using an appropriate balance. Assess whether the tissue powder amount meets the necessary quantity for metabolomics and lipidomics analysis. After determining the appropriate amount, aliquot the samples into sterile cryotubes as specified.

3. Metabolite/lipid Extraction: Thaw the required tissue samples in an iced medium. After that 1.0 mL of ethanol were transferred into cryotube. Tissue samples were homogenized with zirconium beads. Samples were kept in an ultrasonic bath for lysation about ten minutes. Centrifugation were performed for 7 minutes and 10.000xg. Supernatant were extracted and evaporated under the vacuum. Samples were reconstituted just before the Q-TOF analysis with the mobile phase.

4. Quality control samples: Take 10 µL of each extracted sample and collect them in a pool for quality control studies. This pooled QC sample was injected every ten run to normalize the whole analysis. These samples were also used to filter ghost peaks along the analysis.

5. Documentation: Record all data, including powder weight and quality assessments.

6. Storage and Transportation: Store suitable tissue powder specimens at the appropriate temperature (e.g., -80℃) for long-term storage. If necessary for transportation, place suitable specimens in microcentrifuge tubes inside a container with ice packs or coolers.

7. Reporting: Document the suitability assessment results and report them as part of the sample metadata for downstream metabolomics and lipidomics analyses.

8. Quality Control: Implement quality control measures throughout the process to ensure the integrity of the tissue specimens and data.

**Standard Operating Procedure for Patient-Derived Cell (PDC) or Organoid (PDO) Isolation from Lung Cancer Specimens**

**Objective:** This SOP outlines the procedure for isolating patient-derived cells (PDCs) from lung cancer specimens for research or diagnostic purposes.

**Materials and Equipment:** To ensure safe and efficient lung tissue samples collection, the following equipment is required: lung cancer tissue specimen**,** sterile phosphate-buffered saline (PBS)**,** Miltenyi tumor dissociation kit**,** sterile tissue culture medium (e.g., RPMI-1640)**,** penicillin/streptomycin**,** glutamine**,** sterile disposable scalpels and forceps**,** sterile petri dishes**,** tissue culture flasks**,** laminar flow hood**,** water bath, sterile pipettes and pipette tips**,** 70-µM sterile cell strainer, centrifuge**,** sterile microcentrifuge tubes**,** trypan blue (optional)**,** hemocytometer or automated cell counter**,** CO_2_ incubator**,** liquid nitrogen storage, flow cytometry, F-actin stain equipment, DAPI, CD44 and CD45 surface antibodies, growth factors (e.g., EGF, Noggin, R-spondin), ROCK inhibitor (e.g., Y-27632), and Matrigel or extracellular matrix (ECM) for organoid culture.

**Procedure:**

1. Preparatory Steps: Ensure that all equipment, materials, and the working environment are sterile and appropriate for cell culture. Obtain the lung cancer tissue specimen as per ethical guidelines and informed consent.

2. Tissue Dissection and Enzymatic Digestion: Using sterile scalpels and forceps, dissect the lung cancer tissue specimen into small pieces (approximately 1-2 mm³) in a sterile Petri dish. The Miltenyi tumor dissociation kit is being used for isolation. The enzyme cocktail contained in the kit is prepared according to the manufacturer's instructions and added to the cleaned tumor tissue. The tissue in the enzyme cocktail solution is cut into small pieces with a sterile scalpel and minced.

4. Cell Isolation: Then, the tissue homogenate is transferred to a sterile 15-ml Falcon tube and incubates in a water bath at 37℃ for 60 minutes (shaking every 15 minutes). After incubation, the digested tissue is filtered through a 70-µm sterile cell strainer, centrifuged at 300×g for 5 minutes, and resuspended in a mixture of RPMI 1640 medium (Gibco) containing 50 IU/ml penicillin, streptomycin, and 4 mM glutamine. Finally, the isolated cells are seeded in 75 cm^2^ cell culture flasks where they grow and proliferate at 37℃ in a humidified incubator containing 5% CO_2_.

5. Cell Viability and Quality Assessment: Mix a small aliquot of the cell suspension with trypan blue and load it onto a hemocytometer or an automated cell counter. Count the viable cells to assess cell viability. The percentage of viable cells should be determined. The F-actin stain protocol for cell morphology and the Flow cytometry protocol for CD44 and CD45 are applied according to the manufacturer's guidelines for quality control.

6. Cell Culture and Organoid Culture: If you intend to culture PDCs, seed the isolated cells into tissue culture flasks or plates using an appropriate tissue culture medium supplemented with penicillin-streptomycin. Incubate the cells at 37℃ with 5% CO_2_ in a humidified incubator. If you intend to culture PDOs, mix the isolated cells with Matrigel or extracellular matrix and seed them into tissue culture flasks or plates using an appropriate culture medium supplemented with penicillin-streptomycin, growth factors, and ROCK inhibitor. Incubate the cells at 37°C with 5% CO_2_ in a humidified incubator.

8. Cryopreservation and Documentation: If necessary, aliquot the isolated PDCs and PDOs into cryovials in (90%FBS+10% DMSO), and store the vials in liquid nitrogen for long-term preservation. Record details of the isolation process, including tissue source, digestion time, cell yield, and viability (if assessed).

10. Quality Control: Implement quality control measures to monitor the health and stability of the PDCs and PDOs during culture, if applicable.

**Standard Operating Procedure for Establishing Patient-Derived Xenograft (PDX) Models from Lung Cancer Specimens**

**Objective:** This SOP outlines the procedure for establishing patient-derived xenograft (PDX) models from lung cancer tumor specimens for preclinical research purposes.

**Materials and Equipment:** To ensure safe and efficient lung tissue samples collection, the following equipment is required: lung cancer tissue specimen**,** immune-deficient mice (e.g., NOD/SCID or NSG mice)**,** sterile phosphate-buffered saline (PBS)**,** sterile tissue culture medium (e.g., RPMI-1640 or DMEM)**,** sterile surgical tools (scissors, forceps, sterile drapes)**,** sterile disposable scalpel blades and handles**,** isoflurane anesthesia setup**,** sterile sutures and surgical clips**,** sterile cotton swabs and alcohol pads**,** surgical instruments for tumor transplantation**,** animal husbandry equipment (cages, bedding, food, water)**,** autoclave for sterilization**,** animal monitoring equipment (e.g., scales).

**Procedure:**

1. Preparatory Steps: Ensure that all equipment, materials, and the working environment are sterile and appropriate for animal experiments. Obtain the lung cancer tissue specimen as per ethical guidelines and informed consent.

2. Preparation of Mice: Use immune-deficient mice (e.g., NOD/SCID or NSG) that are age and sex-matched for the experiments. House the mice in a controlled environment with regulated temperature, humidity, and a 12-hour light/dark cycle. Provide sterile bedding, food, and water ad libitum.

3. Anesthesia: Induce anesthesia in the recipient mouse using an isoflurane anesthesia setup, following the manufacturer's instructions.

4. Tumor Implantation: Using sterile surgical tools, make a small incision on the mouse's flank. Place the lung tumor tissue specimen, previously dissected into small pieces (1-2 mm³), into the incision site. Suture the incision closed or secure it with surgical clips. Ensure that the surgical area is disinfected with alcohol swabs before closing.

5. Post-surgery Care: Monitor the recipient mice closely during the post-surgery period for signs of distress or complications. Provide appropriate post-operative care, including pain relief if necessary.

6. Tumor Growth Monitoring: Regularly monitor the implanted mice for tumor growth by palpating the implantation site and recording tumor size. Measure tumor size using calipers or similar tools and record measurements regularly.

7. Collection of PDX Tumor Samples: When the tumors reach an appropriate size or as dictated by the experimental timeline, euthanize the mice using an approved method (e.g., CO_2_ asphyxiation). Harvest the PDX tumors for further analysis, such as molecular characterization or drug testing.

8. Documentation: Maintain detailed records of all aspects of the PDX model establishment, including donor tumor characteristics, implantation details, and tumor growth monitoring.

9. Quality Control: Implement quality control measures to ensure the reliability and reproducibility of PDX model experiments.

10. Ethical Considerations: Adhere to ethical guidelines and institutional protocols for working with animals.

**Standard Operating Procedure for Collecting Stool Samples for Metagenomics Studies**

**Objective:** This SOP outlines the procedure for collecting stool samples for metagenomics studies to analyze the microbial composition of the gut microbiome.

**Materials and Equipment:** Clean, disposable gloves**,** Sterile collection containers (e.g., sterile plastic vials or tubes)**,** Ice packs or coolers with ice packs**,** Ziplock bags or containers for transportation, Biohazard bags, Labels and permanent markers**,** Disposable scoops or spatulas**,** Disposable plastic bags or wrap.

**Procedure:**

1. Preparatory Steps: Wear clean and disposable gloves to prevent contamination. Label each collection container with a unique identifier, date, and participant information.

2. Participant Instructions: Provide participants with clear instructions on how to collect the stool sample, including dietary restrictions and hygiene recommendations. Ensure that they understand the process.

3. Collection: Participants use disposable scoops or spatulas to collect a representative portion of their stool sample, avoiding contact with urine or toilet water. They collect approximately 5-10 g of stool, as required for the study, and transfer the collected stool into the labeled collection container, ensuring a tight seal.

4. Storage and Transport: Immediately place the collection container in a biohazard bag to prevent leakage or contamination. Place the biohazard bag containing the sample into a ziplock bag or a secondary container to further prevent leakage. Keep the samples on ice packs or in a cooler during transportation to the laboratory to maintain a cold chain, preserving microbial DNA.

5. Documentation: Record the date and time of sample collection, along with relevant participant information. Maintain a detailed record of the samples collected, including unique identifiers for each sample.

6. Storage at the Laboratory: Upon arrival at the laboratory, store the stool samples at -80℃ or as per the lab's specific storage protocols until DNA extraction.

7. Data Management: Create a database to track sample information, storage location, and associated metadata.

8. Sample Processing: Follow the lab's specific protocols for DNA extraction and metagenomic analysis.

9. Reporting: Record all findings and data generated during metagenomics analysis for future reference and publication.

10. Quality Control: Implement quality control measures throughout the process to ensure the integrity of the samples and data.

**Standard Operating Procedure for Processing Tissue Specimen for Histological Assessment**

**Objective:** The purpose of this Standard Operating Procedure (SOP) is to establish guidelines and protocols for the processing and verification of tissue specimens. This SOP outlines the steps to ensure consistent and accurate handling of tissue samples, from collection to analysis.

**Materials and Equipment:** To ensure safe and efficient lung tissue samples collection, the following equipment is required: tissue collection containers (e.g., cryovials, formalin containers)**,** personal protective equipment (PPE) including gloves, lab coats, and safety goggles, tissue processing equipment (e.g., microtomes, cryostats), reagents for tissue preservation and analysis, microscopes and imaging systems, data recording and analysis software.

**Procedure:**

1. Tissue Sectioning, Preparation, and Histochemical Staining: Slice the frozen lung tissue specimens to a thickness of 5 μm using the Leica CM1100 cryostat machine.

2. Slide Preparation: Sections for Immunohistochemistry (IHC) are fixed in -20℃ acetone for 5 minutes. Sections for *In Situ* Hybridization (ISH) are fixed in formalin for 1 minute, followed by three dips in Saline-Sodium Citrate (SSC) buffer. All sections, after drying on the bench, are immediately refrozen at -80℃.

3. Rehydration and Staining: After removing the cryosections from -80℃, sequentially rehydrate them in 100%, 95%, and 70% ethanol solutions.

4. Hematoxylin-Eosin (H&E) Staining: Wash rehydrated slides with tap water. Immerse slides in hematoxylin dye for 1 minute. Dip slides 10 times in tap water. Apply eosin for 30 seconds. Dip slides 10 times in tap water. Immerse consecutively in 96% ethanol, acetone, and xylene. Apply mounting medium and seal slides with nail polish.

5. Quality Control Regularly calibrate and maintain laboratory equipment to ensure accurate results. Implement quality control measures to monitor the accuracy and reliability of analyses.

**Standard Operating Procedure (SOP) for Exosome Isolation from Primary Lung Tumor and Plasma Samples via Ultracentrifugation**

**Objective:** To isolate exosomes from primary lung tumor tissue and blood samples from the same patients using ultracentrifugation.

**Materials and Reagents:** Primary lung tumor tissue samples, Blood samples from the same patients, Phosphate-buffered saline (PBS), Sterile centrifuge tubes (15 mL and 50 mL), Centrifuge with a swinging bucket rotor, Ultracentrifuge with appropriate rotors, Ultracentrifuge tubes (e.g., Beckman Coulter Polyallomer Tubes), Differential centrifugation buffer (0.22 µm filtered PBS), Ultracentrifuge tube liners, Sucrose or iodixanol gradient solutions, Ultracentrifuge tubes for gradients, Ultrafiltration units (e.g., Amicon Ultra-15 Centrifugal Filters), Sterile pipettes and tips, Sterile syringes and needles, Disposable gloves, and Ice.

**Procedure:**

1. Collection and Preparation of Samples. Collect primary lung tumor tissue samples and blood samples from the same patients following ethical guidelines. Centrifuge blood samples at 1,500 x g for 15 minutes at 4°C to separate plasma.

2. Exosome Isolation from Tumor Tissue. Homogenize the tumor tissue samples in PBS. Centrifuge the homogenate at 300 x g for 10 minutes to remove cell debris. Transfer the supernatant to a new tube.

3. Exosome Isolation from Plasma. Centrifuge the plasma at 2,000 x g for 10 minutes to remove residual cells. Transfer the supernatant to a new tube and centrifuge it at 10,000 x g for 30 minutes to remove larger debris. Collect the supernatant, which contains smaller vesicles.

4. Ultracentrifugation. Transfer the supernatants from steps 2 and 3 into ultracentrifuge tubes. Balance the tubes and ultracentrifuge at 100,000 x g for 2 hours at 4°C.

5. Exosome Pellet. Carefully remove the supernatant. Resuspend the exosome pellet in a small volume of PBS.

6. Gradient Ultracentrifugation (Optional). Prepare a sucrose or iodixanol gradient in ultracentrifuge tubes. Layer the exosome suspension on top of the gradient. Ultracentrifuge at 100,000 x g for 16-18 hours. Collect exosomes from the gradient interface.

7. Exosome Concentration. If necessary, concentrate the exosomes using an ultrafiltration unit. Wash with PBS if needed.

8. Exosome Storage: Store the isolated exosomes at -20°C for future analysis.

**Notes.** All steps should be performed at 4°C or on ice to maintain exosome integrity. Centrifuge steps should be carried out using appropriate centrifuge rotors and adapters for 15 mL and 50 mL tubes. Verify the quality and quantity of isolated exosomes using appropriate characterization methods (e.g., nanoparticle tracking analysis, electron microscopy, Western blotting, or RNA analysis). Always follow biohazard and safety protocols when handling patient samples.
