# Supplementary material for: LUNGBANK: a novel biorepository strategy tailored for comprehensive multiomics analysis and P-medicine applications in lung cancer
Source: Turk J Biol. 2024 May 28;48(3):203–17. doi: 10.55730/1300-0152.2696 (PMC11265891; doi:10.55730/1300-0152.2696)
Supplement: Supplementary file 2 [file Supplementary_Data-2-Demography.docx]

**Quantitative Data Analysis**

**Table 1.** Histological classifications and Tumor stage/grade categorizations

| **Rank** | **Patient ID** | **Histology** | **TNM** | **Stage** | **Grade** |
| --- | --- | --- | --- | --- | --- |
| 1 | 1 | SCC | T3N0M0 | IIB | 2 |
| 2 | 2 | LUAD | T4N1M0 | IIIA | 3 |
| 3 | 3 | SCC | T2aN1M0 | IIA | 3 |
| 4 | 4 | SCC | T2aN1M0 | IIA | 2 |
| 5 | 5 | SCC | T2bN1M0 | IIB | 3 |
| 6 | 6 | SCC | T2bN0M0 | IIA | 1 |
| 7 | 7 | SCC | T3N1M0 | IIIA | 3 |
| 8 | 8 | SCC | T3N0M0 | IIB | 2 |
| 9 | 10 | SCC | T2aN0M0 | IB | 2 |
| 10 | 11 | LUAD | T4N1M0 | IIIA | 3 |
| 11 | 12 | LUAD | T2bN0M0 | IIA | 2 |
| 12 | 13 | LUAD | T1bN0M0 | IA | 3 |
| 13 | 14 | LUAD | T3N0M0 | IIB | 3 |
| 14 | 16 | SCC | T4N0M0 | IIIA | 3 |
| 15 | 17 | SCC | T3N0M0 | IIB | 2 |
| 16 | 18 | SCC | T2aN0M0 | IB | 2 |
| 17 | 19 | LUAD | T2bN0M0 | IIA | 2 |
| 18 | 20 | LCLC | T1bN0M0 | IA | 3 |
| 19 | 21 | LCLC | T4N0M0 | IIIA | 3 |
| 20 | 22 | SCC | T3N2M0 | IIIA | 3 |
| 21 | 23 | SCC | T2aN1M0 | IIA | 2 |
| 22 | 24 | LUAD | T4N2M0 | IIIB | 3 |
| 23 | 25 | SCC | T3N1M0 | IIIA | 2 |
| 24 | 26 | LUAD | T1bN0M0 | IA | 2 |
| 25 | 27 | LUAD | T2aN0M0 | IB | 3 |
| 26 | 28 | SCC | T2bN1M0 | IIB | 2 |
| 27 | 29 | SCC | T2aN0M0 | IB | 2 |
| 28 | 30 | SCC | T4N0M0 | IIIA | 1 |
| 29 | 31 | SCC | T3N0M0 | IIB | 2 |
| 30 | 32 | LUAD | T2aN0M0 | IB | 3 |
| 31 | 33 | SCC | T3N1M0 | IIIA | 2 |
| 32 | 34 | LCLC | T3N0M0 | IIB | 3 |
| 33 | 35 | LUAD | T3N0M0 | IIB | 3 |
| 34 | 36 | SCC | T2aN0M0 | IB | 1 |
| 35 | 37 | SCC | T2aN1M0 | IIA | 2 |
| 36 | 38 | SCC | T4N1M0 | IIIA | 2 |
| 37 | 39 | SCC | T2bN1M0 | IIB | 2 |
| 38 | 40 | SCC | T3N1M0 | IIIA | 2 |
| 39 | 41 | LUAD | T2bN0M0 | IIA | 3 |
| 40 | 42 | SCC | T2bN2M0 | IIIA | 2 |
| 41 | 43 | SCC | T2aN0M0 | IB | 2 |
| 42 | 44 | LUAD | T1bN0M0 | IA | 3 |
| 43 | 46 | SCC | T2bN0M0 | IIA | 3 |
| 44 | 47 | LUAD | T2aN0M0 | IB | 3 |
| 45 | 48 | SCC | T2bN0M0 | IIA | 2 |
| 46 | 49 | LUAD | T2aN0M0 | IB | 3 |
| 47 | 51 | SCC | T2bN1M0 | IIB | 2 |
| 48 | 52 | SCC | T3N0M0 | IIB | 3 |
| 49 | 54 | SCC | T4N1M0 | IIIA | 2 |
| 50 | 55 | LUAD | T3N0M0 | IIB | 3 |
| 51 | 56 | SCC | T3N0M0 | IIB | 2 |
| 52 | 57 | SCC | T3N0M0 | IIB | 2 |
| 53 | 58 | LCLC | T3N1MO | IIIA | 3 |
| 54 | 59 | SCC | T4N0M0 | IIIA | 2 |
| 55 | 60 | CARCINOID | T2aN0M0 | IB | 2 |
| 56 | 61 | LUAD | T4N0M0 | IIIA | 3 |
| 57 | 62 | SCC | T3N0M0 | IIB | 3 |
| 58 | 63 | SCC | T3N1M0 | IIIA | 3 |
| 59 | 64 | SCC | T4N0M0 | IIIA | 3 |
| 60 | 65 | SCC | T1aN0M0 | IA | 2 |
| 61 | 66 | SCC | T3N0M0 | IIB | 3 |
| 62 | 67 | LUAD | T3N0M0 | IIB | 3 |
| 63 | 68 | SCC | T3N0M0 | IIB | 2 |
| 64 | 69 | LUAD | T2aN0M0 | IB | 2 |
| 65 | 70 | LUAD | T3N0M0 | IIB | 3 |
| 66 | 71 | SCC | T2bN1M0 | IIB | 2 |
| 67 | 72 | SCC | T2aN0M0 | IB | 3 |
| 68 | 73 | SCC | T3N0M0 | IIB | 3 |
| 69 | 74 | SCC | T3N0M0 | IIB | 3 |
| 70 | 75 | SCC | T3N1M0 | IIIA | 2 |
| 71 | 76 | SCC | T2bN1M0 | IIB | 1 |
| 72 | 77 | SCC | T3N0M0 | IIB | 3 |
| 73 | 78 | LCLC | T3N0M0 | IIB | 3 |
| 74 | 79 | SCC | T3N0M0 | IIB | 3 |
| 75 | 80 | SCC | T3N0M0 | IIB | 3 |
| 76 | 81 | LUAD | T3N0M0 | IIB | 3 |
| 77 | 82 | SCC | T3N0M0 | IIB | 2 |
| 78 | 83 | SCC | T3N0M0 | IIB | 3 |
| 79 | 85 | SCC | T3N0M0 | IIB | 3 |
| 80 | 86 | SCC | T2bN0M0 | IIA | 3 |
| 81 | 87 | SCC | T4N1M0 | IIIA | 3 |
| 82 | 88 | LUAD | T2bN2M0 | IIIA | 3 |
| 83 | 88 | SCC | T2bN0M0 | IIA | 1 |
| 84 | 89 | LUAD | T3N0M0 | IIB | 3 |
| 85 | 90 | LUAD | T3N1M0 | IIIA | 3 |
| 86 | 91 | SCC | T3N1M0 | IIIA | 2 |
| 87 | 92 | SCC | T3N0M0 | IIB | 3 |
| 88 | 93 | LUAD | T3N0M0 | IIB | 3 |
| 89 | 94 | SCC | T3N1M0 | IIIA | 3 |
| 90 | 95 | LUAD | T2bN2M0 | IIIA | 3 |
| 91 | 96 | LUAD | T2aN0M0 | IB | 2 |
| 92 | 97 | SCC | T2aN0M0 | IB | 2 |
| 93 | 98 | SCC | T4N1M0 | IIIA | 2 |
| 94 | 99 | LUAD | T3N0M0 | IIB | 3 |
| 95 | 99 | SCC | T2bN0M0 | IIA | 1 |
| 96 | 100 | SCC | T2aN0M0 | IB | 3 |
| 97 | 101 | SCC | T3N0M0 | IIB | 2 |
| 98 | 102 | LUAD | T3N2M0 | IIIA | 3 |
| 99 | 103 | LUAD | T2aN0M0 | IB | 3 |
| 100 | 104 | SCLC | T2bN1M1 | IVB | 4 |
| 101 | 105 | LUAD | T3N1M0 | IIIA | 3 |
| 102 | 106 | SCC | T4N1M0 | IIIA | 3 |
| 103 | 107 | SCC | T3N1M0 | IIIA | 3 |
| 104 | 108 | SCC | T4N0M0 | IIIA | 3 |
| 105 | 109 | SCC | T3N1M0 | IIIA | 2 |
| 106 | 110 | SCC | T3N1M0 | IIIA | 3 |
| 107 | 111 | LUAD | T2bN0M0 | IIA | 3 |
| 108 | 112 | SCC | T2bN0M0 | IIA | 2 |
| 109 | 113 | SCC | T3N0M0 | IIB | 2 |
| 110 | 114 | SCC | T3N1M0 | IIIA | 3 |
| 111 | 115 | SCC | T3N1M0 | IIIA | 3 |
| 112 | 116 | SCC | T3N0M0 | IIB | 2 |
| 113 | 117 | SCC | T3N0M0 | IIB | 2 |
| 114 | 118 | SCC | T4N0M0 | IIIA | 2 |
| 115 | 119 | SCC | T2bN0M0 | IIA | 3 |
| 116 | 120 | LUAD | T2bN0M0 | IIA | 2 |
| 117 | 121 | LCLC | T2aN1M0 | IIA | 3 |
| 118 | 122 | LUAD | T1bN0M0 | IA | 1 |
| 119 | 123 | LUAD | T4N0M0 | IIIA | 3 |
| 120 | 124 | SCC | T4N0M0 | IIIA | 3 |
| 121 | 125 | LUAD | T3N1M0 | IIIA | 3 |
| 122 | 126 | SCC | T3N1M0 | IIIA | 2 |
| 123 | 127 | LUAD | T2aN2M0 | IIIA | 3 |
| 124 | 128 | SCC | T4N2M0 | IIIB | 2 |
| 125 | 129 | LUAD | T2bN0M0 | IIA | 3 |
| 126 | 130 | SCC | T3N1M0 | IIIA | 2 |
| 127 | 131 | LUAD | T2bN1M0 | IIB | 3 |
| 128 | 132 | SCC | T2bN0M0 | IIA | 1 |
| 129 | 133 | LUAD | T2bN1M0 | IIB | 2 |
| 130 | 134 | SCC | T3N0M0 | IIB | 3 |
| 131 | 135 | LCLC | T2bN1M0 | IIB | 3 |
| 132 | 136 | SCC | T3N0M0 | IIB | 1 |
| 133 | 137 | ASQ | T3N0M0 | IIB | 3 |
| 134 | 138 | ASQ | T3N0M1 | IVB | 3 |
| 135 | 139 | LUAD | T3N0M0 | IIB | 3 |
| 136 | 140 | SCC | T3N2M0 | IIIA | 2 |
| 137 | 141 | SCC | T4N1M0 | IIIA | 2 |
| 138 | 142 | SCC | T2bN2M0 | IIIA | 3 |
| 139 | 143 | SCC | T3N0M0 | IIB | 2 |
| 140 | 144 | SCLC | T3N0M0 | IIB | 4 |
| 141 | 145 | SCC | T2bN0M0 | IIA | 3 |
| 142 | 146 | SCC | T4N1M0 | IIIA | 2 |
| 143 | 147 | LCLC | T4N2M0 | IIIB | 3 |
| 144 | 148 | SCC | T2aN0M0 | IB | 3 |
| 145 | 149 | SCC | T4N1M0 | IIIA | 2 |
| 146 | 150 | SCC | T3N1M0 | IIIA | 2 |
| 147 | 151 | SCC | T2bN1M0 | IIB | 3 |
| 148 | 152 | SCC | T3N0M0 | IIB | 2 |
| 149 | 153 | SCC | T1bN0M0 | IA | 2 |
| 150 | 154 | SCC | T3N1M0 | IIIA | 2 |
| 151 | 155 | LUAD | T2bN0M0 | IIA | 3 |
| 152 | 156 | SCC | T2bN0M0 | IIA | 3 |
| 153 | 159 | LUAD | T4N0M0 | IIIA | 3 |
| 154 | 160 | LUAD | T2aN1M0 | IIA | 2 |
| 155 | 161 | SCC | T2bN1M0 | IIB | 3 |
| 156 | 162 | SCC | T4N1M0 | IIIA | 1 |
| 157 | 163 | SCC | T4N1M0 | IIIA | 2 |
| 158 | 164 | SCC | T4N1M0 | IIIA | 2 |
| 159 | 165 | SCC | T3N0M0 | IIB | 2 |
| 160 | 166 | SCC | T4N1M0 | IIIA | 3 |
| 161 | 168 | LCLC | T2aN0M0 | IB | 3 |
| 162 | 169 | SCC | T1bN0M0 | IA | 3 |
| 163 | 170 | SCC | T1bN0M0 | IA | 2 |
| 164 | 171 | SCLC | T4N1M0 | IIIA | 4 |
| 165 | 172 | LUAD | T3N0M0 | IIB | 3 |
| 166 | 173 | SCC | T3N0M0 | IIB | 3 |
| 167 | 174 | LUAD | T2bN0M0 | IIA | 2 |
| 168 | 175 | SCC | T4N1M0 | IIIA | 3 |
| 169 | 177 | SCC | T4N0M0 | IIIA | 2 |
| 170 | 178 | LUAD | T3N0M0 | IIB | 1 |
| 171 | 179 | LUAD | T2aN1M0 | IIA | 2 |
| 172 | 180 | LUAD | T1bN1M0 | IIA | 2 |
| 173 | 181 | LUAD | T1bN1M0 | IIA | 1 |
| 174 | 182 | LUAD | T1bN1M0 | IIA | 3 |
| 175 | 183 | SCC | T1bN0M0 | IA | 2 |
| 176 | 184 | SCC | T4N1M0 | IIIA | 2 |
| 177 | 185 | SCC | T4N2M0 | IIIB | 2 |
| 178 | 186 | SCC | T4N0M0 | IIIA | 2 |
| 179 | 187 | SCC | T3N0M0 | IIB | 2 |
| 180 | 188 | SCC | T2aN0M0 | IB | 1 |
| 181 | 235 | LUAD | T2aN0M0 | IB | 3 |
| 182 | 236 | SARKOMATOİD | T2bN2M0 | IIIA | 3 |
| 183 | 237 | SCC | T2aN1M0 | IIA | 3 |
| 184 | 239 | SCC | T2aN1M0 | IIA | 1 |
| 185 | 241 | SCC | T3N0M0 | IIB | 2 |
| 186 | 242 | LUAD | T3N0M0 | IIB | 2 |
| 187 | 243 | SCC | T2aN0M0 | IB | 2 |
| 188 | 244 | SCC | T3N0M0 | IIB | 3 |
| 189 | 245 | SCC | T3N0M0 | IIB | 3 |
| 190 | 246 | SCC | T3N0M0 | IIB | 2 |
| 191 | 247 | SCC | T2bN1M0 | IIB | 2 |
| 192 | 248 | LUAD | T3N1M0 | IIIA | 3 |
| 193 | 249 | SCC | T1bN0M0 | IA | 2 |
| 194 | 250 | SCC | T4N0M0 | IIIA | 2 |
| 195 | 251 | NEUROENDOCRINE | T2aN0M0 | IB | 3 |
| 196 | 252 | LUAD | T2bN0M0 | IIA | 2 |
| 197 | 253 | SCC | T3N1M0 | IIIA | 2 |
| 198 | 254 | SCC | T4N0M0 | IIIA | 3 |
| 199 | 255 | SCC | T1aN0M0 | IA | 3 |
| 200 | 256 | SCC | T3N1M0 | IIIA | 1 |
| 201 | 257 | SCC | T1bN0M0 | IA | 3 |
| 202 | 258 | SCC | T2aN0M0 | IB | 3 |
| 203 | 259 | SCC | T2aN0M0 | IB | 3 |
| 204 | 260 | SCC | T2bN2M0 | IIIA | 2 |
| 205 | 261 | LUAD | T1bN0M0 | IA | 1 |
| 206 | 262 | LUAD | T1BN1M0 | IIA | 3 |
| 207 | 263 | SCLC | T1aN0M0 | IA | 4 |
| 208 | 264 | LUAD | T2bN2M0 | IIIA | 3 |
| 209 | 265 | LUAD | T2bN2M1 | IVB | 3 |
| 210 | 266 | SCC | T3N0M0 | IIB | 3 |
| 211 | 267 | LUAD | T2bN0M0 | IIA | 2 |
| 212 | 268 | CARCINOID | T2bN0M0 | IIA | 1 |
| 213 | 269 | LUAD | T3N0M0 | IIB | 3 |
| 214 | 270 | SCC | T2bN1M0 | IIB | 1 |
| 215 | 271 | LUAD | T1bN0M0 | IA | 2 |
| 216 | 272 | LUAD | T2bN2M0 | IIIA | 3 |
| 217 | 273 | SCC | T1bN0M0 | IA | 1 |
| 218 | 274 | LUAD | T1bN0M0 | IA | 2 |
| 219 | 275 | LUAD | T1aN0M0 | IA | 1 |
| 220 | 276 | SCC | T2bN0M0 | IIA | 2 |
| 221 | 277 | SCC | T1bN0M0 | IA | 2 |
| 222 | 278 | LUAD | T2bN1M0 | IIB | 3 |
| 223 | 279 | LUAD | T3N1M0 | IIIA | 3 |
| 224 | 280 | LUAD | T3N1M0 | IIIA | 3 |
| 225 | 281 | LUAD | T2bN2M0 | IIIA | 3 |
| 226 | 282 | SCC | T2aN2M0 | IIIA | 2 |
| 227 | 283 | SCC | T2bN0M0 | IIA | 3 |
| 228 | 284 | LUAD | T3N1M0 | IIIA | 3 |
| 229 | 285 | LUAD | T2bN0M0 | IIA | 2 |
| 230 | 286 | SCC | T2bN2M0 | IIIA | 3 |
| 231 | 287 | LUAD | T2bN1M0 | IIB | 3 |
| 232 | 288 | LUAD | T2bN2M0 | IIIA | 3 |
| 233 | 289 | SCC | T2aN0M0 | IB | 2 |
| 234 | 290 | SCC | T2aN0M0 | IB | 2 |
| 235 | 291 | LUAD | T1bN2M0 | IIIA | 3 |
| 236 | 292 | LUAD | T2bN0M0 | IIA | 2 |
| 237 | 293 | SCC | T1bN0M0 | IA | 2 |

**Table 2.** Key epidemiological data

| **Rank** | **Patient ID** | **Age** | **Sex** | **Smoking history** |
| --- | --- | --- | --- | --- |
| 1 | 1 | 63 | M | S |
| 2 | 2 | 71 | M | S |
| 3 | 3 | 61 | M | S |
| 4 | 4 | 68 | M | S |
| 5 | 5 | 76 | M | S |
| 6 | 6 | 56 | M | S |
| 7 | 7 | 57 | M | S |
| 8 | 8 | 58 | M | S |
| 9 | 10 | 63 | F | S |
| 10 | 11 | 73 | M | S |
| 11 | 12 | 60 | M | S |
| 12 | 13 | 49 | M | S |
| 13 | 14 | 59 | F | S |
| 14 | 16 | 64 | M | S |
| 15 | 17 | 76 | M | S |
| 16 | 18 | 61 | M | S |
| 17 | 19 | 50 | M | S |
| 18 | 20 | 68 | M | S |
| 19 | 21 | 62 | M | NS |
| 20 | 22 | 61 | M | S |
| 21 | 23 | 57 | M | S |
| 22 | 24 | 50 | F | S |
| 23 | 25 | 62 | F | NS |
| 24 | 26 | 50 | M | S |
| 25 | 27 | 37 | M | S |
| 26 | 28 | 69 | M | S |
| 27 | 29 | 69 | M | S |
| 28 | 30 | 57 | M | S |
| 29 | 31 | 74 | M | S |
| 30 | 32 | 58 | M | S |
| 31 | 33 | 61 | M | S |
| 32 | 34 | 72 | M | S |
| 33 | 35 | 81 | M | S |
| 34 | 36 | 67 | F | S |
| 35 | 37 | 49 | M | S |
| 36 | 38 | 59 | M | S |
| 37 | 39 | 65 | M | S |
| 38 | 40 | 59 | M | S |
| 39 | 41 | 66 | M | S |
| 40 | 42 | 62 | M | S |
| 41 | 43 | 70 | M | S |
| 42 | 44 | 35 | M | NS |
| 43 | 46 | 65 | M | S |
| 44 | 47 | 47 | M | S |
| 45 | 48 | 65 | M | S |
| 46 | 49 | 72 | F | NS |
| 47 | 51 | 68 | M | NS |
| 48 | 52 | 66 | M | S |
| 49 | 54 | 73 | M | S |
| 50 | 55 | 61 | M | S |
| 51 | 56 | 69 | M | S |
| 52 | 57 | 73 | M | S |
| 53 | 58 | 62 | M | S |
| 54 | 59 | 63 | M | S |
| 55 | 60 | 55 | F | S |
| 56 | 61 | 52 | M | NS |
| 57 | 62 | 62 | M | S |
| 58 | 63 | 63 | M | S |
| 59 | 64 | 64 | M | S |
| 60 | 65 | 71 | M | S |
| 61 | 66 | 71 | M | S |
| 62 | 67 | 62 | M | S |
| 63 | 68 | 57 | M | S |
| 64 | 69 | 54 | M | S |
| 65 | 70 | 68 | M | S |
| 66 | 71 | 61 | M | S |
| 67 | 72 | 78 | M | S |
| 68 | 73 | 51 | M | S |
| 69 | 74 | 73 | M | S |
| 70 | 75 | 58 | F | S |
| 71 | 76 | 50 | M | S |
| 72 | 77 | 56 | M | S |
| 73 | 78 | 73 | M | S |
| 74 | 79 | 62 | M | S |
| 75 | 80 | 56 | M | S |
| 76 | 81 | 57 | F | S |
| 77 | 82 | 76 | M | S |
| 78 | 83 | 63 | M | S |
| 79 | 85 | 61 | F | S |
| 80 | 86 | 58 | M | S |
| 81 | 87 | 52 | M | S |
| 82 | 88 | 60 | M | S |
| 83 | 88 | 78 | M | S |
| 84 | 89 | 56 | M | S |
| 85 | 90 | 60 | M | S |
| 86 | 91 | 65 | M | S |
| 87 | 92 | 67 | M | S |
| 88 | 93 | 39 | M | S |
| 89 | 94 | 52 | M | NS |
| 90 | 95 | 62 | M | S |
| 91 | 96 | 46 | M | S |
| 92 | 97 | 58 | M | S |
| 93 | 98 | 59 | M | NS |
| 94 | 99 | 55 | M | NS |
| 95 | 99 | 71 | M | NS |
| 96 | 100 | 51 | M | NS |
| 97 | 101 | 65 | M | S |
| 98 | 102 | 52 | F | NS |
| 99 | 103 | 53 | M | S |
| 100 | 104 | 57 | M | S |
| 101 | 105 | 72 | F | NS |
| 102 | 106 | 65 | M | S |
| 103 | 107 | 68 | M | S |
| 104 | 108 | 59 | M | S |
| 105 | 109 | 62 | M | S |
| 106 | 110 | 77 | M | S |
| 107 | 111 | 46 | M | S |
| 108 | 112 | 57 | M | S |
| 109 | 113 | 64 | M | S |
| 110 | 114 | 67 | M | S |
| 111 | 115 | 60 | M | S |
| 112 | 116 | 54 | M | S |
| 113 | 117 | 70 | M | NS |
| 114 | 118 | 61 | M | NS |
| 115 | 119 | 65 | M | S |
| 116 | 120 | 69 | M | S |
| 117 | 121 | 70 | F | NS |
| 118 | 122 | 55 | F | S |
| 119 | 123 | 69 | M | S |
| 120 | 124 | 76 | M | S |
| 121 | 125 | 61 | M | S |
| 122 | 126 | 64 | M | S |
| 123 | 127 | 65 | M | S |
| 124 | 128 | 64 | M | S |
| 125 | 129 | 52 | M | NS |
| 126 | 130 | 46 | M | S |
| 127 | 131 | 62 | M | S |
| 128 | 132 | 55 | M | S |
| 129 | 133 | 85 | M | S |
| 130 | 134 | 60 | M | S |
| 131 | 135 | 56 | M | NS |
| 132 | 136 | 56 | M | S |
| 133 | 137 | 64 | M | S |
| 134 | 138 | 58 | M | S |
| 135 | 139 | 60 | M | S |
| 136 | 140 | 56 | M | S |
| 137 | 141 | 69 | M | S |
| 138 | 142 | 67 | M | S |
| 139 | 143 | 59 | M | NS |
| 140 | 144 | 68 | M | S |
| 141 | 145 | 49 | M | S |
| 142 | 146 | 74 | M | S |
| 143 | 147 | 63 | F | NS |
| 144 | 148 | 63 | M | S |
| 145 | 149 | 63 | M | S |
| 146 | 150 | 48 | M | NS |
| 147 | 151 | 62 | M | S |
| 148 | 152 | 63 | M | S |
| 149 | 153 | 58 | M | S |
| 150 | 154 | 53 | M | S |
| 151 | 155 | 73 | M | S |
| 152 | 156 | 56 | M | NS |
| 153 | 159 | 63 | M | S |
| 154 | 160 | 58 | M | S |
| 155 | 161 | 75 | M | S |
| 156 | 162 | 61 | M | S |
| 157 | 163 | 59 | M | S |
| 158 | 164 | 50 | M | S |
| 159 | 165 | 56 | M | S |
| 160 | 166 | 46 | M | S |
| 161 | 168 | 61 | M | S |
| 162 | 169 | 68 | M | S |
| 163 | 170 | 68 | M | S |
| 164 | 171 | 61 | M | S |
| 165 | 172 | 56 | M | S |
| 166 | 173 | 69 | M | S |
| 167 | 174 | 61 | M | S |
| 168 | 175 | 60 | M | S |
| 169 | 177 | 58 | F | S |
| 170 | 178 | 57 | M | S |
| 171 | 179 | 55 | M | S |
| 172 | 180 | 67 | M | S |
| 173 | 181 | 45 | F | S |
| 174 | 182 | 42 | M | NS |
| 175 | 183 | 52 | M | S |
| 176 | 184 | 70 | M | S |
| 177 | 185 | 60 | M | S |
| 178 | 186 | 68 | M | S |
| 179 | 187 | 67 | M | S |
| 180 | 188 | 41 | M | S |
| 181 | 235 | 59 | M | S |
| 182 | 236 | 50 | M | S |
| 183 | 237 | 73 | M | S |
| 184 | 239 | 60 | M | S |
| 185 | 241 | 60 | M | S |
| 186 | 242 | 68 | M | S |
| 187 | 243 | 75 | M | S |
| 188 | 244 | 64 | M | NS |
| 189 | 245 | 68 | M | S |
| 190 | 246 | 73 | M | S |
| 191 | 247 | 66 | M | NS |
| 192 | 248 | 68 | F | S |
| 193 | 249 | 66 | M | NS |
| 194 | 250 | 59 | M | S |
| 195 | 251 | 65 | M | S |
| 196 | 252 | 60 | M | S |
| 197 | 253 | 69 | M | S |
| 198 | 254 | 58 | M | NS |
| 199 | 255 | 51 | M | S |
| 200 | 256 | 65 | M | S |
| 201 | 257 | 51 | M | S |
| 202 | 258 | 74 | M | S |
| 203 | 259 | 55 | M | S |
| 204 | 260 | 45 | M | S |
| 205 | 261 | 57 | M | S |
| 206 | 262 | 48 | M | S |
| 207 | 263 | 54 | M | S |
| 208 | 264 | 68 | M | S |
| 209 | 265 | 62 | M | S |
| 210 | 266 | 58 | M | NS |
| 211 | 267 | 66 | M | S |
| 212 | 268 | 49 | F | S |
| 213 | 269 | 56 | M | S |
| 214 | 270 | 59 | M | S |
| 215 | 271 | 65 | M | S |
| 216 | 272 | 71 | M | S |
| 217 | 273 | 56 | M | S |
| 218 | 274 | 65 | M | S |
| 219 | 275 | 65 | M | NS |
| 220 | 276 | 68 | M | NS |
| 221 | 277 | 80 | M | NS |
| 222 | 278 | 50 | M | S |
| 223 | 279 | 51 | M | S |
| 224 | 280 | 65 | M | NS |
| 225 | 281 | 68 | M | NS |
| 226 | 282 | 68 | M | S |
| 227 | 283 | 64 | M | S |
| 228 | 284 | 60 | M | S |
| 229 | 285 | 71 | F | S |
| 230 | 286 | 61 | M | S |
| 231 | 287 | 63 | F | NS |
| 232 | 288 | 58 | F | S |
| 233 | 289 | 64 | M | NS |
| 234 | 290 | 74 | M | S |
| 235 | 291 | 57 | M | NS |
| 236 | 292 | 73 | M | S |
| 237 | 293 | 54 | M | NS |
