# Supplementary material for: LUNGBANK: a novel biorepository strategy tailored for comprehensive multiomics analysis and P-medicine applications in lung cancer
Source: Turk J Biol. 2024 May 28;48(3):203–17. doi: 10.55730/1300-0152.2696 (PMC11265891; doi:10.55730/1300-0152.2696)
Supplement: Supplementary file 3 [file Supplementary_Data-3-IHC.pdf]

**Patient ID: 1**

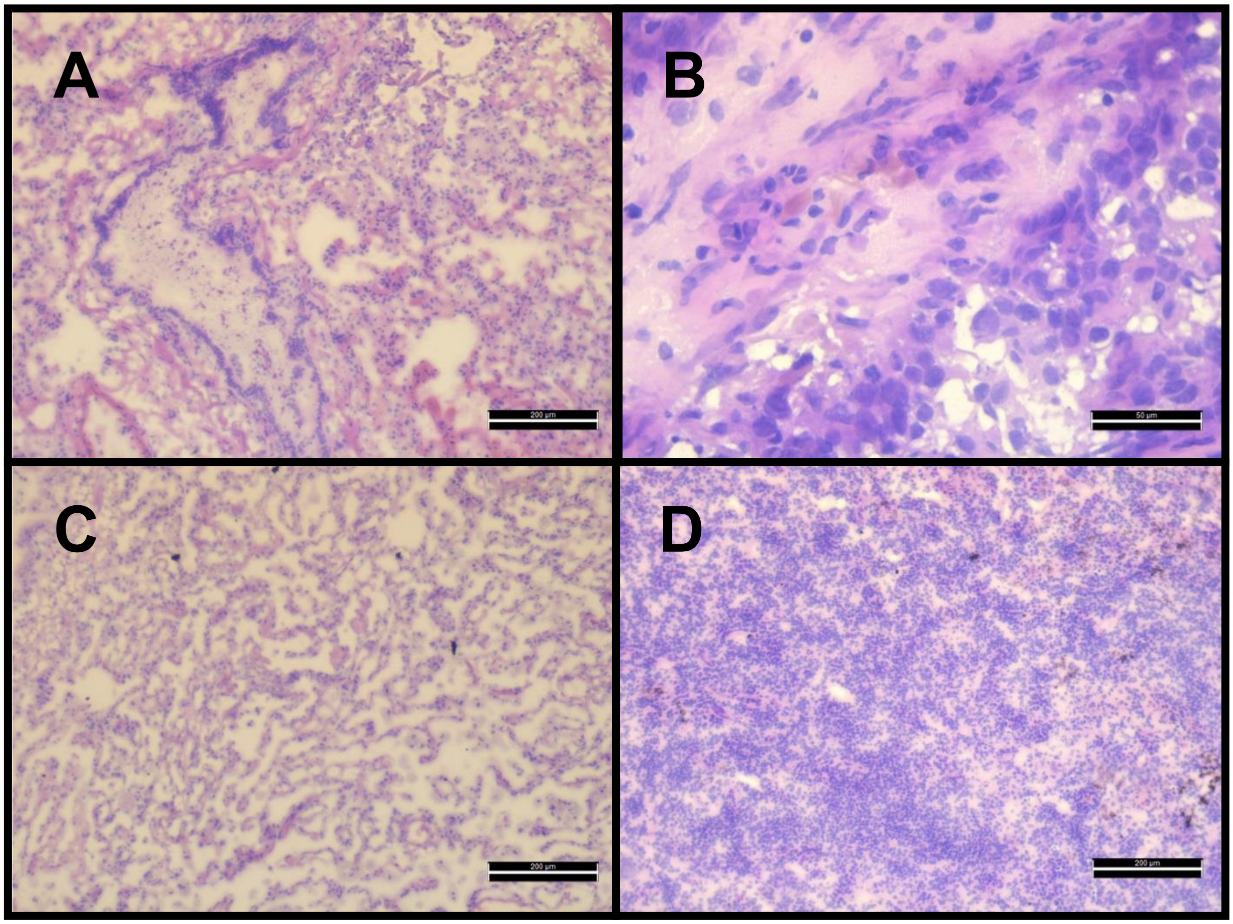


**A:** Normal lung, **B:** Squamous cell carcinoma, **C:** Reactive stroma (Tumor microenvironment, **D:** Lymph node

**Bar:** 200 μM

**Patient ID: 2**

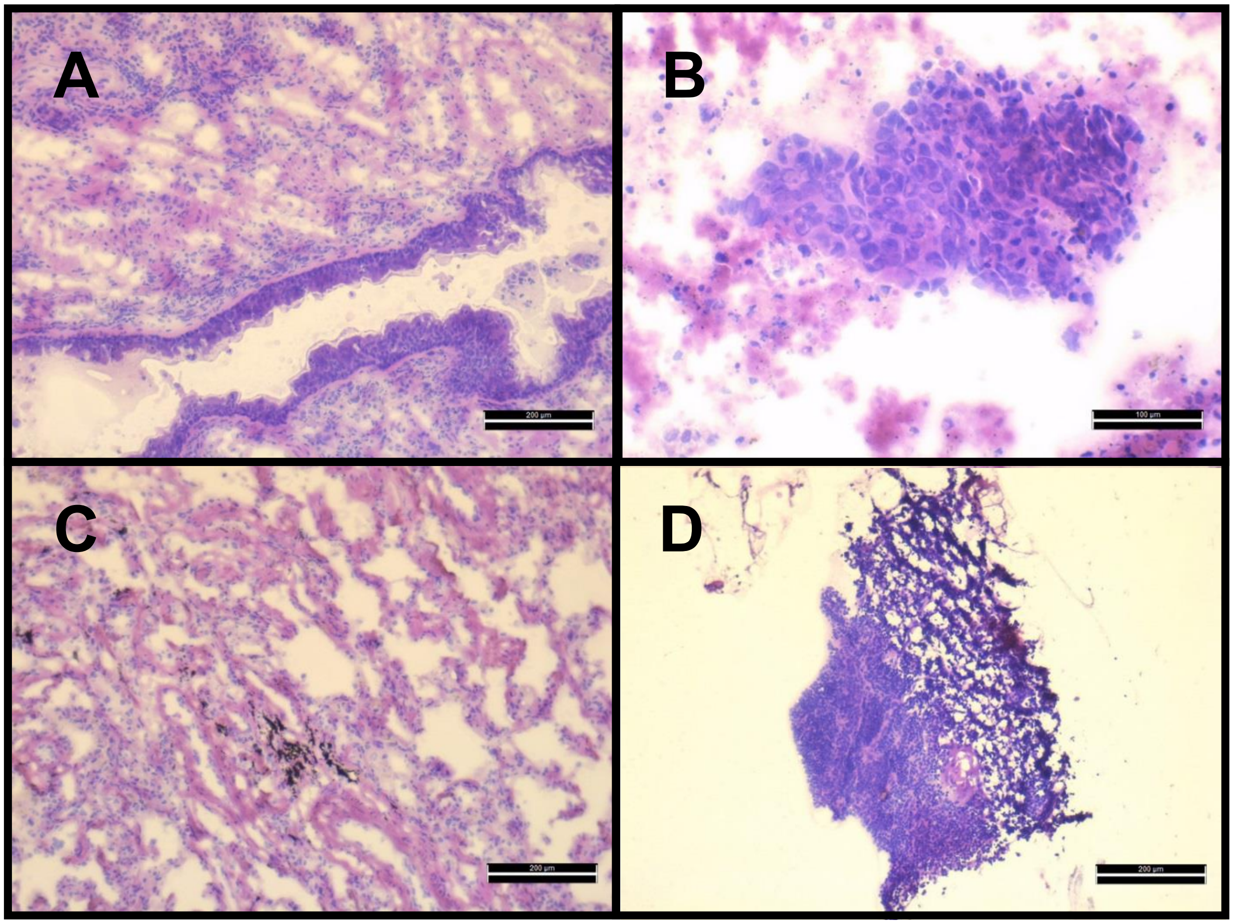


**A:** Normal lung, **B:** Lung adenocarcinoma, **C:** Reactive stroma (Tumor microenvironment, **D:** Lymph node

**Bar:** 200 µM

**Patient ID: 4**

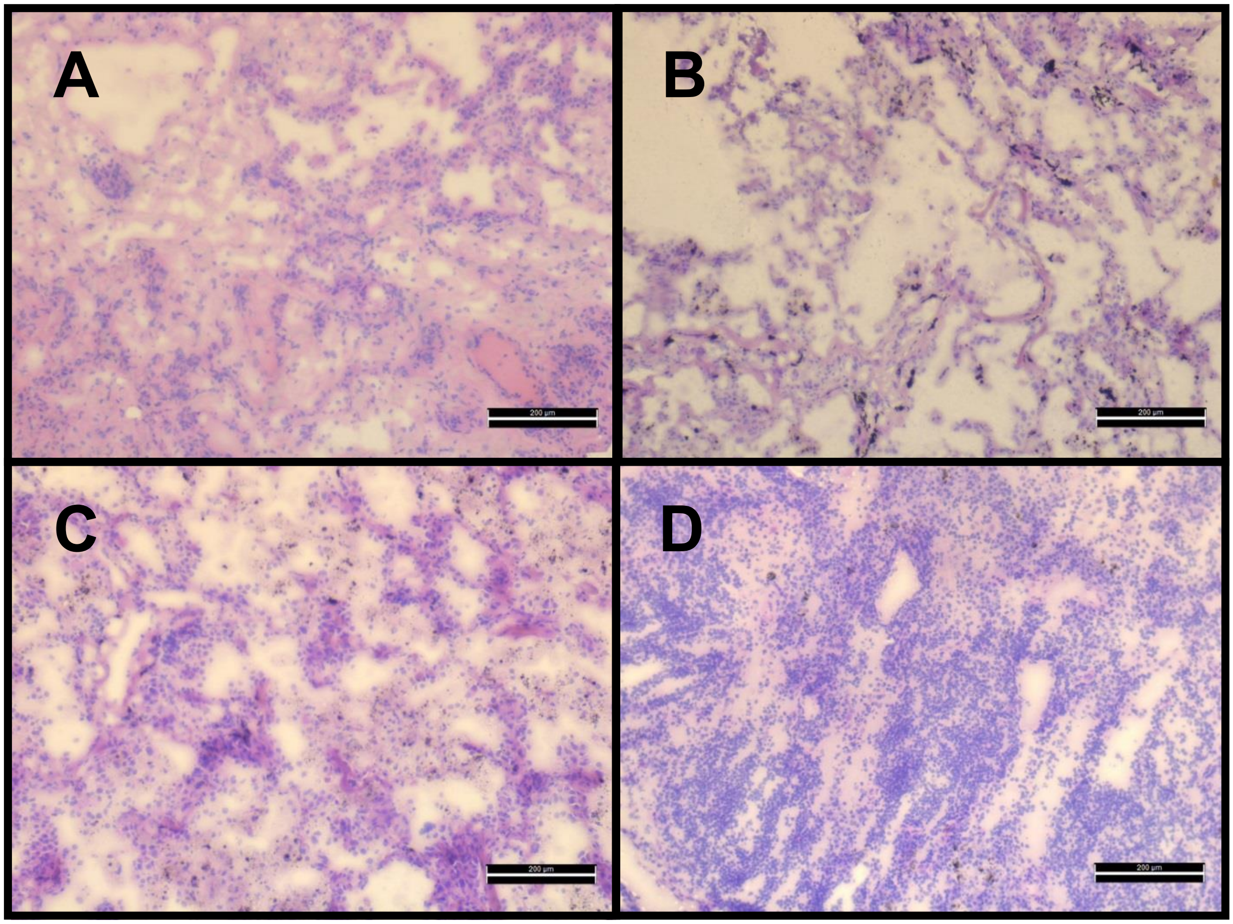


**A:** Normal lung, **B:** Squamous cell carcinoma, **C:** Reactive stroma (Tumor microenvironment, **D:** Lymph node

**Bar:** 200 μM

**Patient ID: 5**

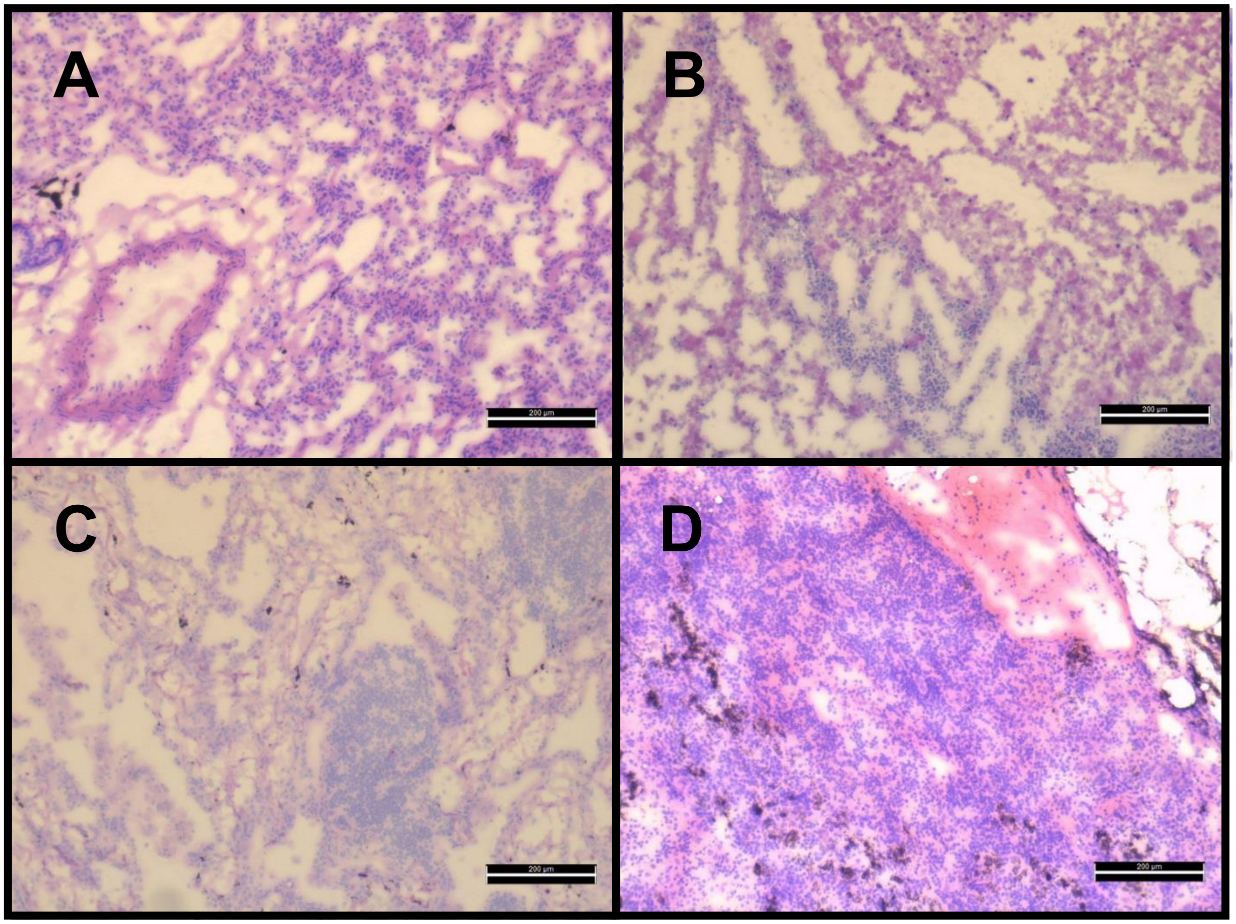


**A:** Normal lung, **B:** Squamous cell carcinoma, **C:** Reactive stroma (Tumor microenvironment, **D:** Lymph node

**Bar:** 200 μM

**Patient ID: 8**

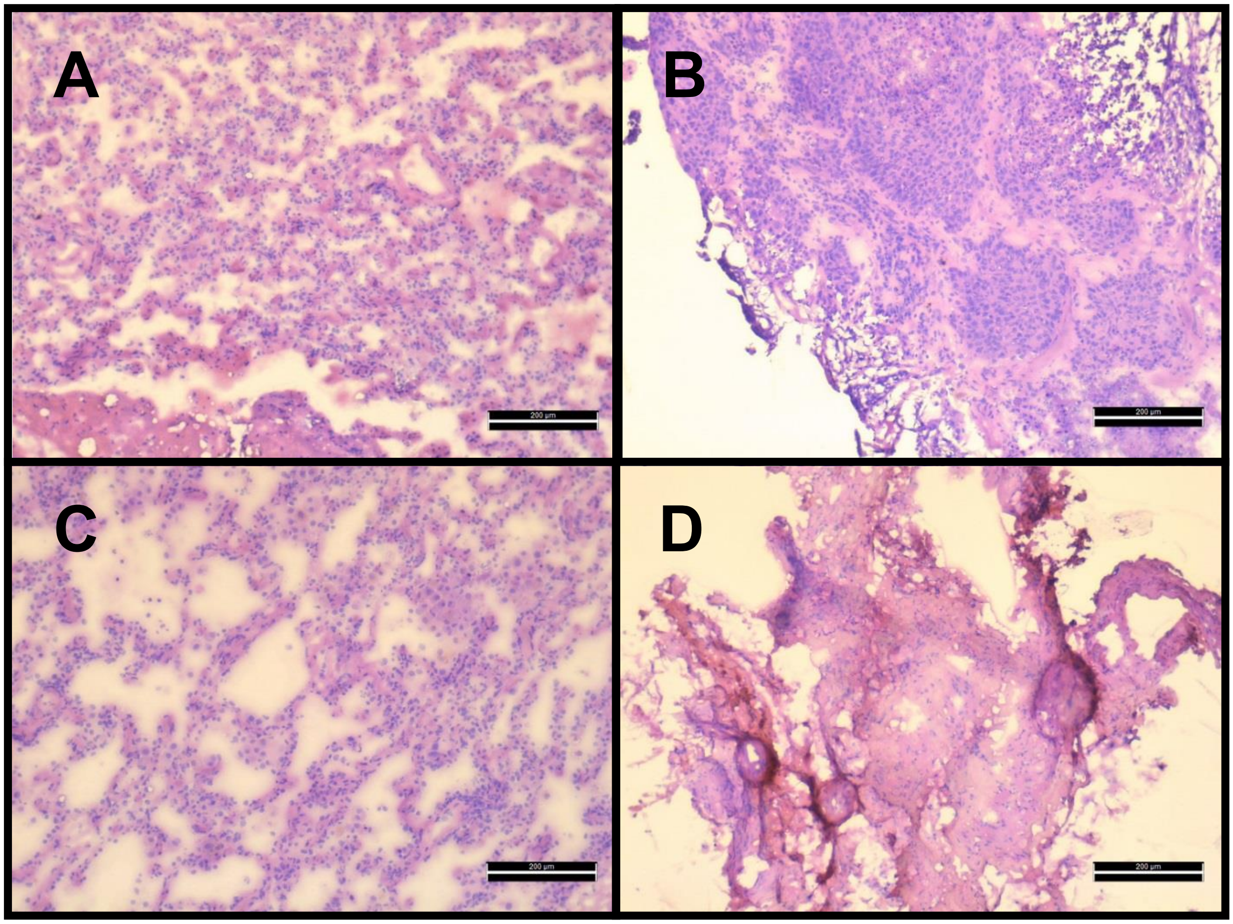


**A:** Normal lung, **B:** Squamous cell carcinoma, **C:** Reactive stroma (Tumor microenvironment, **D:** Lymph node

**Bar:** 200 μM

**Patient ID: 10**

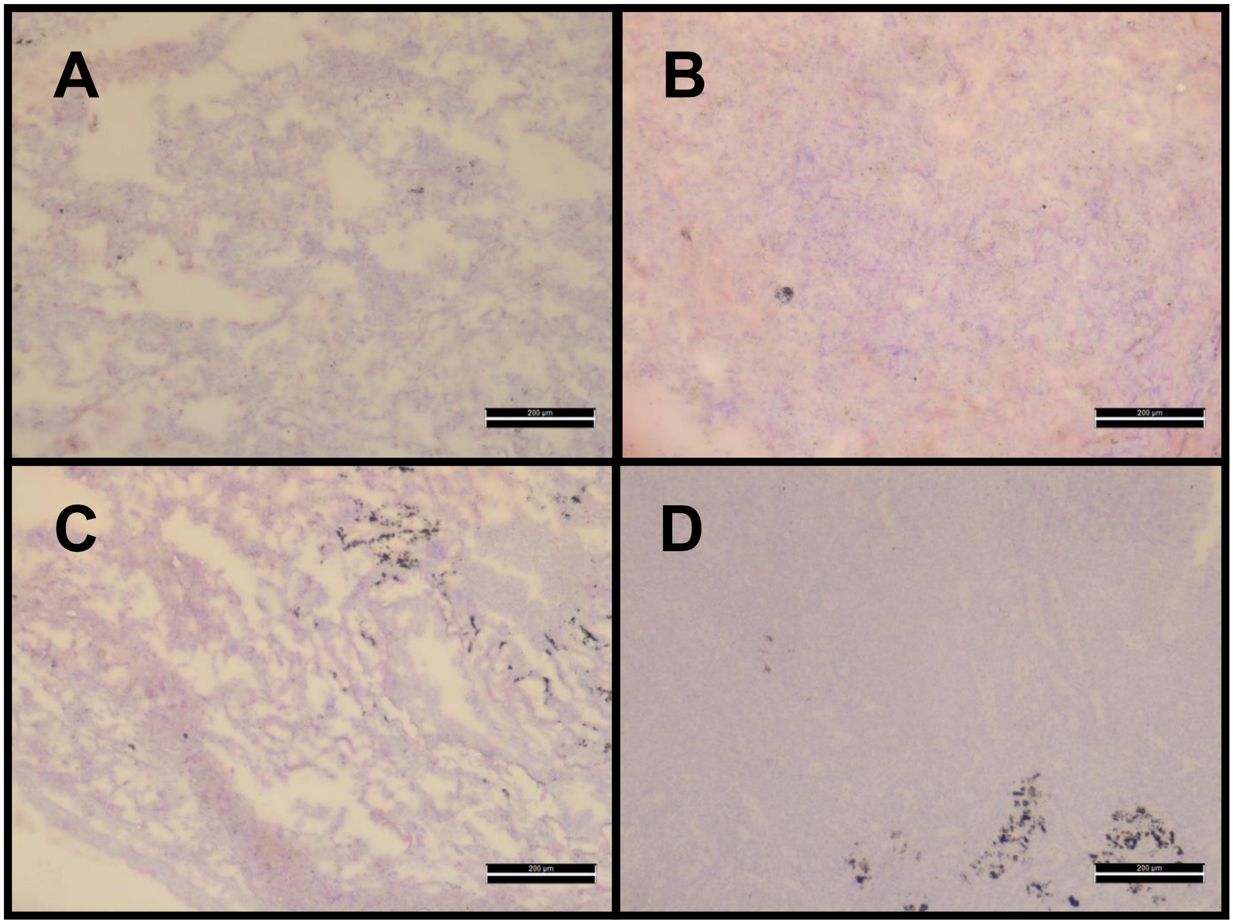


**A:** Normal lung, **B:** Squamous cell carcinoma, **C:** Reactive stroma (Tumor microenvironment, **D:** Lymph node

**Bar:** 200 μM

**Patient ID: 13**

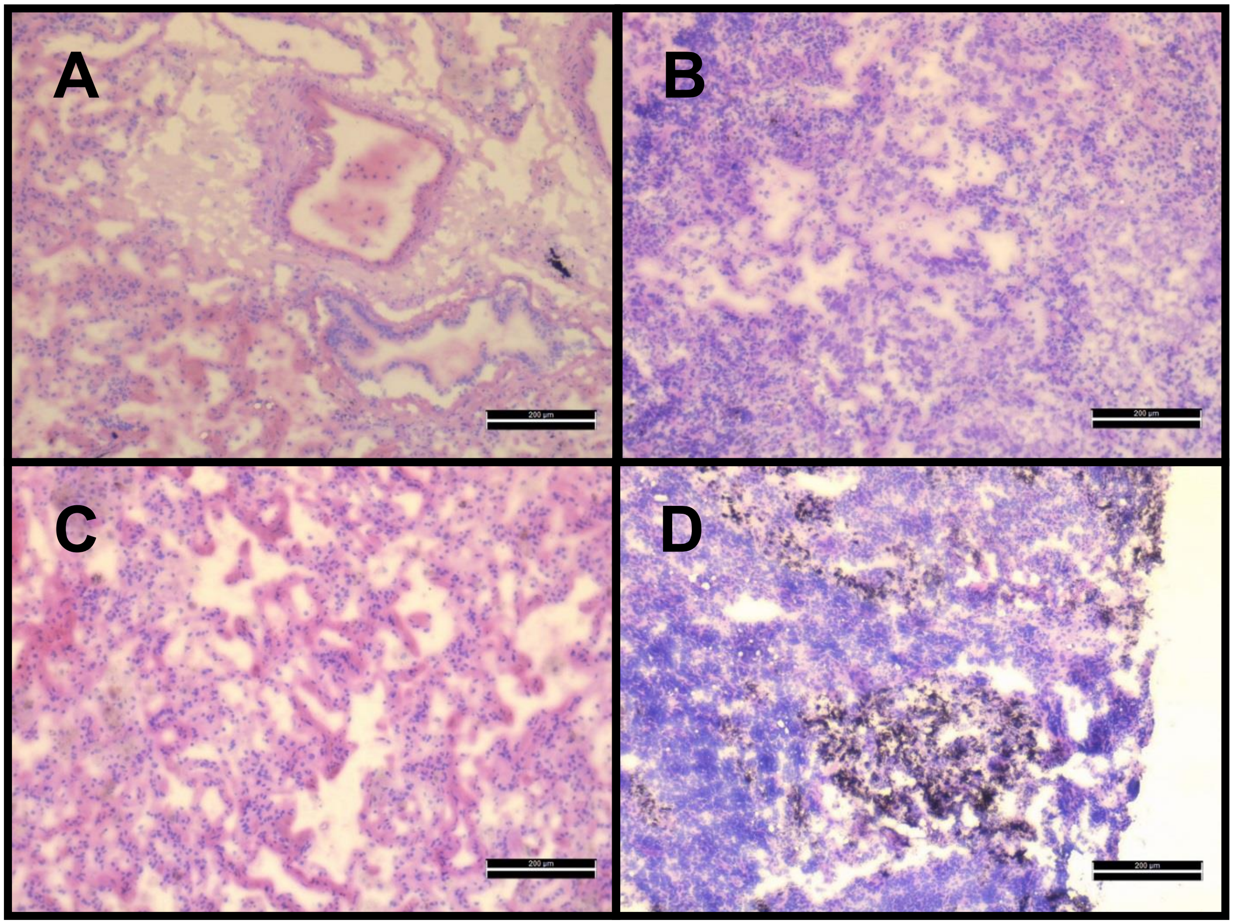


**A:** Normal lung, **B:** Lung adenocarcinoma, **C:** Reactive stroma (Tumor microenvironment, **D:** Lymph node

**Bar:** 200 μM

**Patient ID: 14**

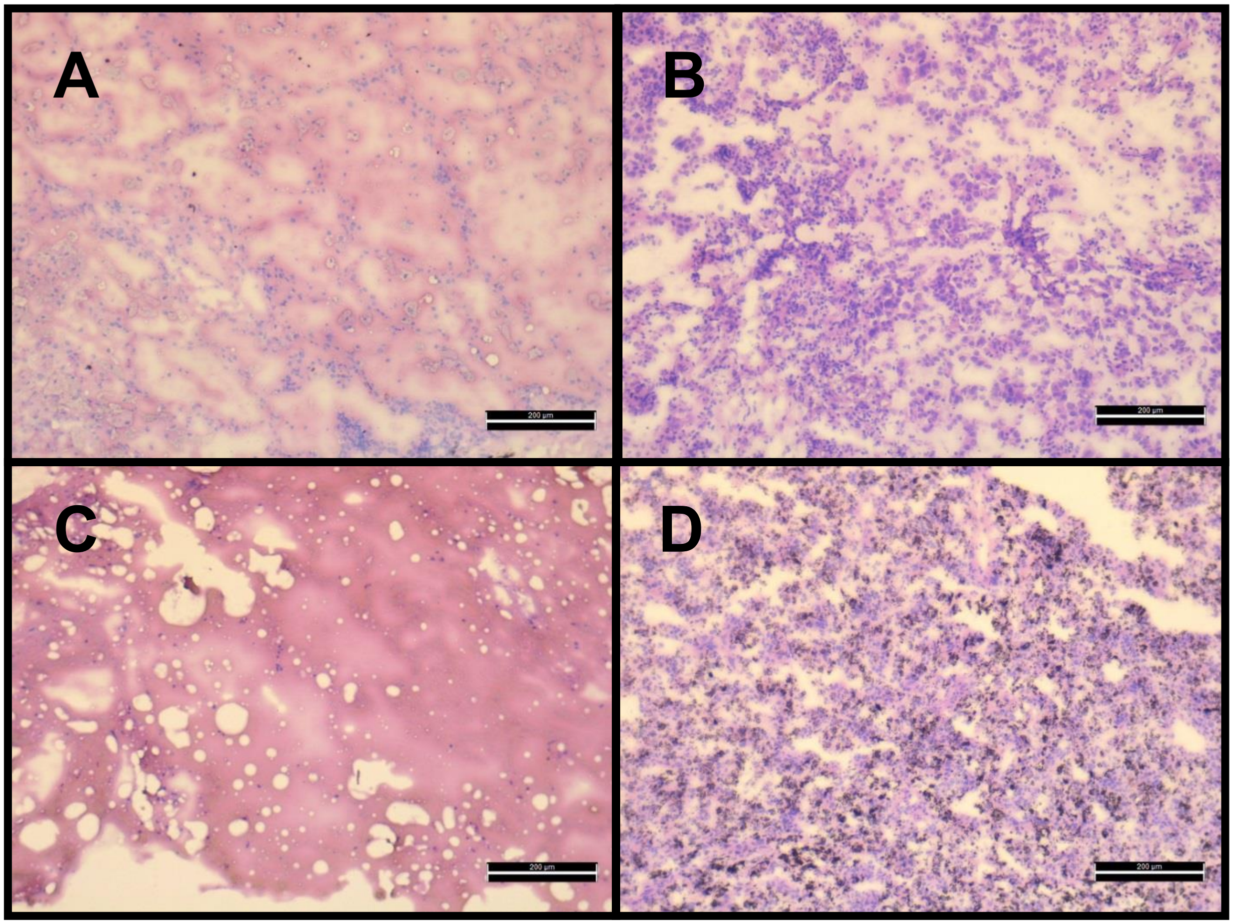


**A:** Normal lung, **B:** Lung adenocarcinoma, **C:** Reactive stroma (Tumor microenvironment, **D:** Lymph node

**Bar:** 200 μM

**Patient ID: 16**

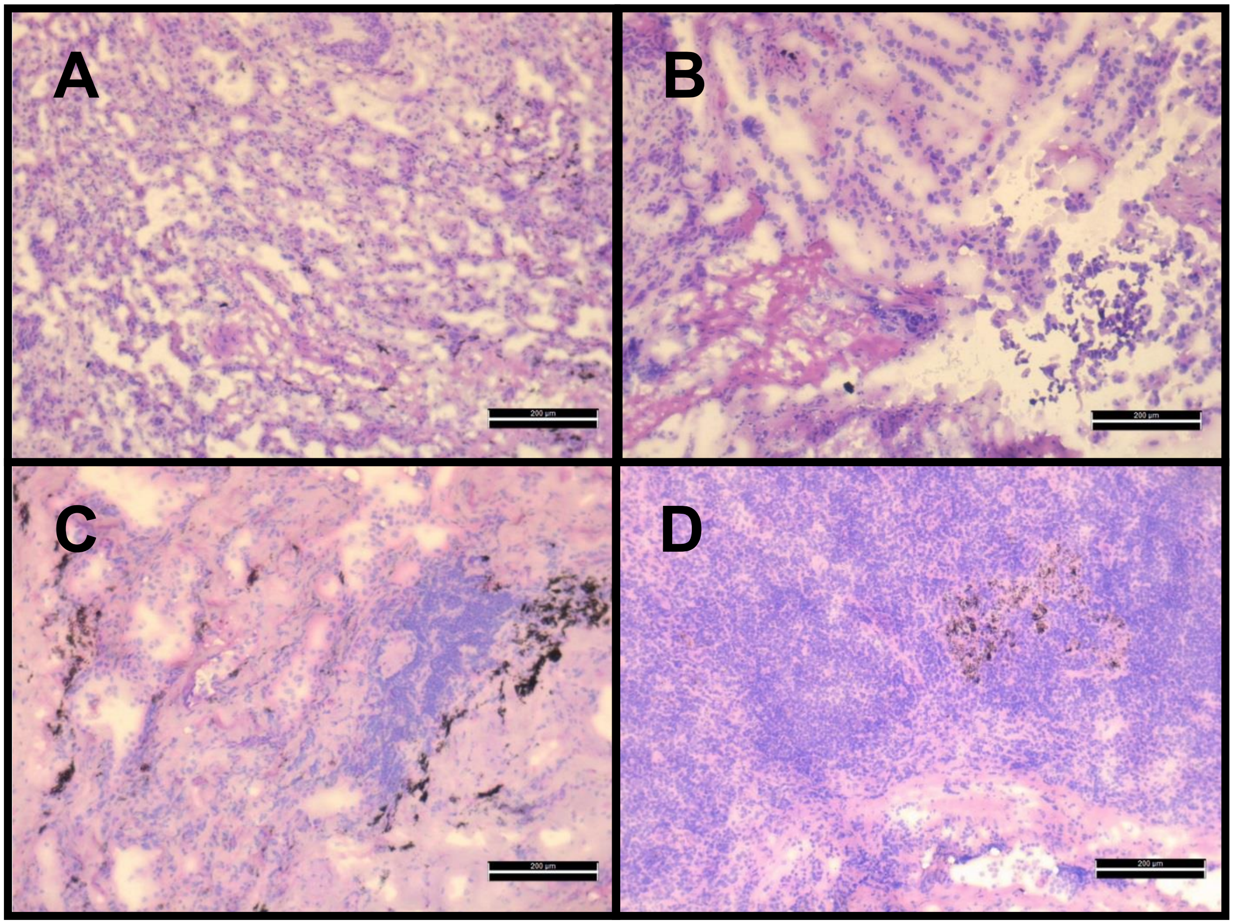


**A:** Normal lung, **B:** Squamous cell carcinoma, **C:** Reactive stroma (Tumor microenvironment, **D:** Lymph node

**Bar:** 200 μM

**Patient ID: 17**

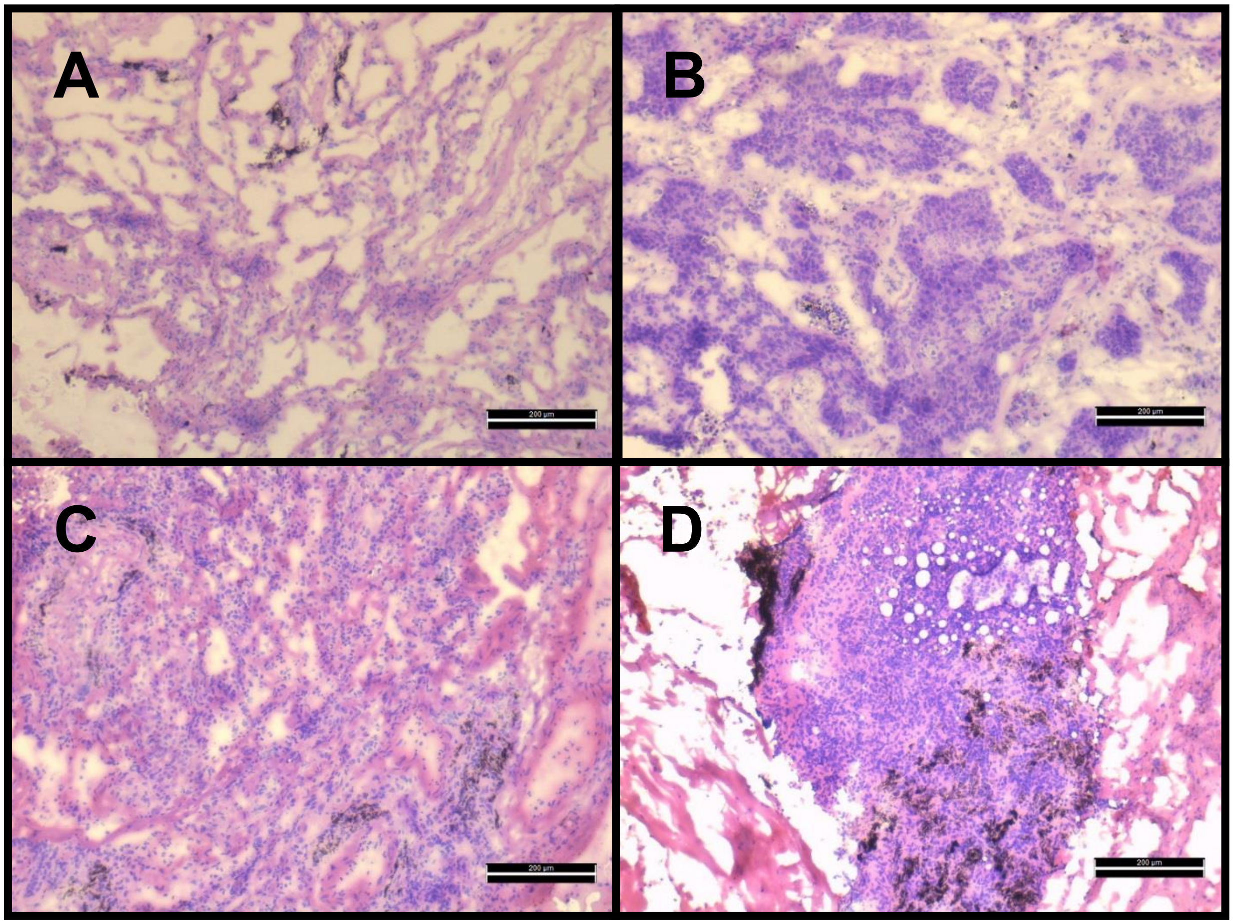


**A:** Normal lung, **B:** Squamous cell carcinoma, **C:** Reactive stroma (Tumor microenvironment, **D:** Lymph node

**Bar:** 200 μM

**Patient ID: 21**

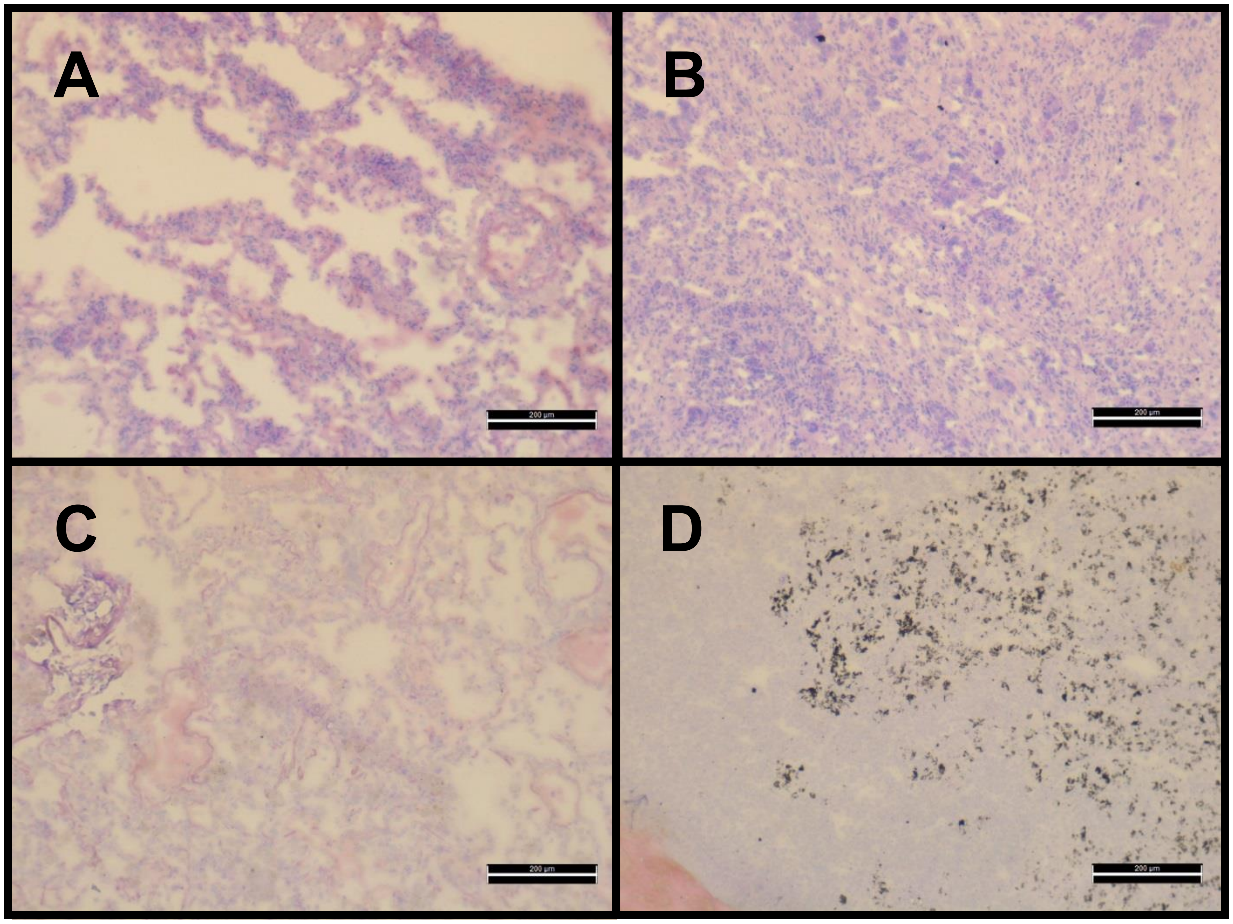


**A:** Normal lung, **B:** Large cell lung carcinoma, **C:** Reactive stroma (Tumor microenvironment, **D:** Lymph node

**Bar:** 200 µM

**Patient ID: 22**

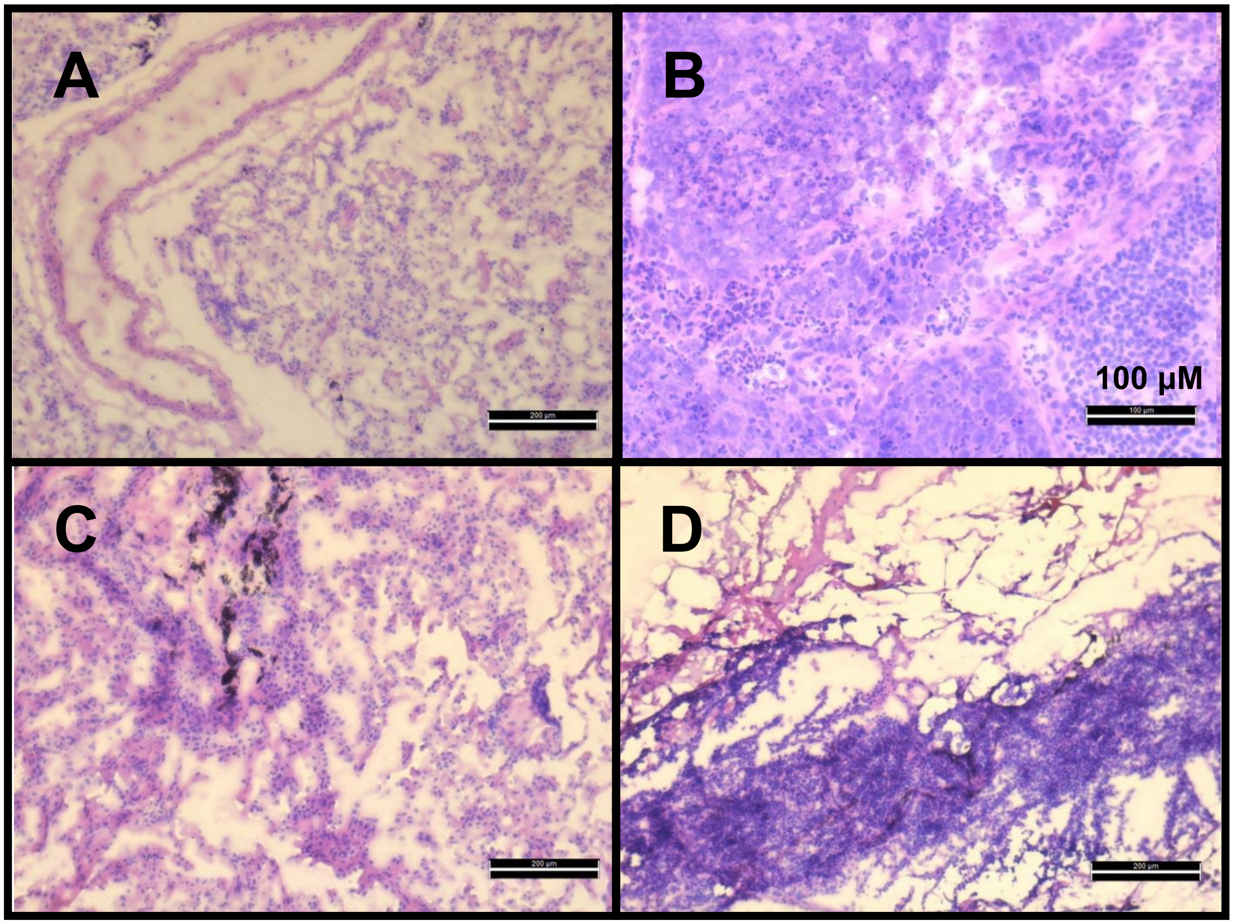


**A:** Normal lung, **B:** Squamous cell carcinoma, **C:** Reactive stroma (Tumor microenvironment, **D:** Lymph node

**Bar:** 200 µM

**Patient ID: 23**

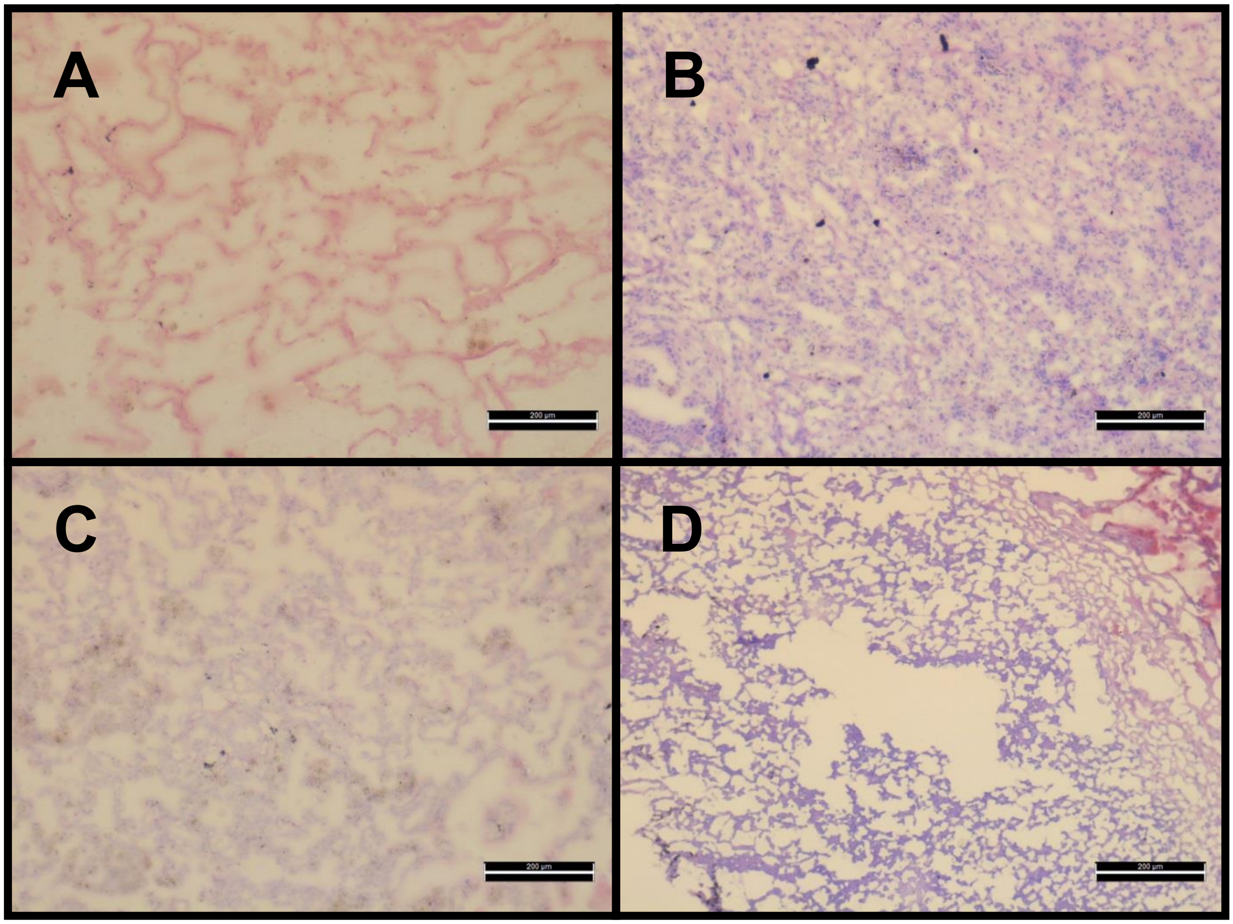


**A:** Normal lung, **B:** Squamous cell carcinoma, **C:** Reactive stroma (Tumor microenvironment, **D:** Lymph node

**Bar:** 200 µM

**Patient ID: 24**

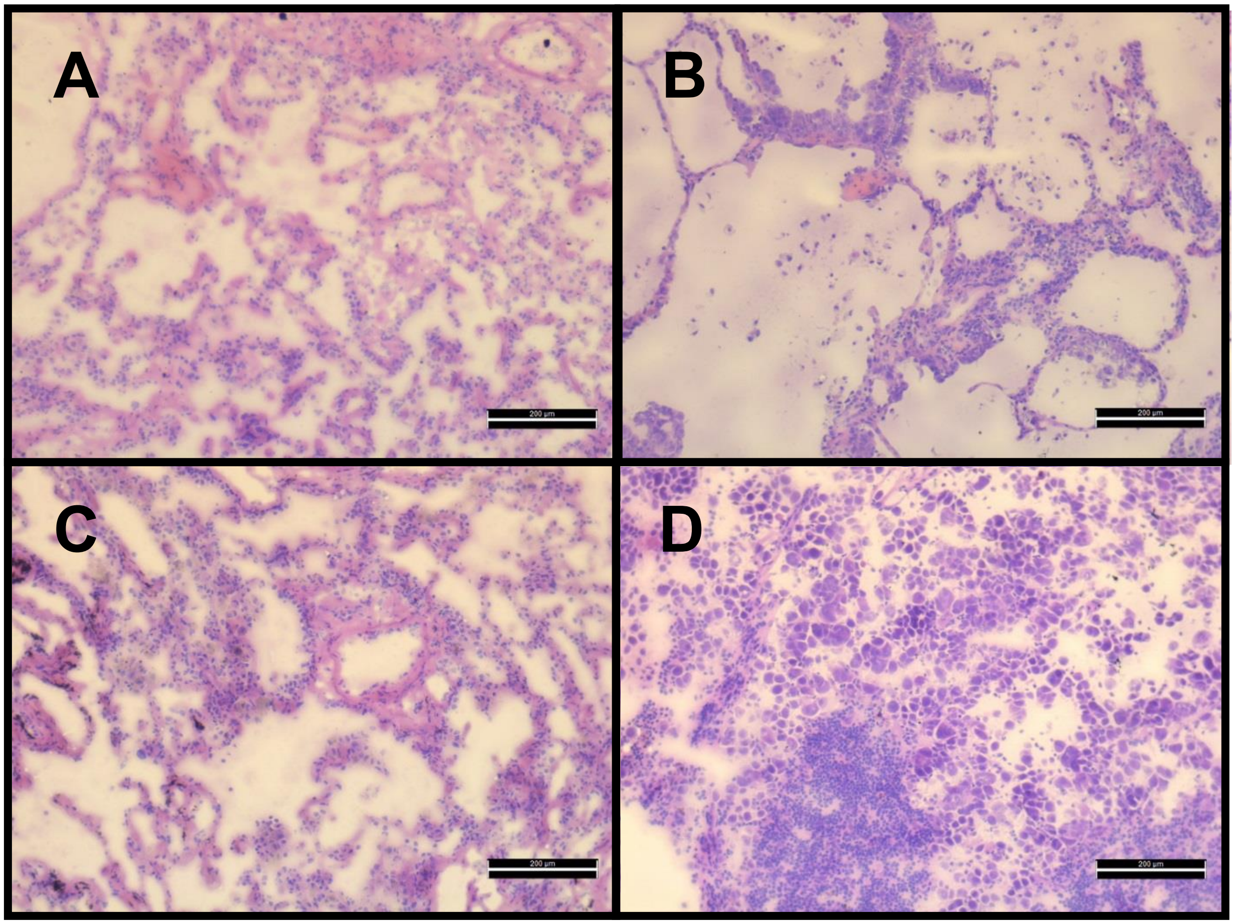


**A:** Normal lung, **B:** Lung adenocarcinoma, **C:** Reactive stroma (Tumor microenvironment, **D:** Lymph node

**Bar:** 200 µM

**Patient ID: 25**

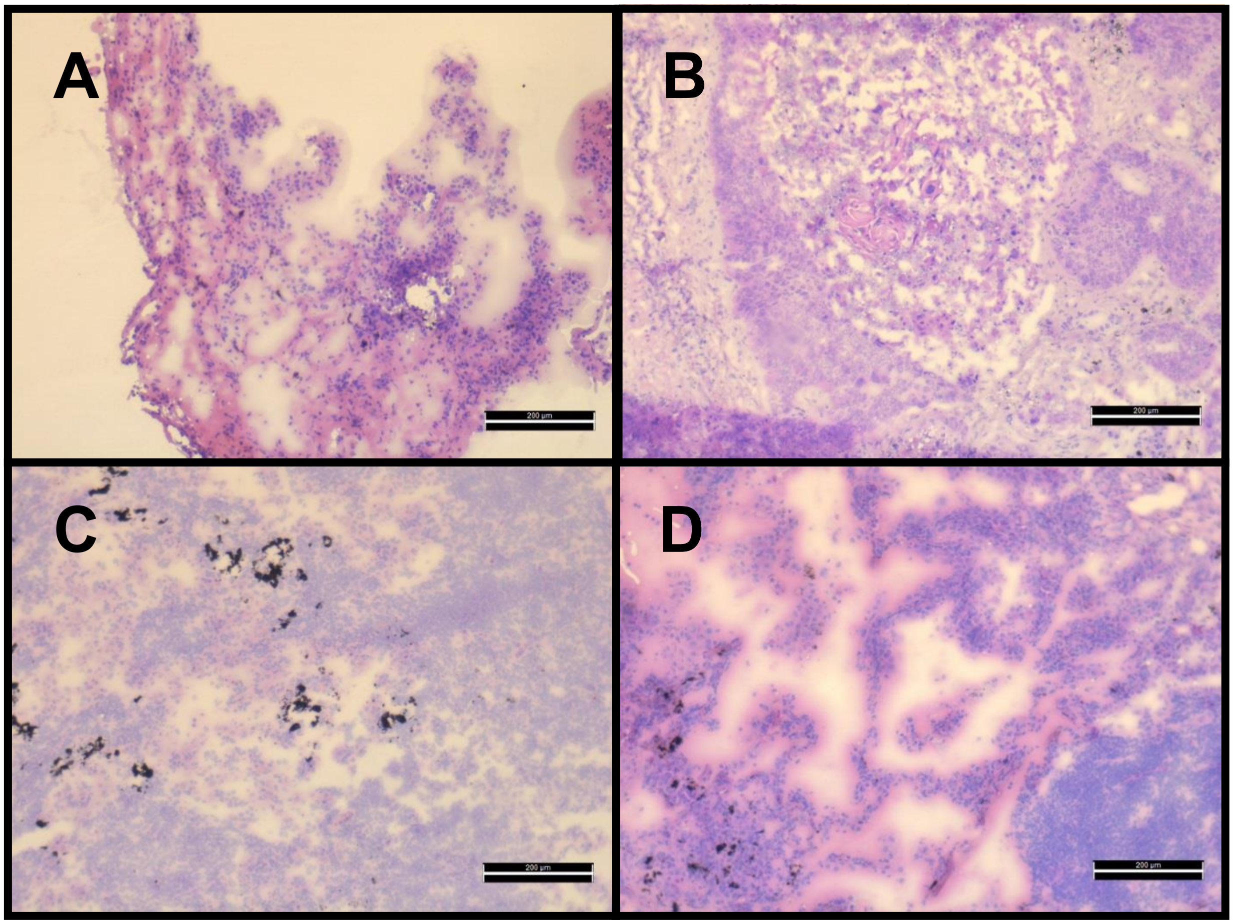


**A:** Normal lung, **B:** Squamous cell carcinoma, **C:** Reactive stroma (Tumor microenvironment, **D:** Lymph node

**Bar:** 200 μM

**Patient ID: 26**

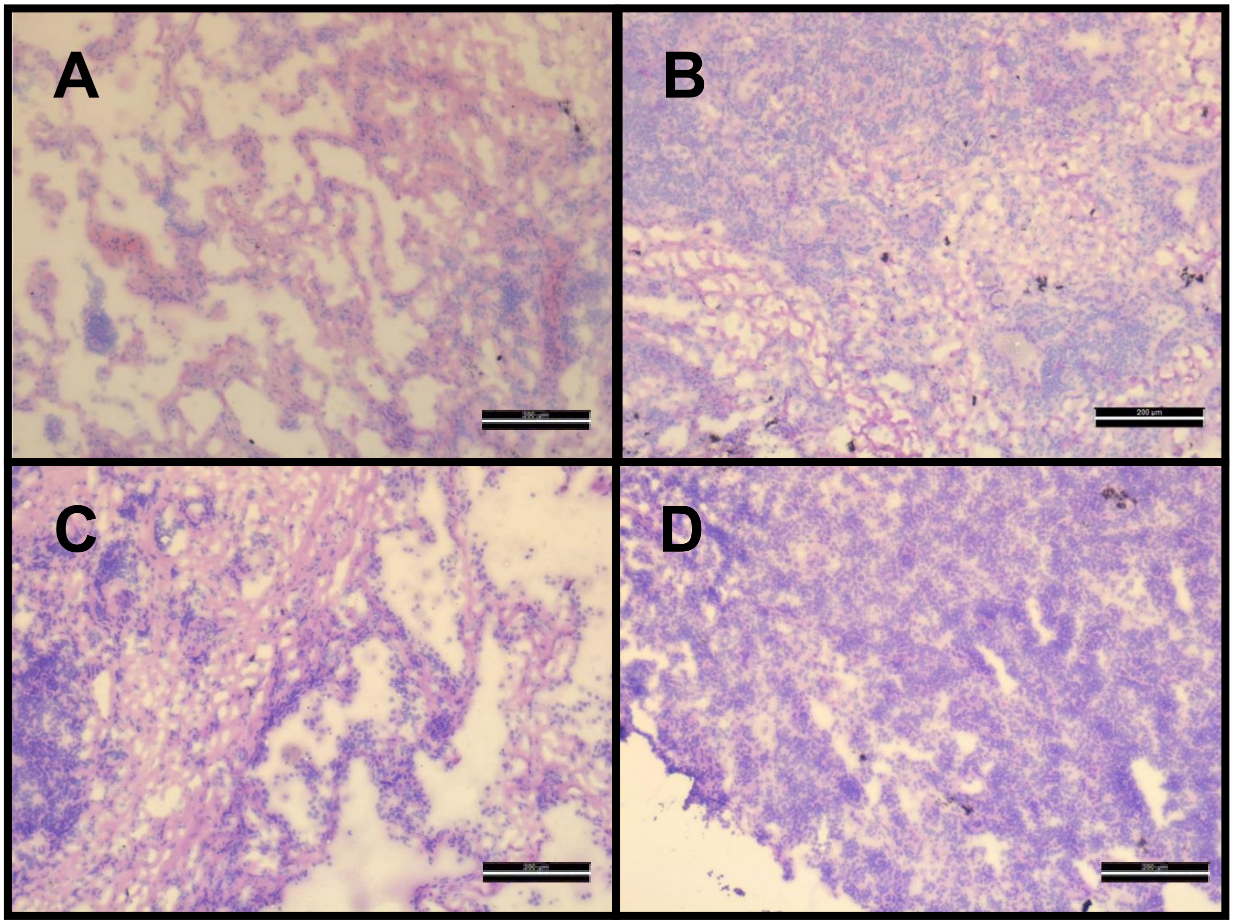


**A:** Normal lung, **B:** Lung adenocarcinoma, **C:** Reactive stroma (Tumor microenvironment, **D:** Lymph node

**Bar:** 200 μM

**Patient ID: 27**

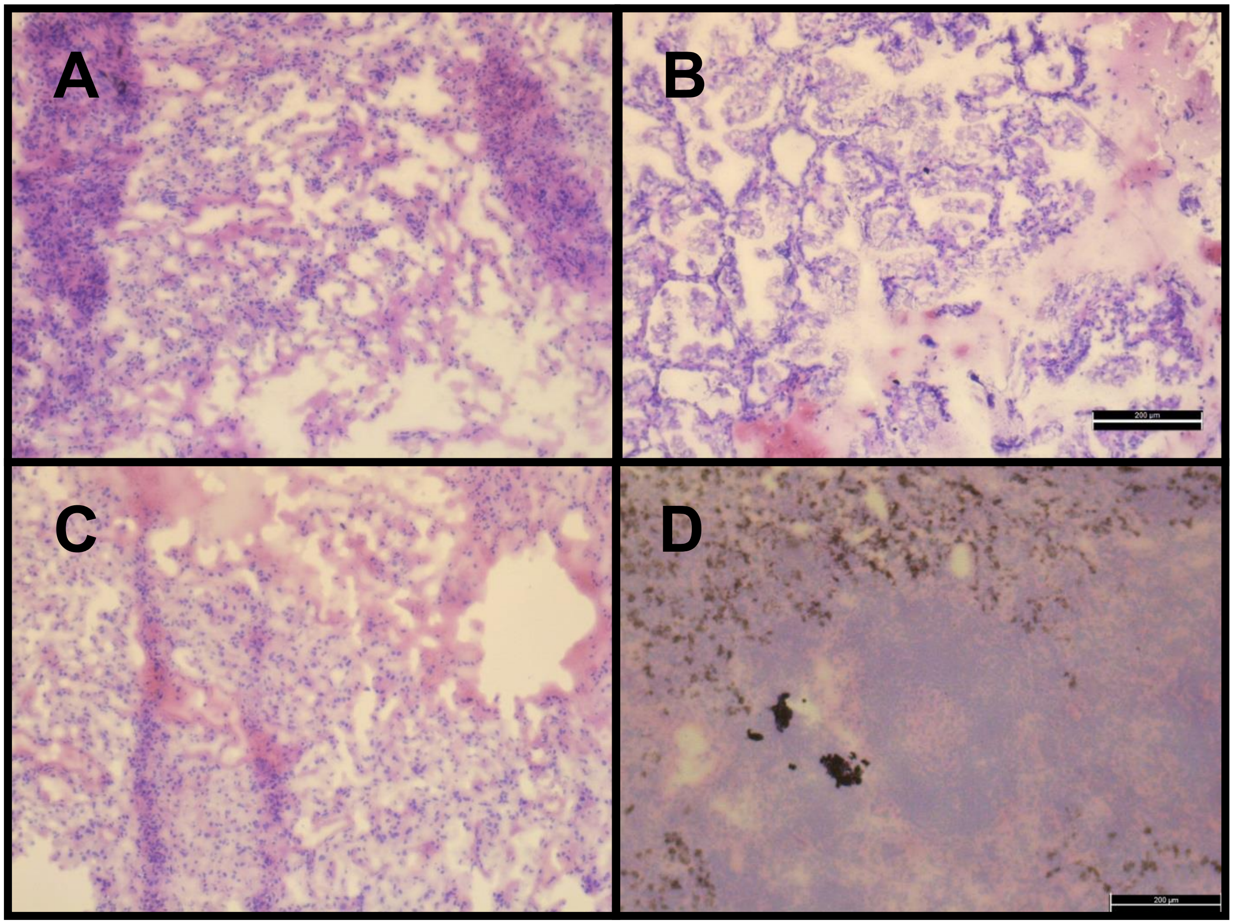


**A:** Normal lung, **B:** Lung adenocarcinoma, **C:** Reactive stroma (Tumor microenvironment, **D:** Lymph node

**Bar:** 200 µM

**Patient ID: 32**

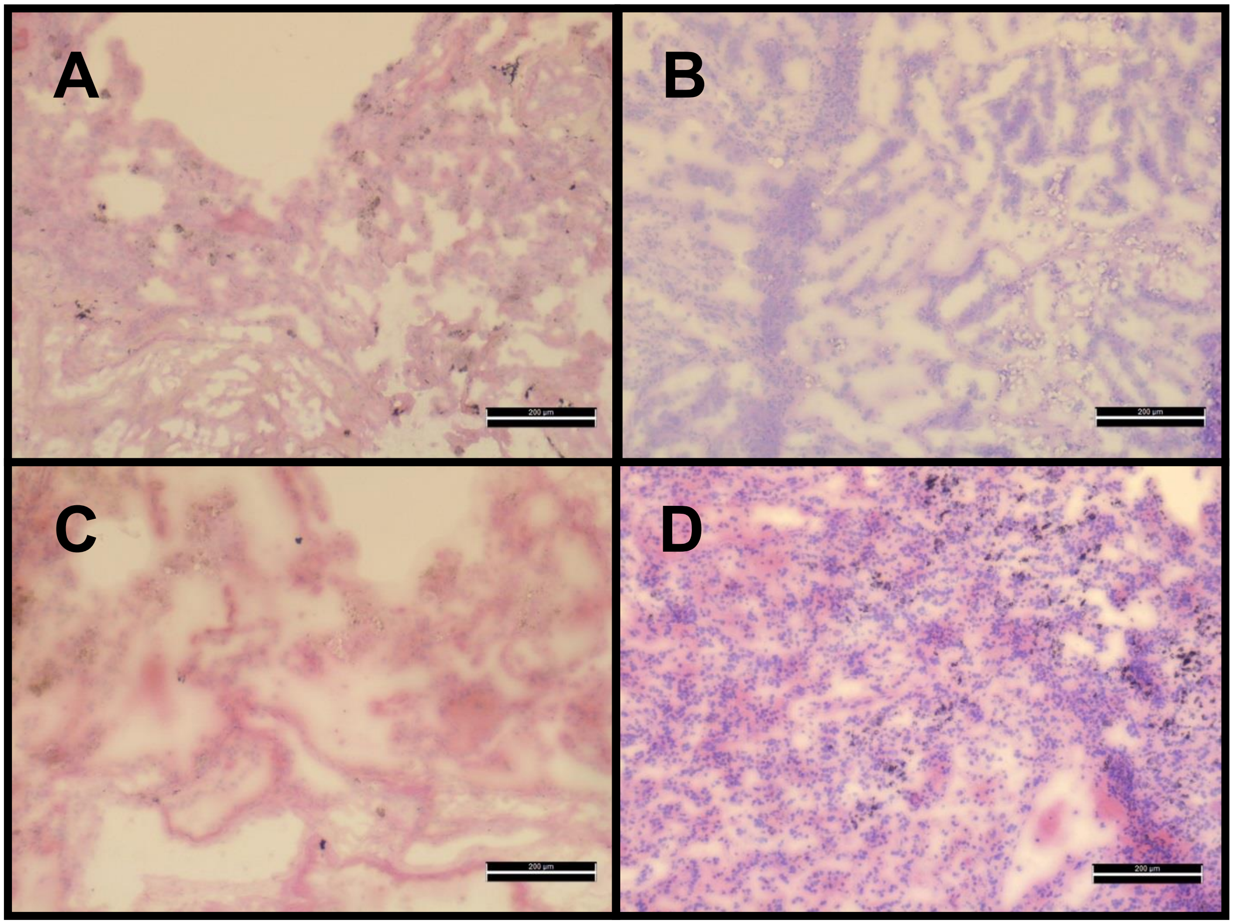


**A:** Normal lung, **B:** Lung adenocarcinoma, **C:** Reactive stroma (Tumor microenvironment, **D:** Lymph node

**Bar:** 200 μM

**Patient ID: 34**

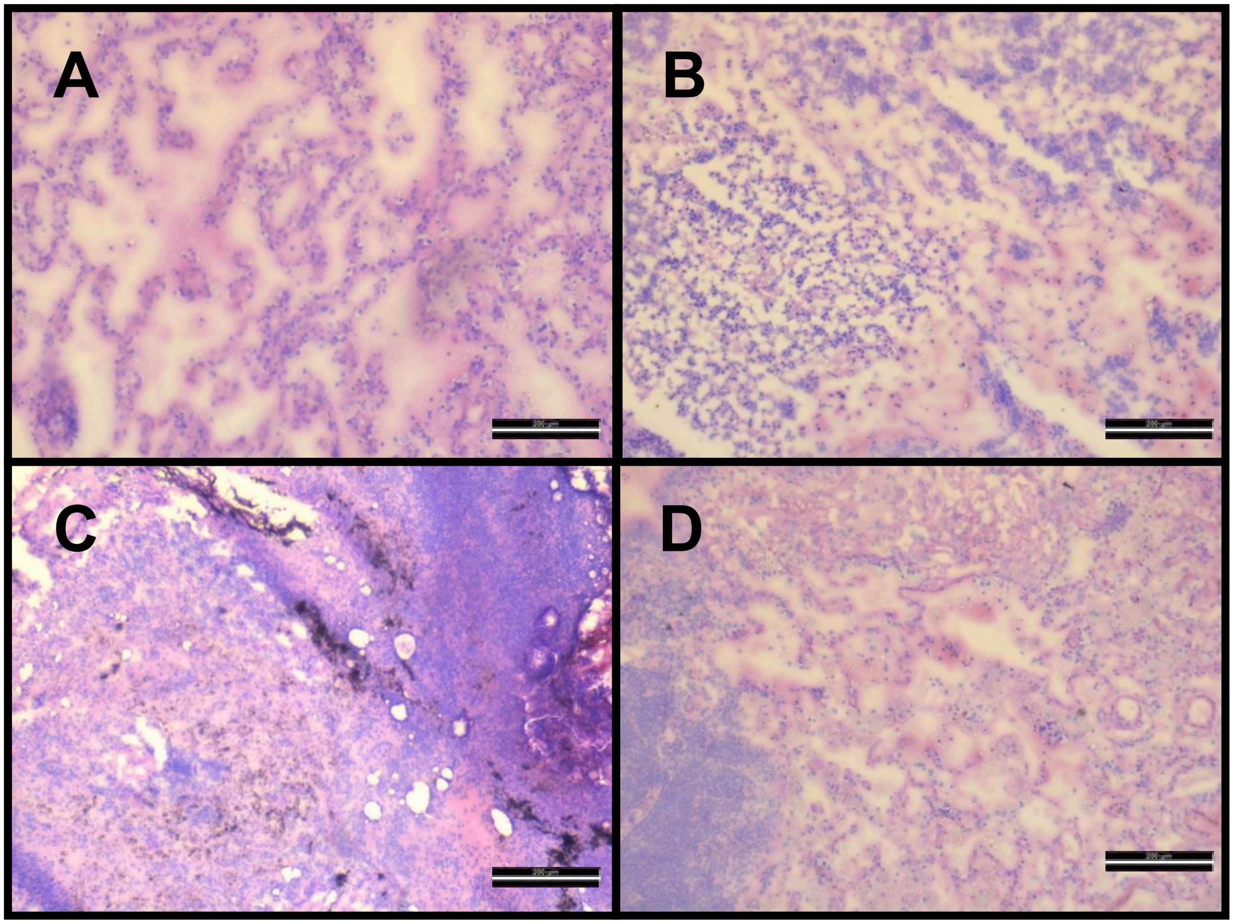


**A:** Normal lung, **B:** Large cell lung carcinoma, **C:** Reactive stroma (Tumor microenvironment, **D:** Lymph node

**Bar:** 200 μM

**Patient ID: 35**

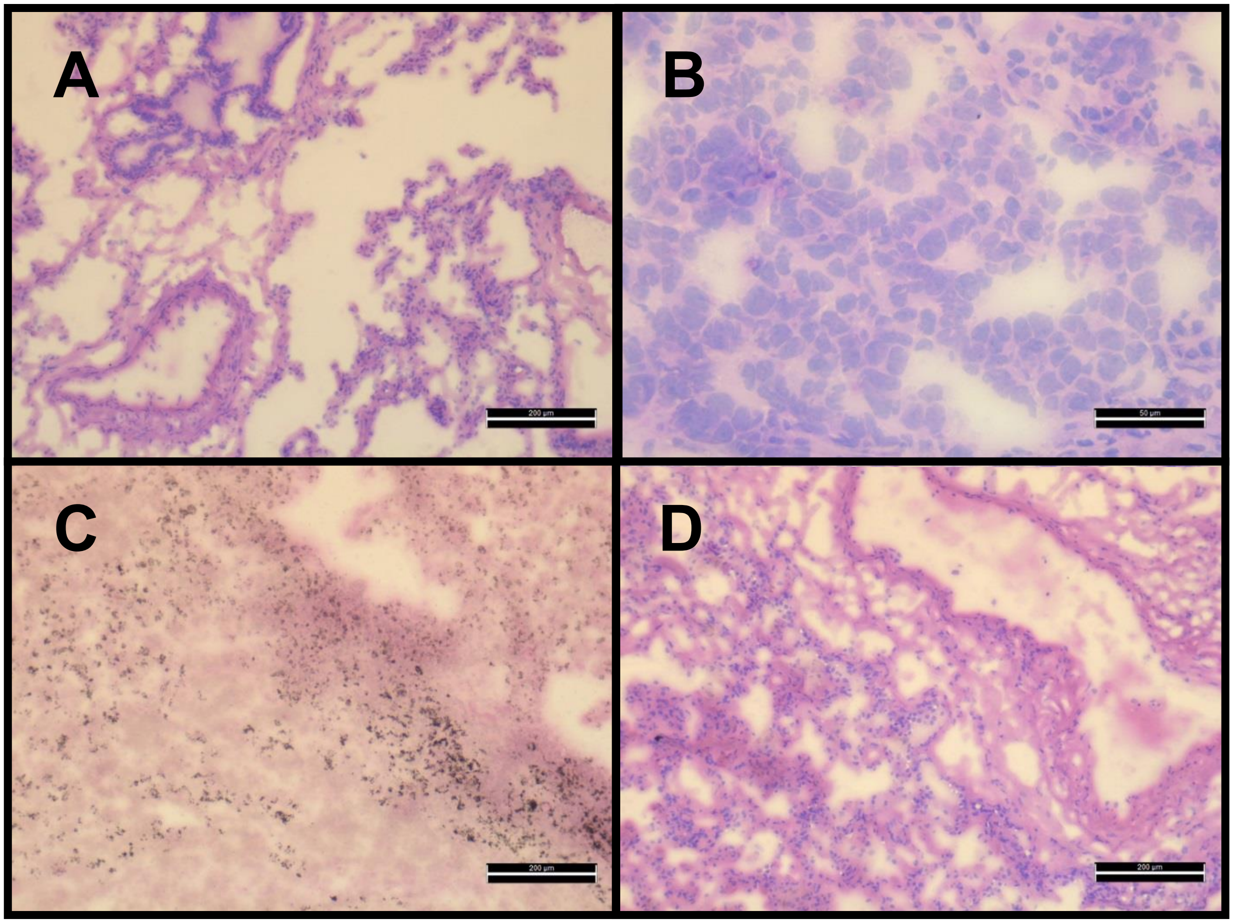


**A:** Normal lung, **B:** Lung adenocarcinoma, **C:** Reactive stroma (Tumor microenvironment, **D:** Lymph node

**Bar:** 200 μM

**Patient ID: 37**

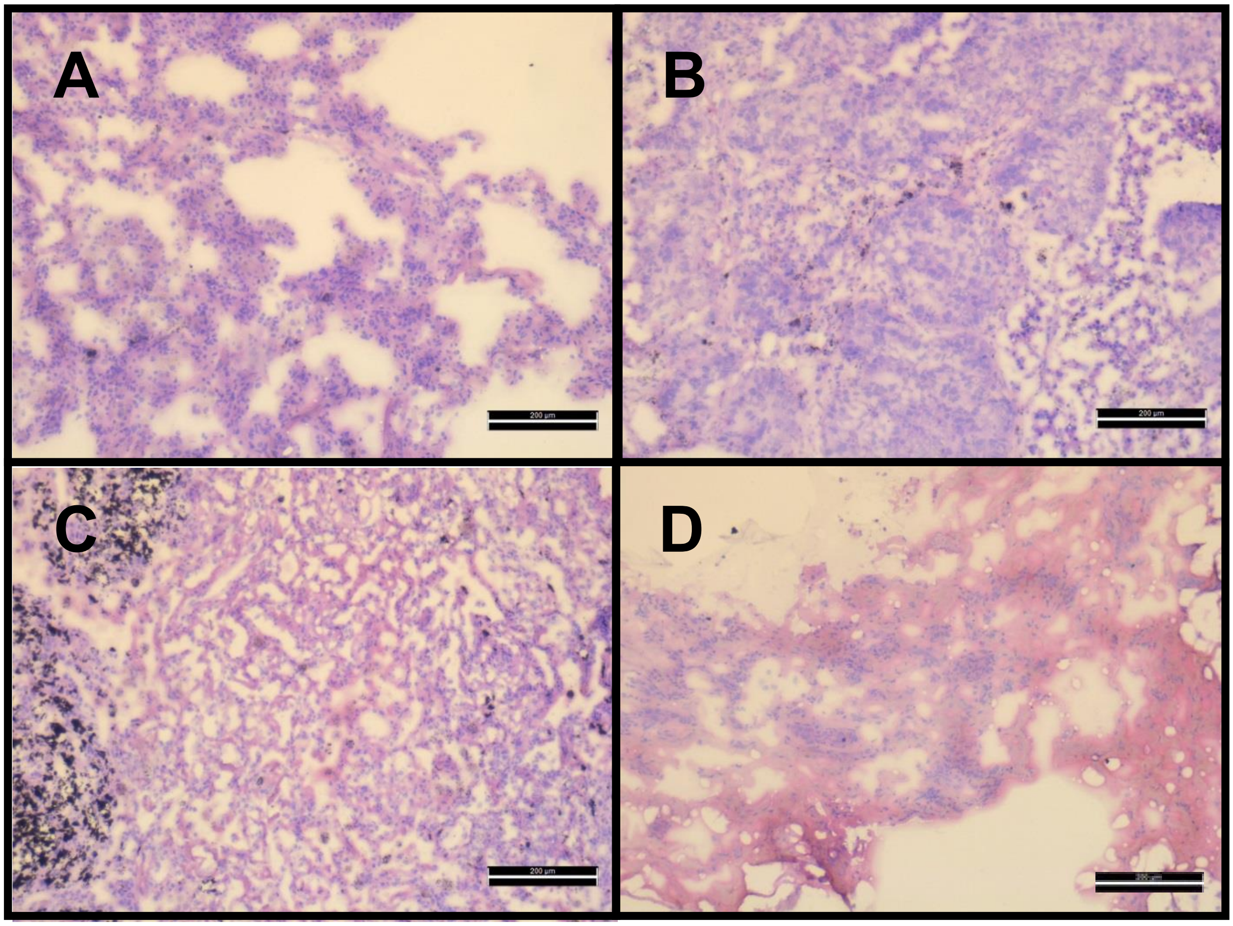


**A:** Normal lung, **B:** Squamous cell carcinoma, **C:** Reactive stroma (Tumor microenvironment, **D:** Lymph node

**Bar:** 200 μM

**Patient ID: 38**

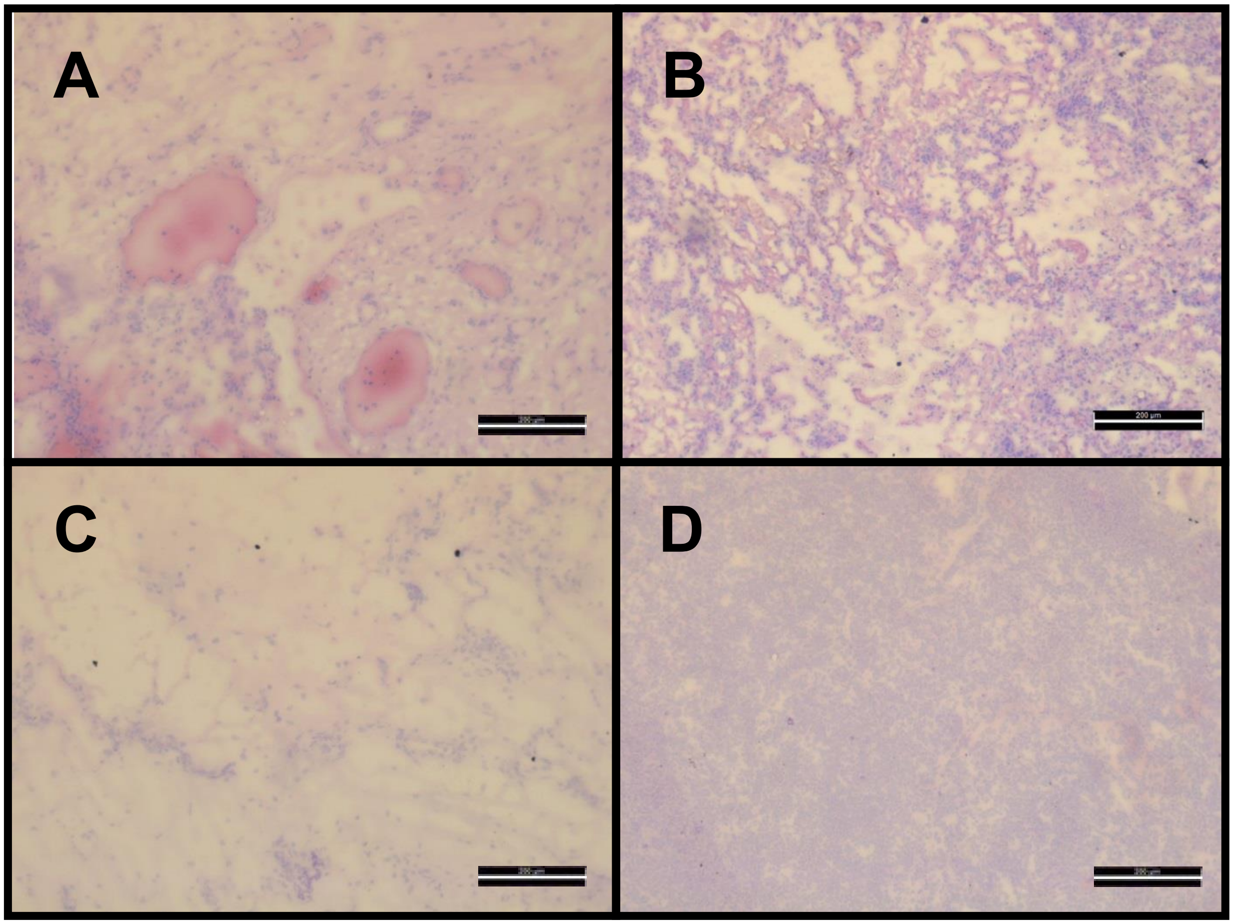


**A:** Normal lung, **B:** Squamous cell carcinoma, **C:** Reactive stroma (Tumor microenvironment, **D:** Lymph node

**Bar:** 200 μM

**Patient ID: 39**

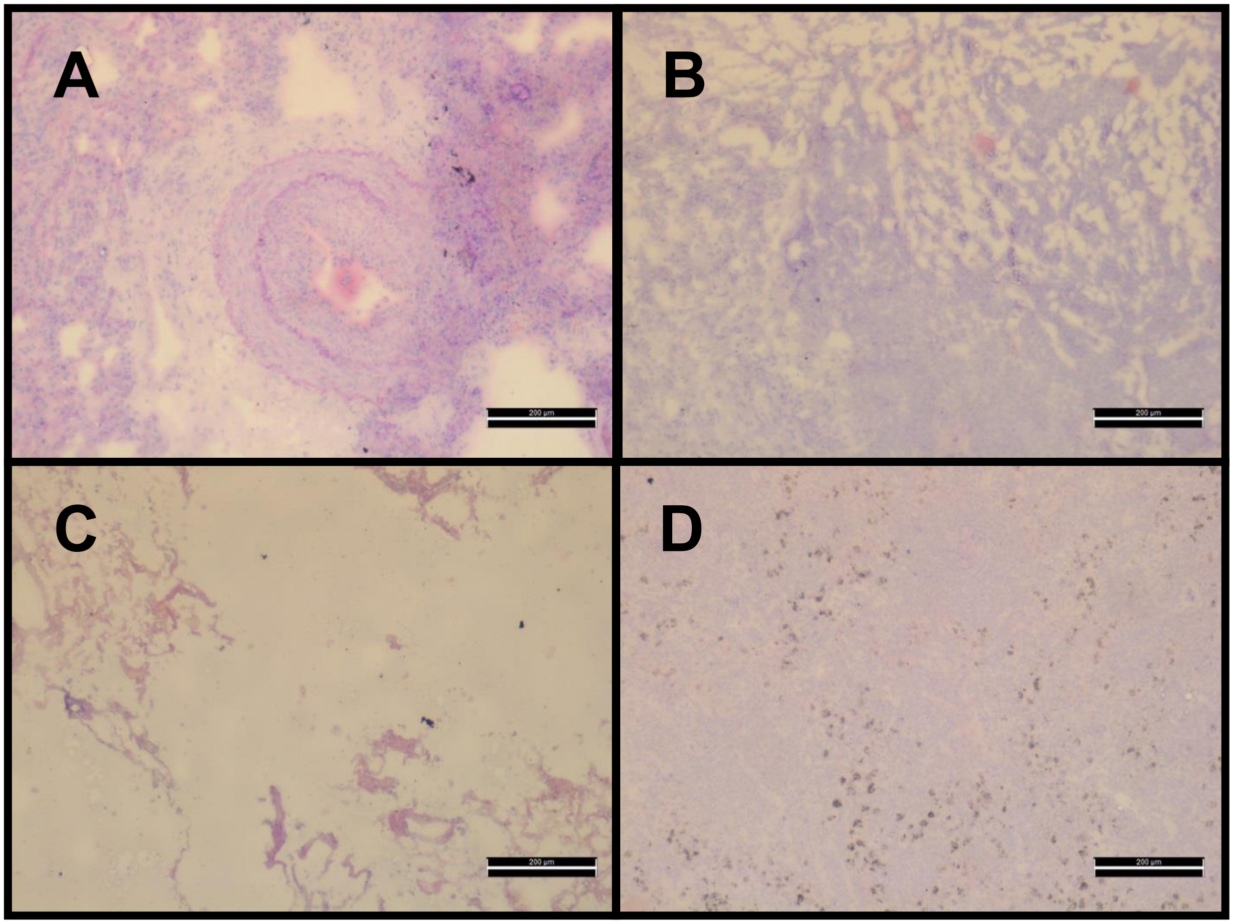


**A:** Normal lung, **B:** Squamous cell carcinoma, **C:** Reactive stroma (Tumor microenvironment, **D:** Lymph node

**Bar:** 200 µM

**Patient ID: 41**

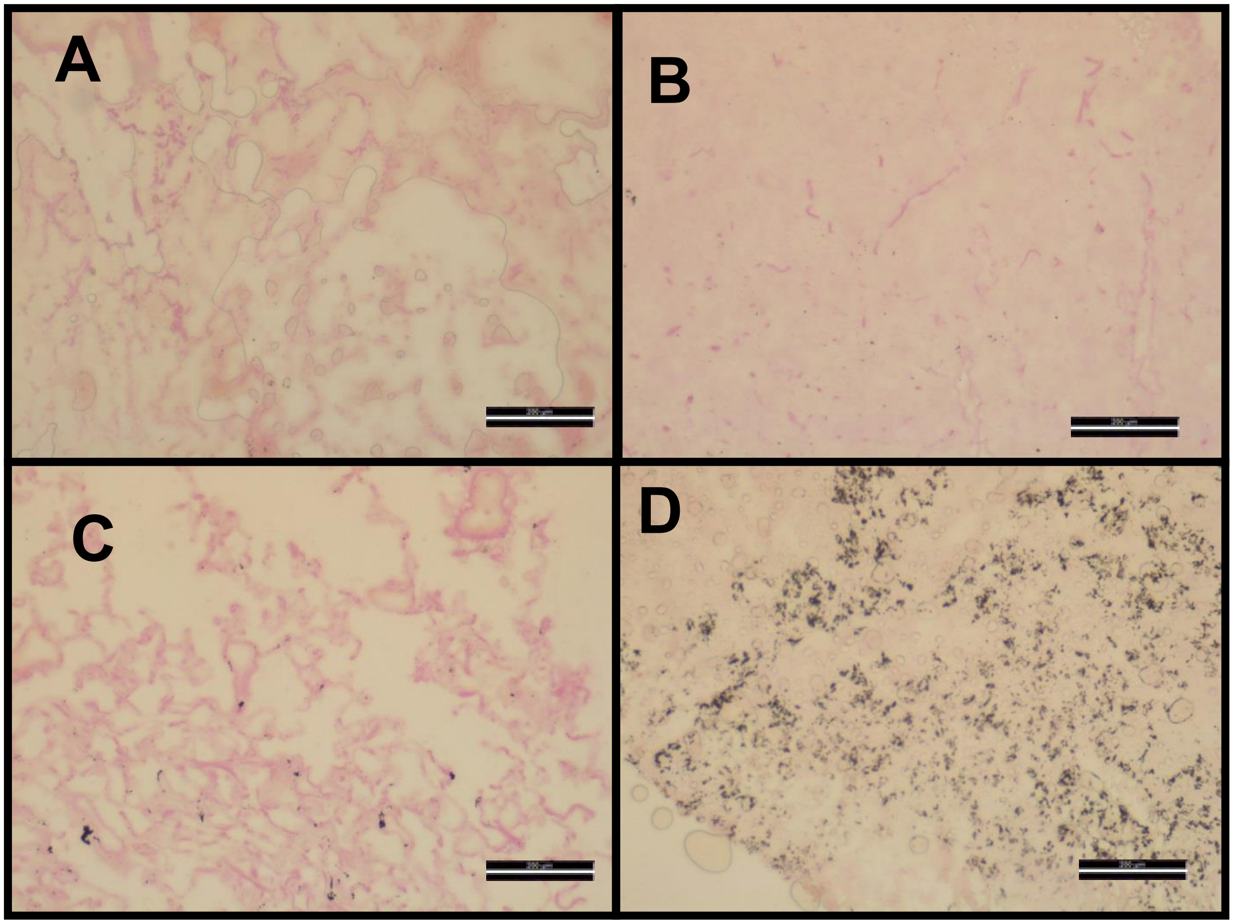


**A:** Normal lung, **B:** Lung adenocarcinoma, **C:** Reactive stroma (Tumor microenvironment, **D:** Lymph node

**Bar:** 200 μM

**Patient ID: 44**

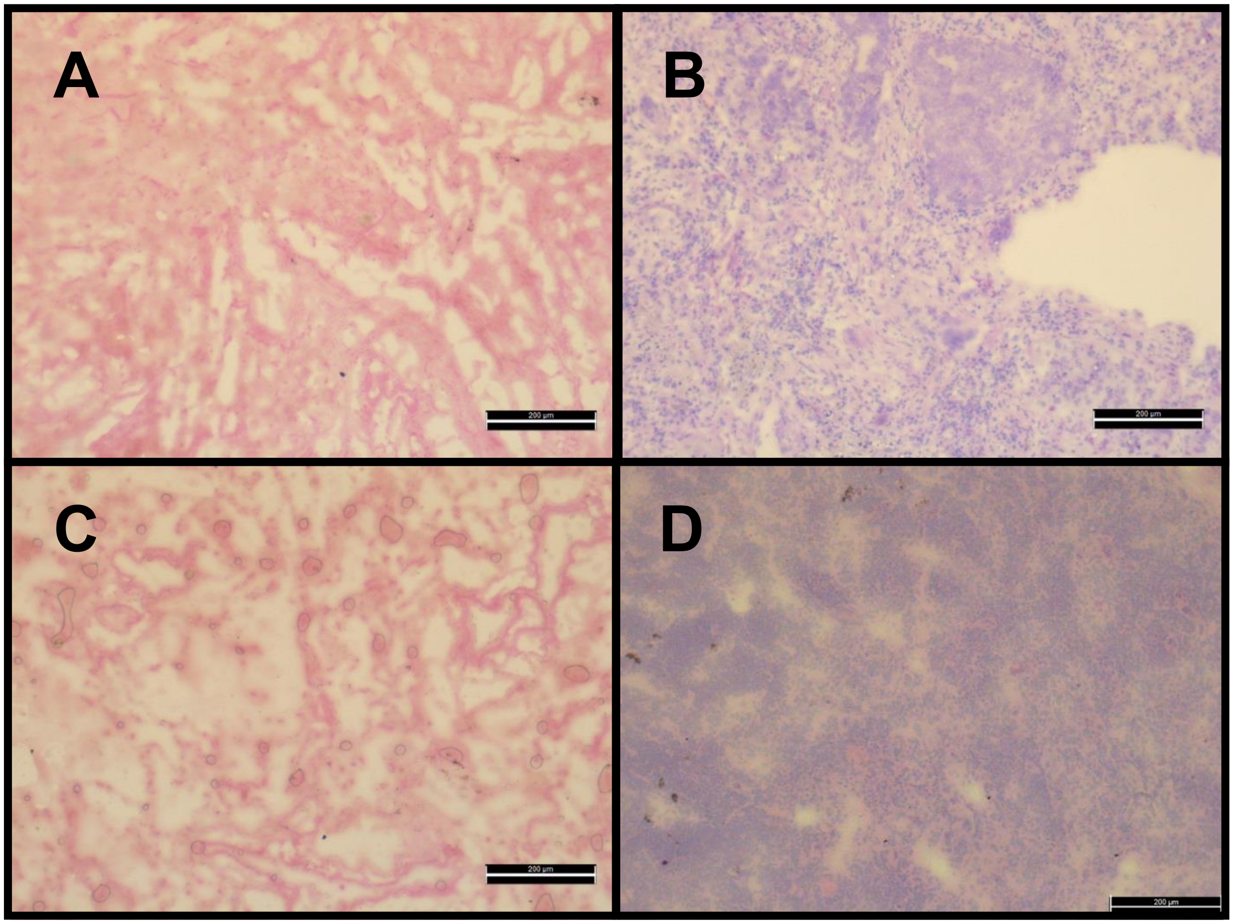


**A:** Normal lung, **B:** Lung adenocarcinoma, **C:** Reactive stroma (Tumor microenvironment, **D:** Lymph node

**Bar:** 200 µM

**Patient ID: 47**

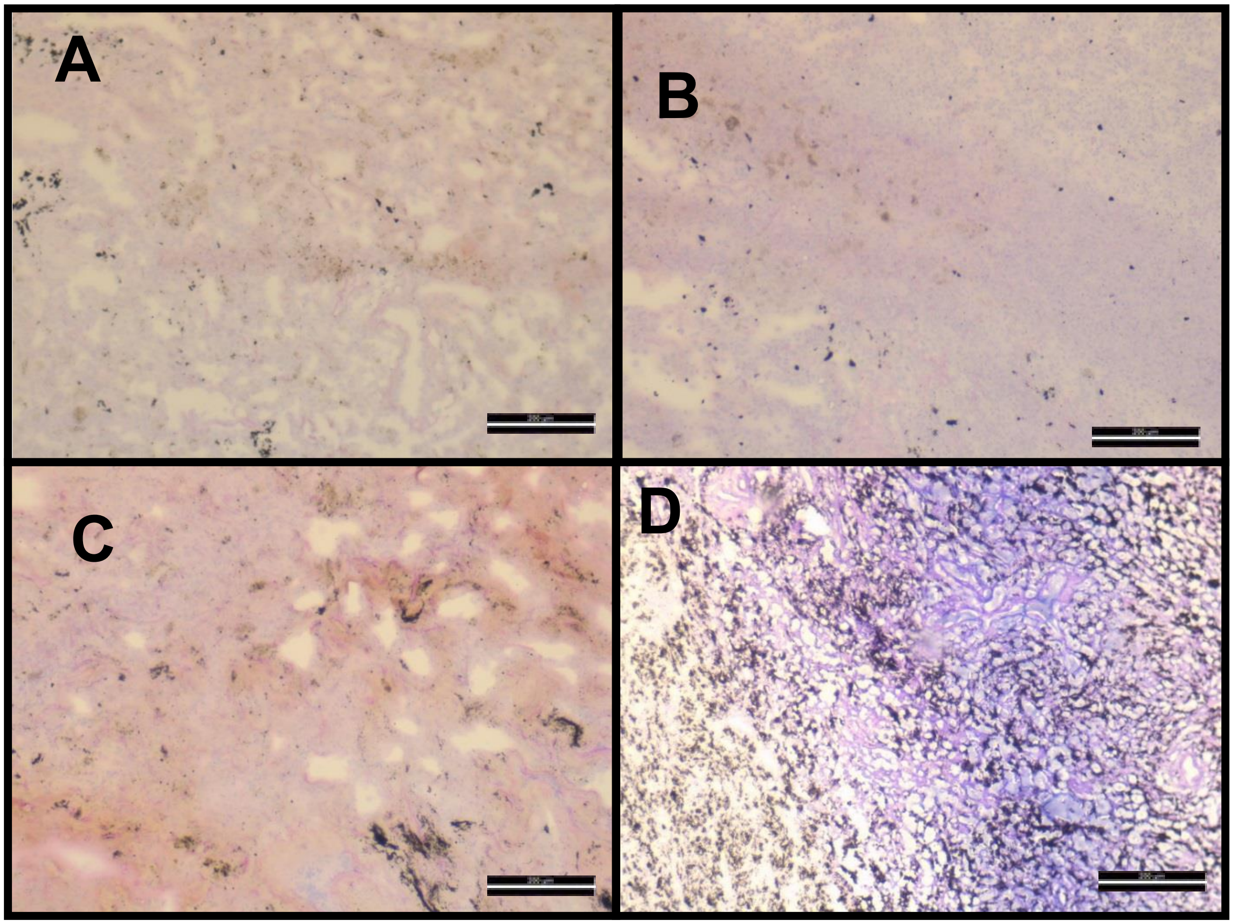


**A:** Normal lung, **B:** Lung adenocarcinoma, **C:** Reactive stroma (Tumor microenvironment, **D:** Lymph node

**Bar:** 200 μM

**Patient ID: 49**

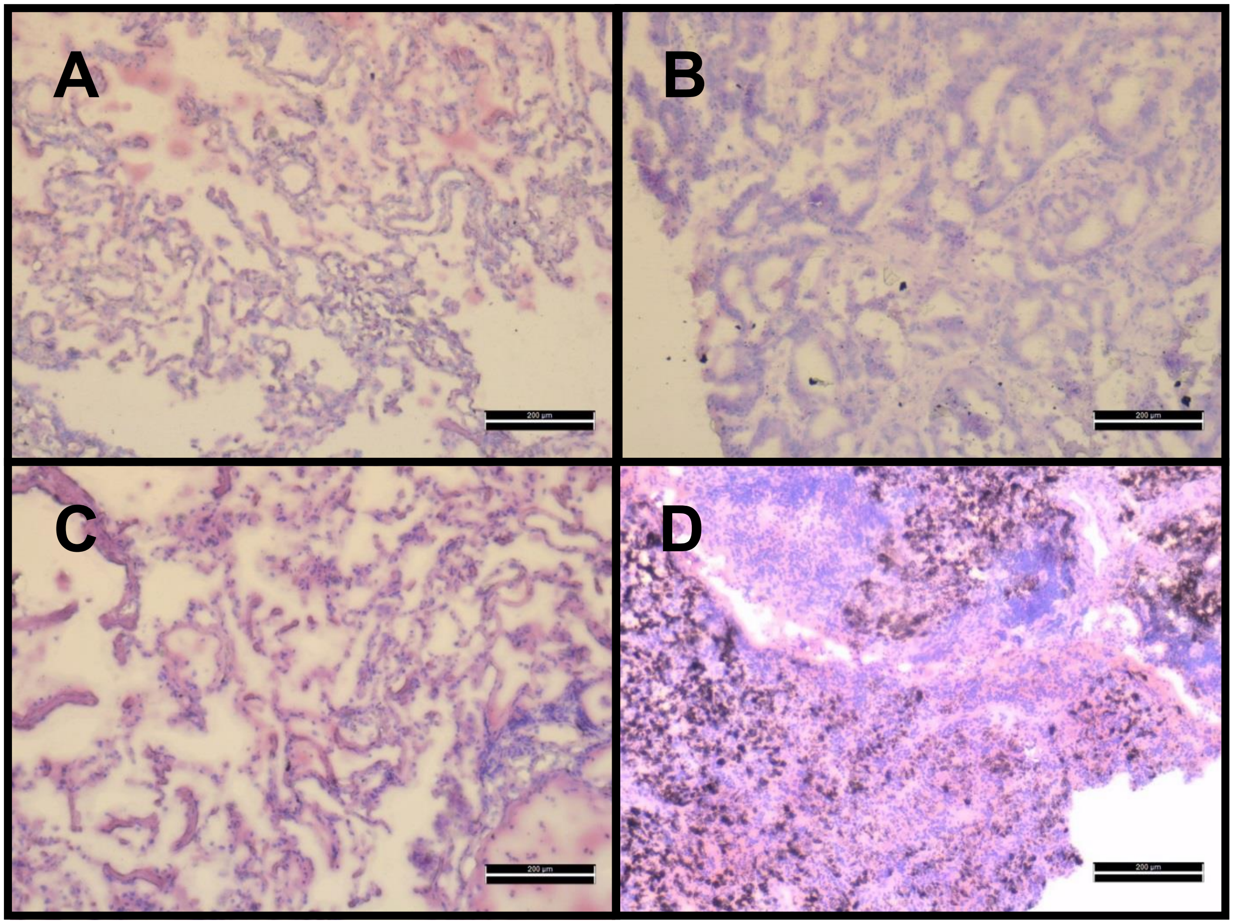


**A:** Normal lung, **B:** Lung adenocarcinoma, **C:** Reactive stroma (Tumor microenvironment, **D:** Lymph node

**Bar:** 200 µM

**Patient ID: 51**

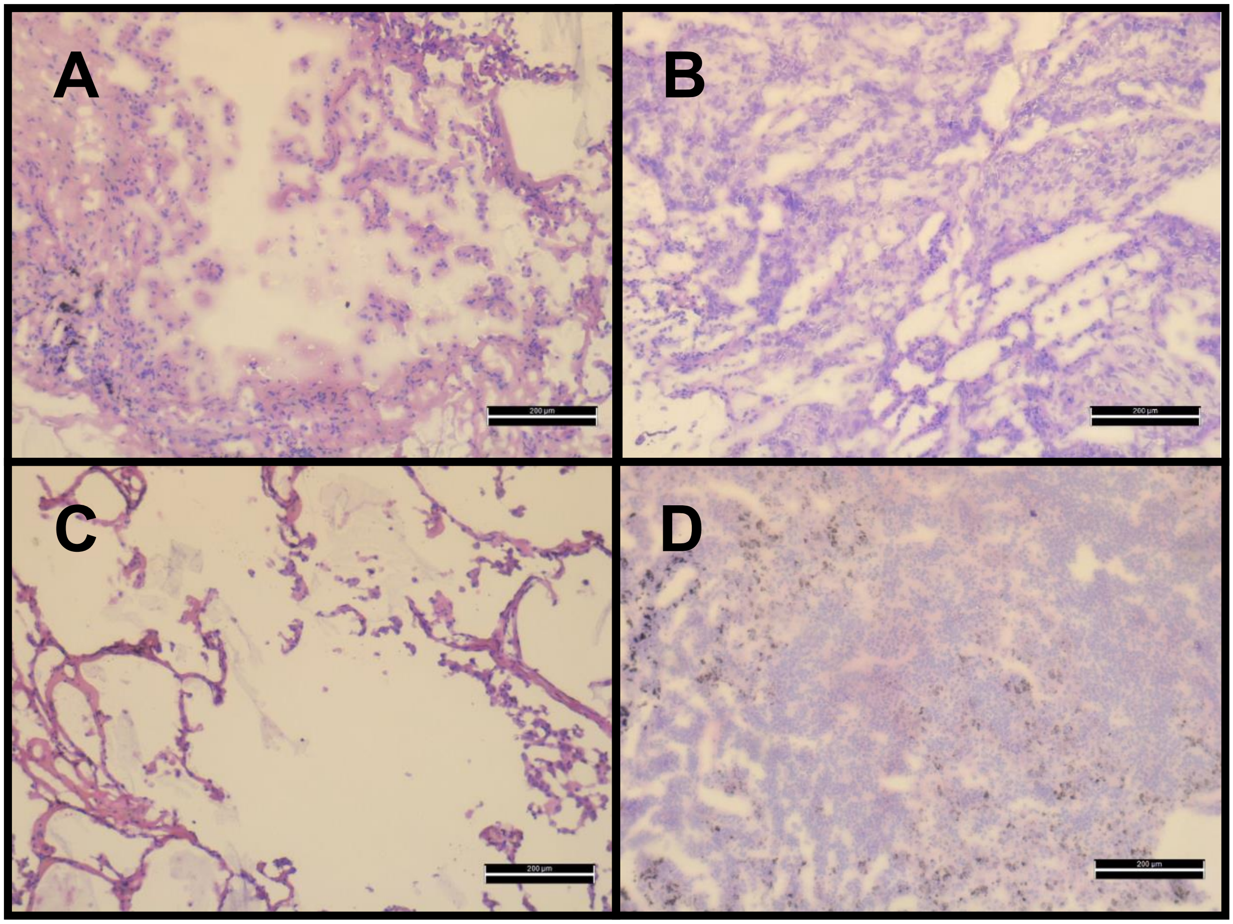


**A:** Normal lung, **B:** Squamous cell carcinoma, **C:** Reactive stroma (Tumor microenvironment, **D:** Lymph node

**Bar:** 200 µM

**Patient ID: 54**

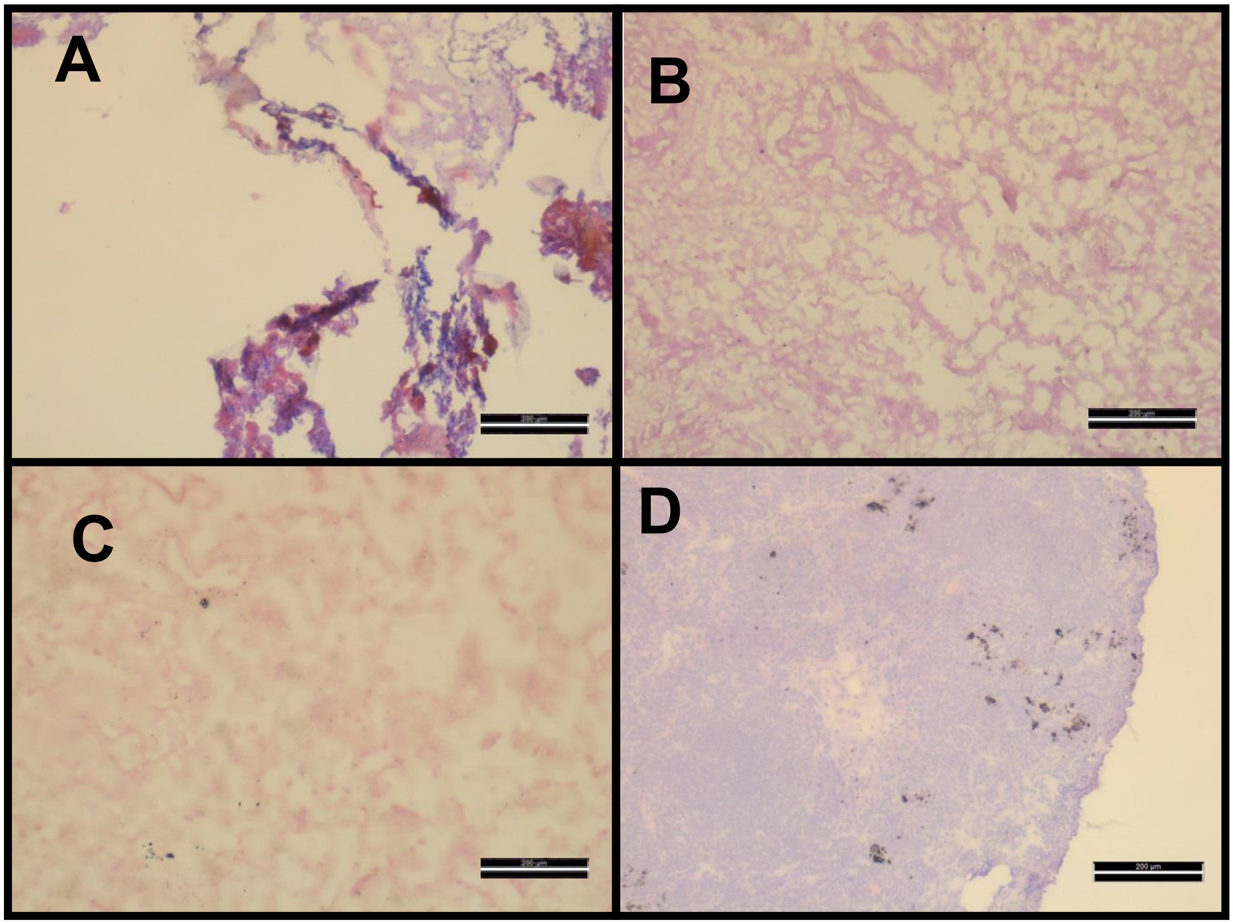


**A:** Normal lung, **B:** Squamous cell carcinoma, **C:** Reactive stroma (Tumor microenvironment, **D:** Lymph node

**Bar:** 200 μM

**Patient ID: 55**

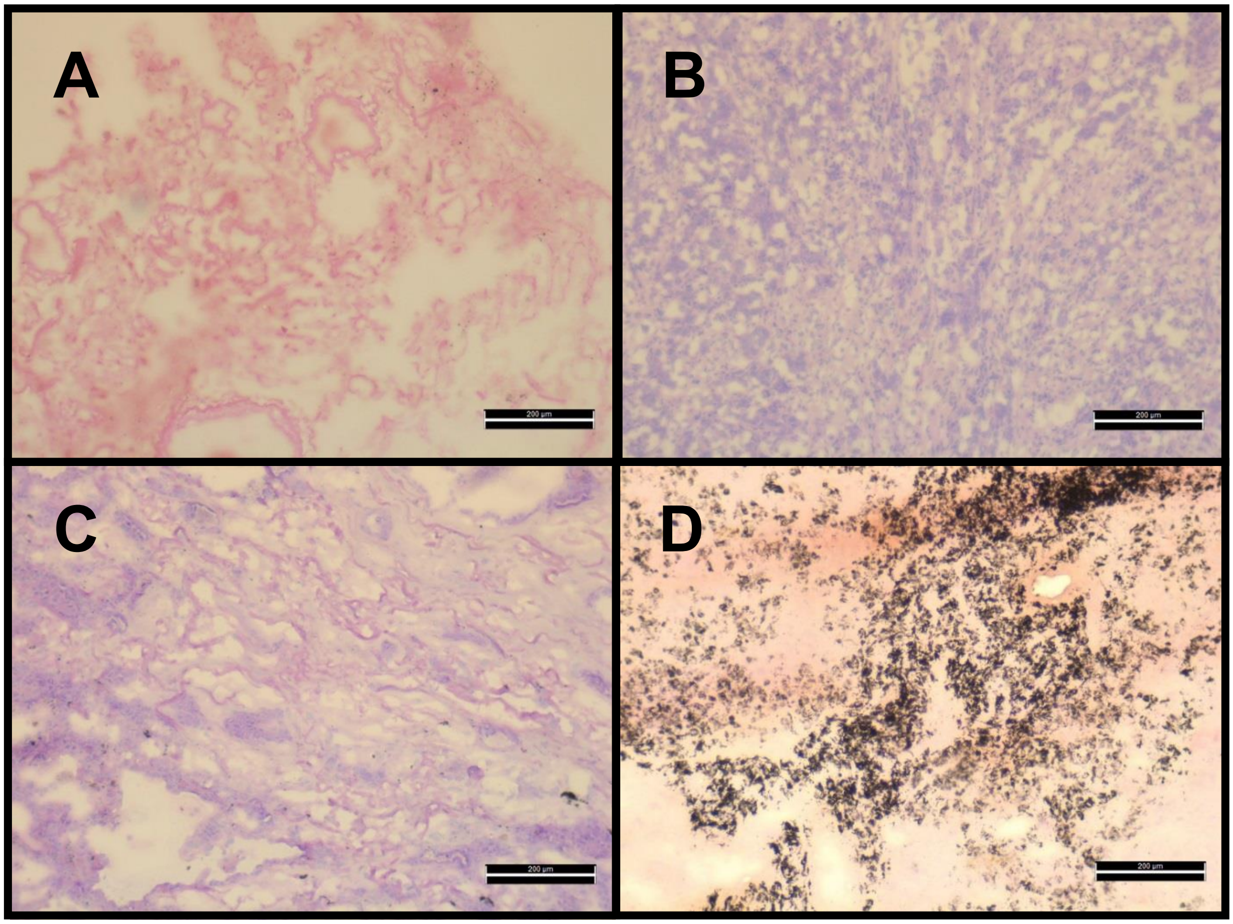


**A:** Normal lung, **B:** Lung adenocarcinoma, **C:** Reactive stroma (Tumor microenvironment, **D:** Lymph node

**Bar:** 200 µM

**Patient ID: 56**

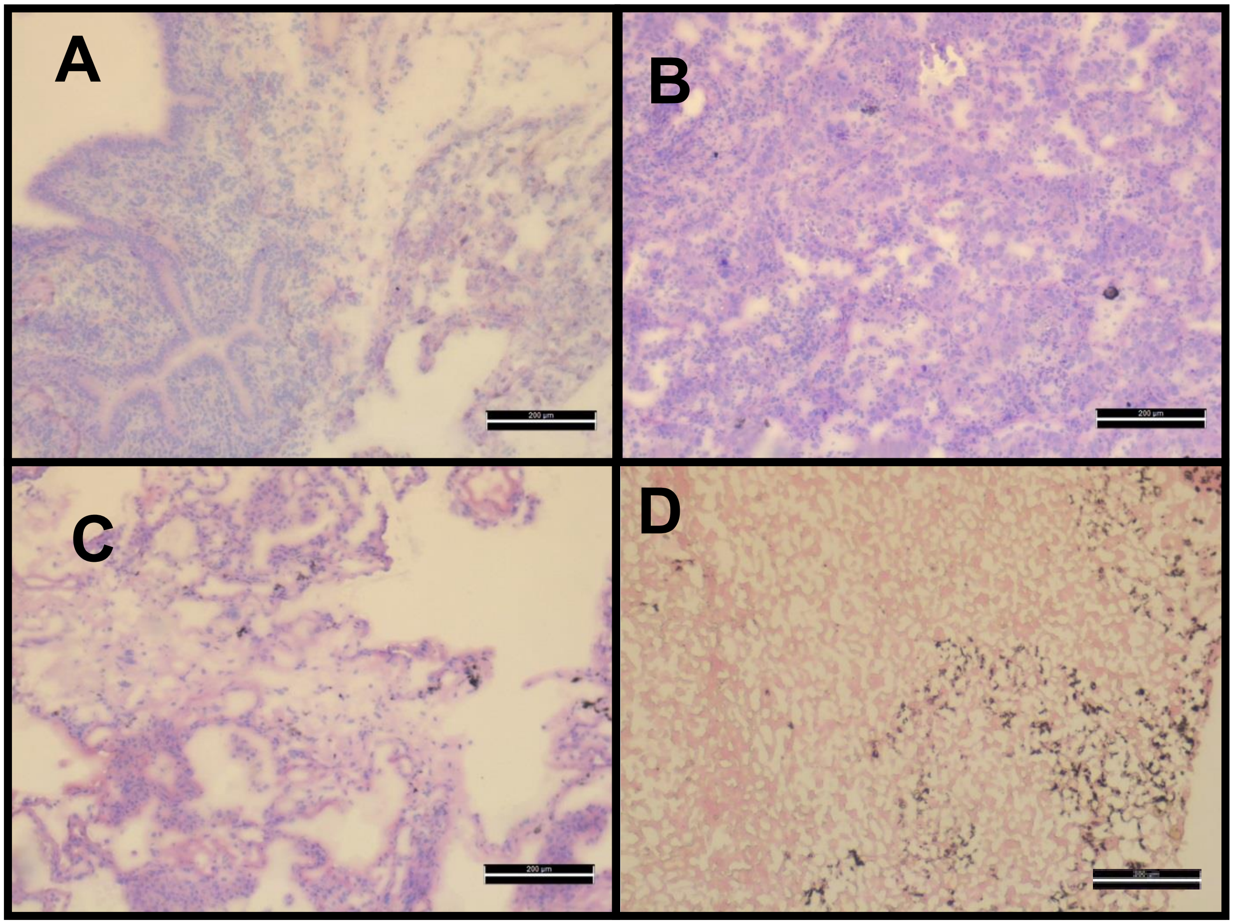


**A:** Normal lung, **B:** Squamous cell carcinoma, **C:** Reactive stroma (Tumor microenvironment, **D:** Lymph node

**Bar:** 200 μM

**Patient ID: 61**

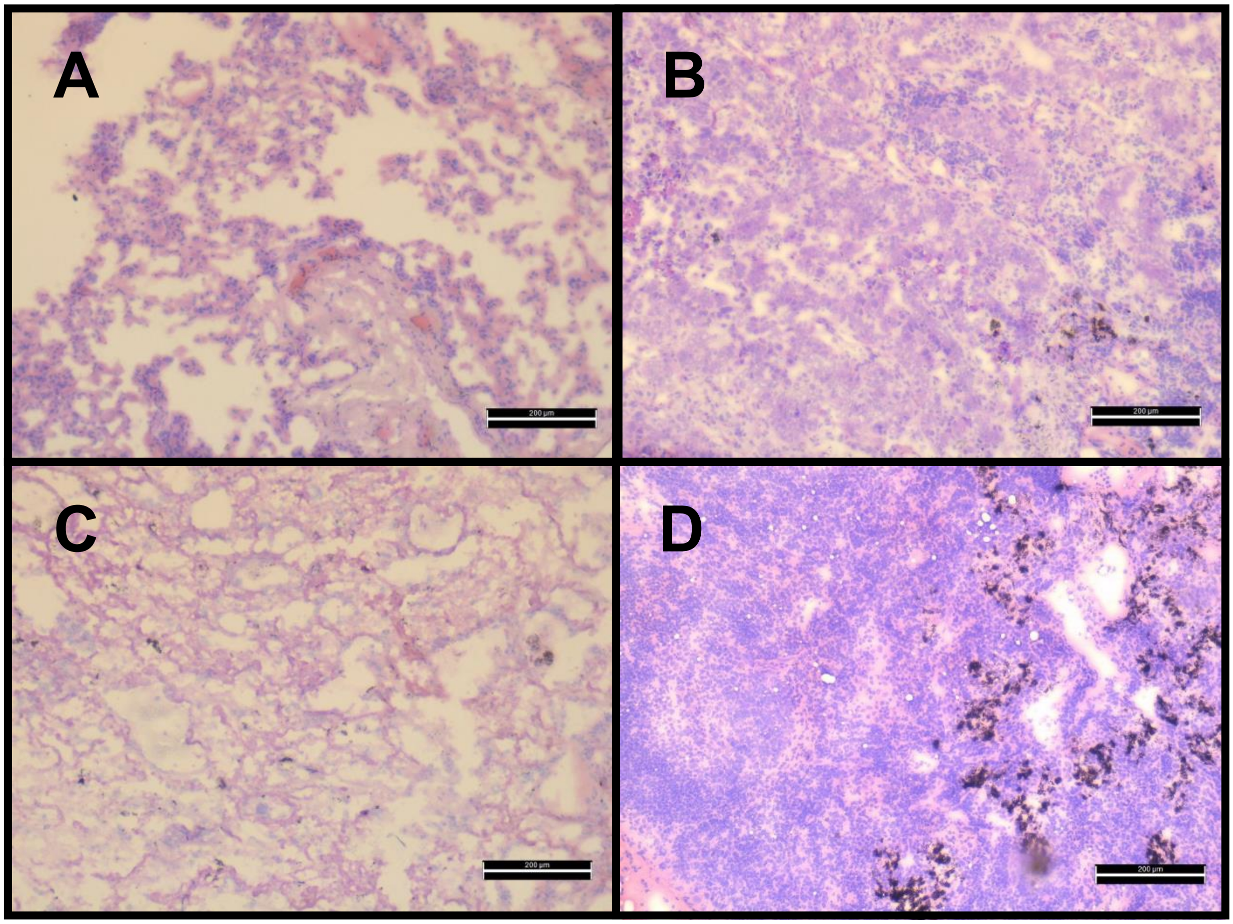


**A:** Normal lung, **B:** Lung adenocarcinoma, **C:** Reactive stroma (Tumor microenvironment, **D:** Lymph node

**Bar:** 200 µM

**Patient ID: 63**

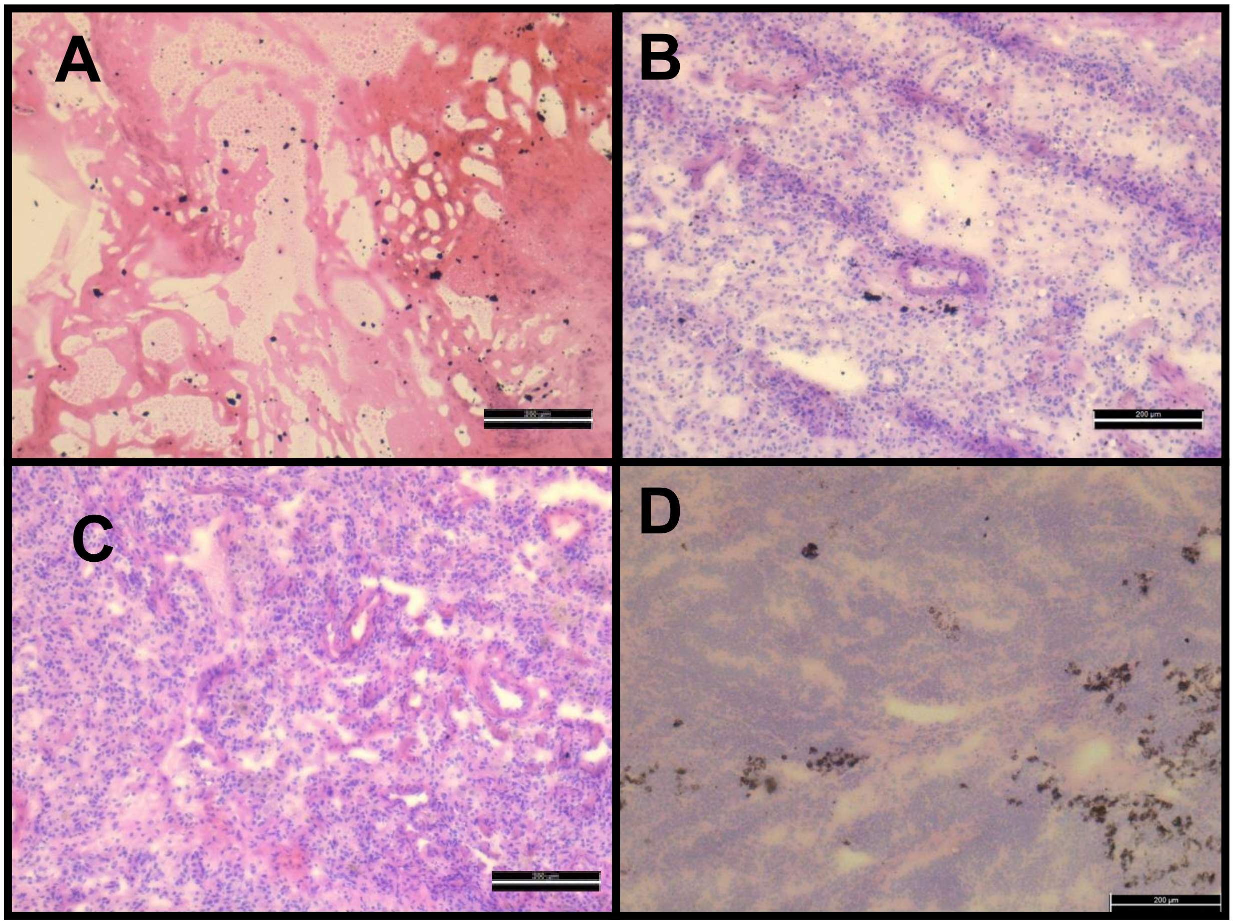


**A:** Normal lung, **B:** Squamous cell carcinoma, **C:** Reactive stroma (Tumor microenvironment, **D:** Lymph node

**Bar:** 200 μM

**Patient ID: 68**

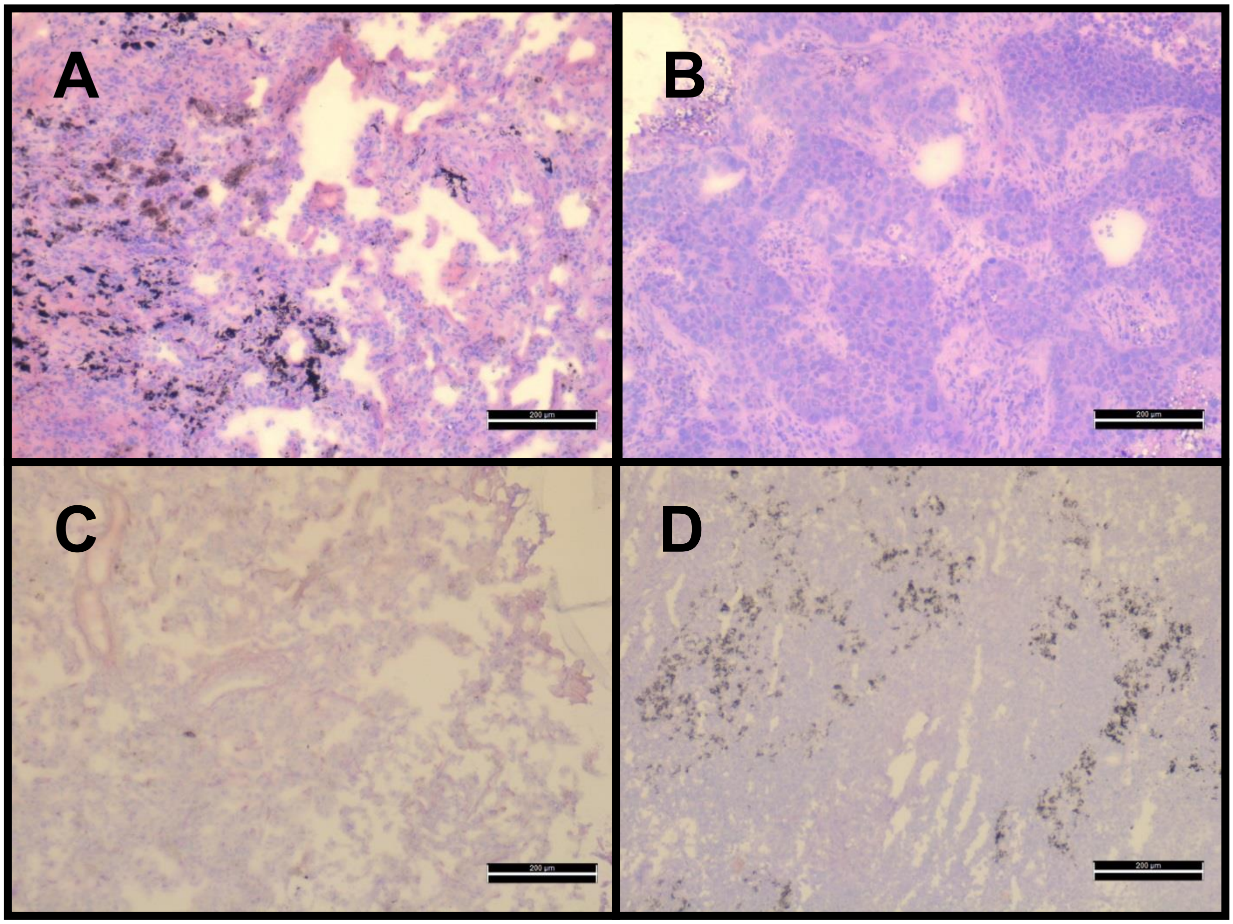


**A:** Normal lung, **B:** Squamous cell carcinoma, **C:** Reactive stroma (Tumor microenvironment, **D:** Lymph node

**Bar:** 200 μM

**Patient ID: 70**

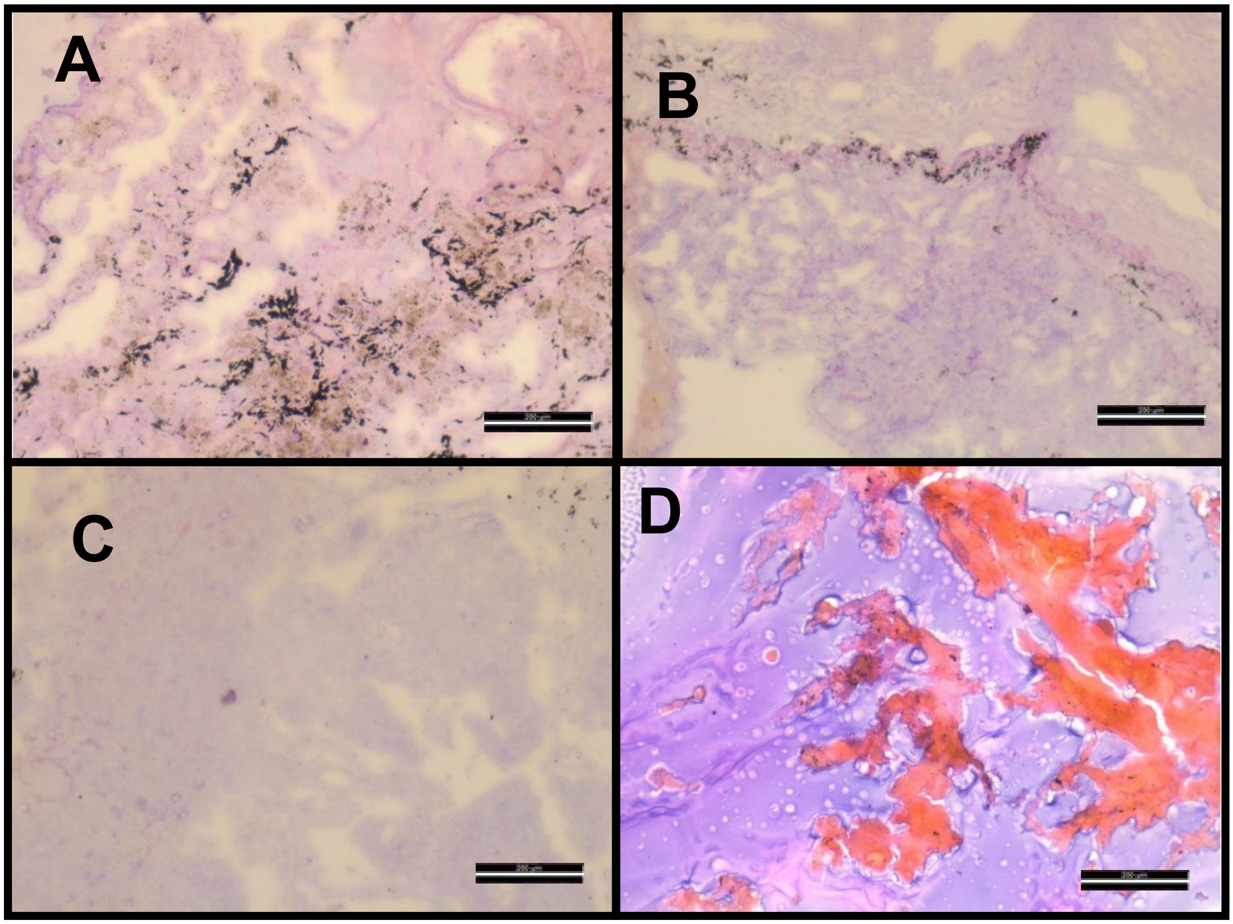


**A:** Normal lung, **B:** Lung adenocarcinoma, **C:** Reactive stroma (Tumor microenvironment, **D:** Lymph node

**Bar:** 200 μM

**Patient ID: 71**

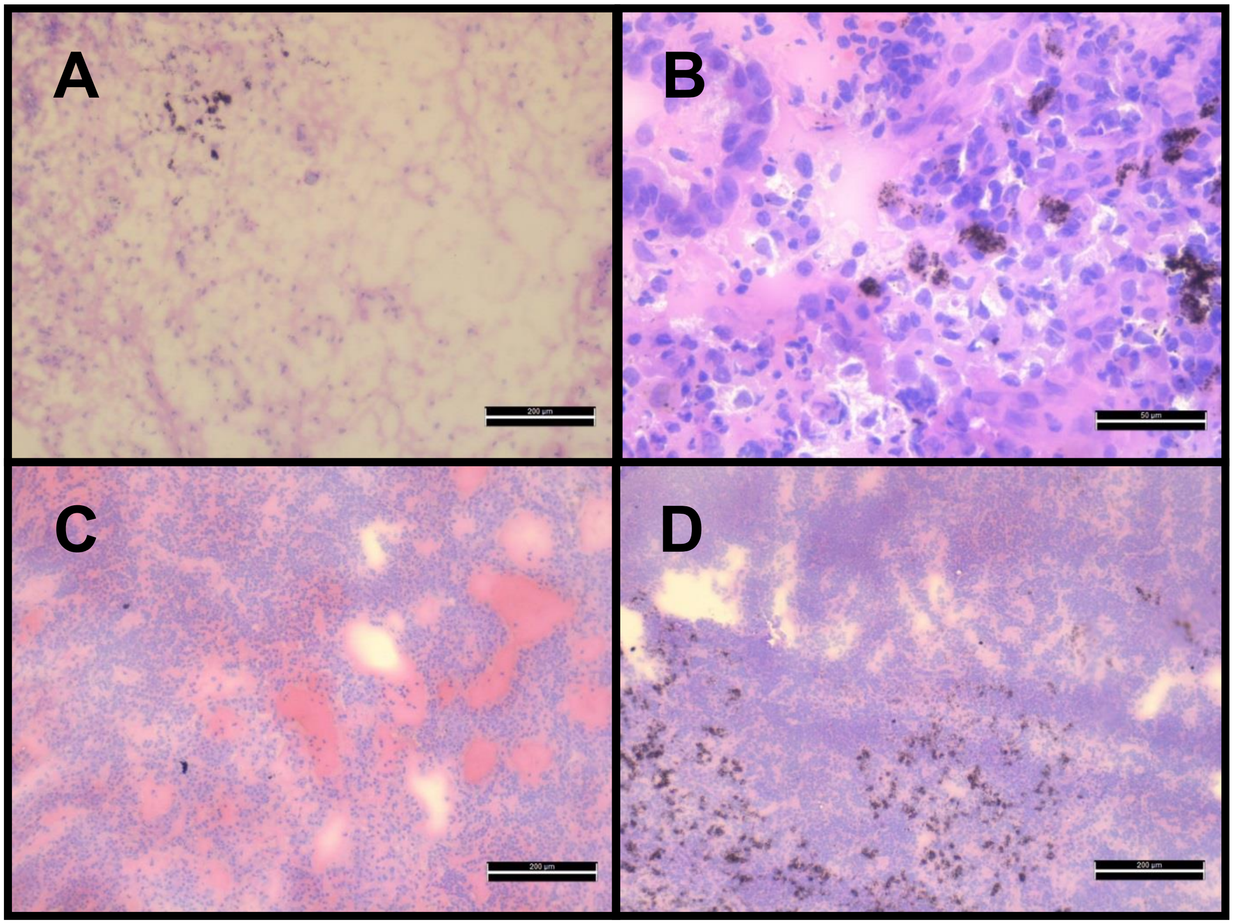


**A:** Normal lung, **B:** Squamous cell carcinoma, **C:** Reactive stroma (Tumor microenvironment, **D:** Lymph node

**Bar:** 200 µM

**Patient ID: 78**

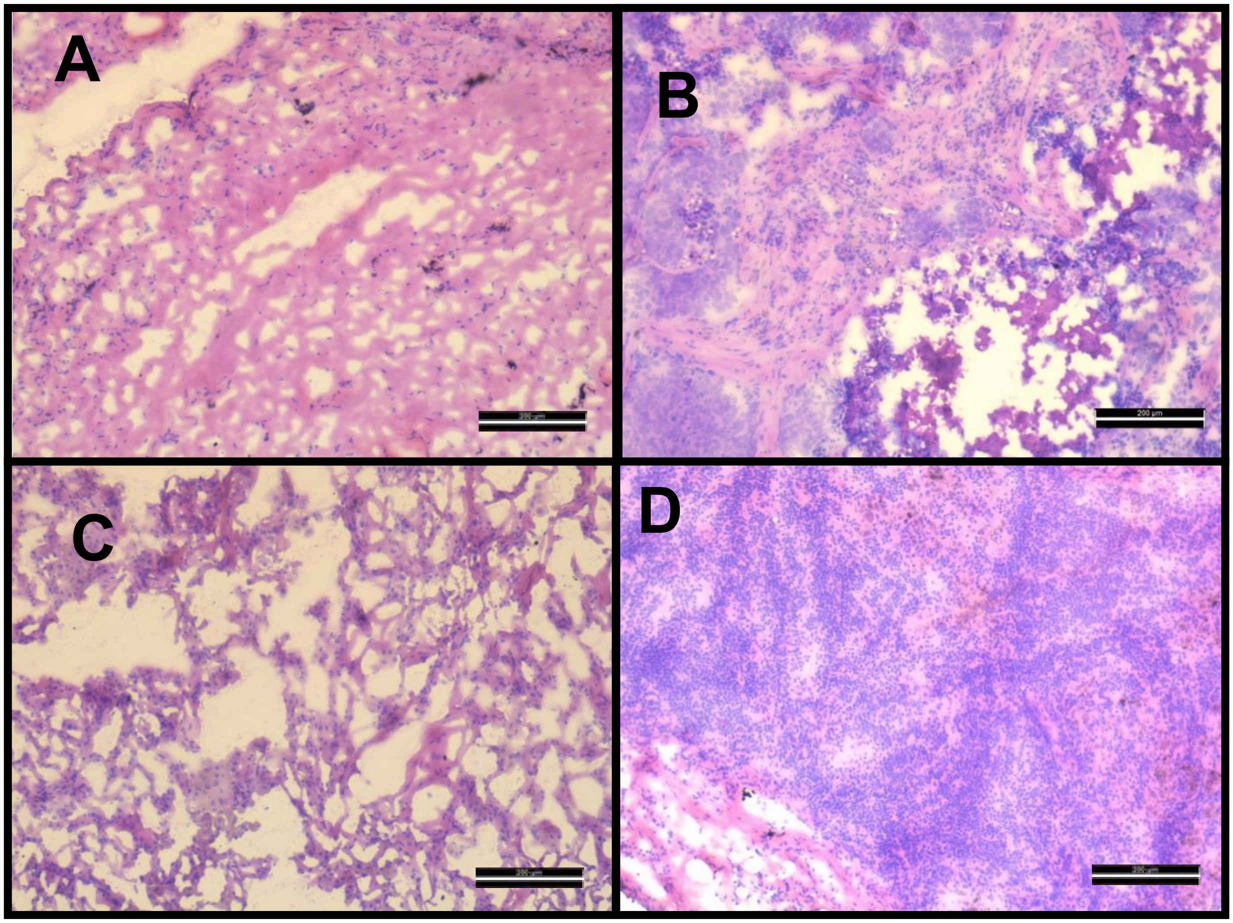


**A:** Normal lung, **B:** Large cell lung cancer, **C:** Reactive stroma (Tumor microenvironment, **D:** Lymph node

**Bar:** 200 µM

**Patient ID: 90**

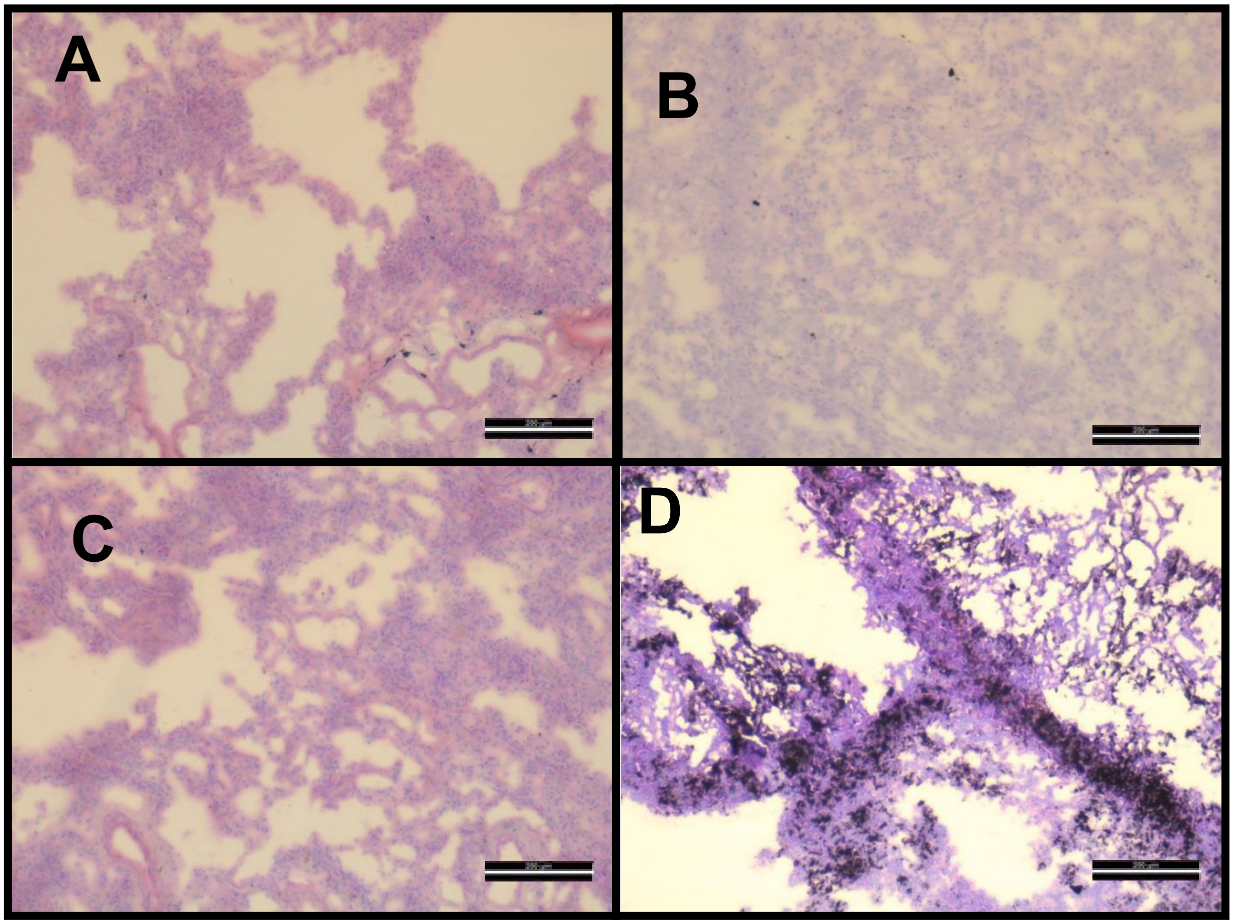


**A:** Normal lung, **B:** Lung adenocarcinoma, **C:** Reactive stroma (Tumor microenvironment, **D:** Lymph node

**Bar:** 200 µM

**Patient ID: 102**

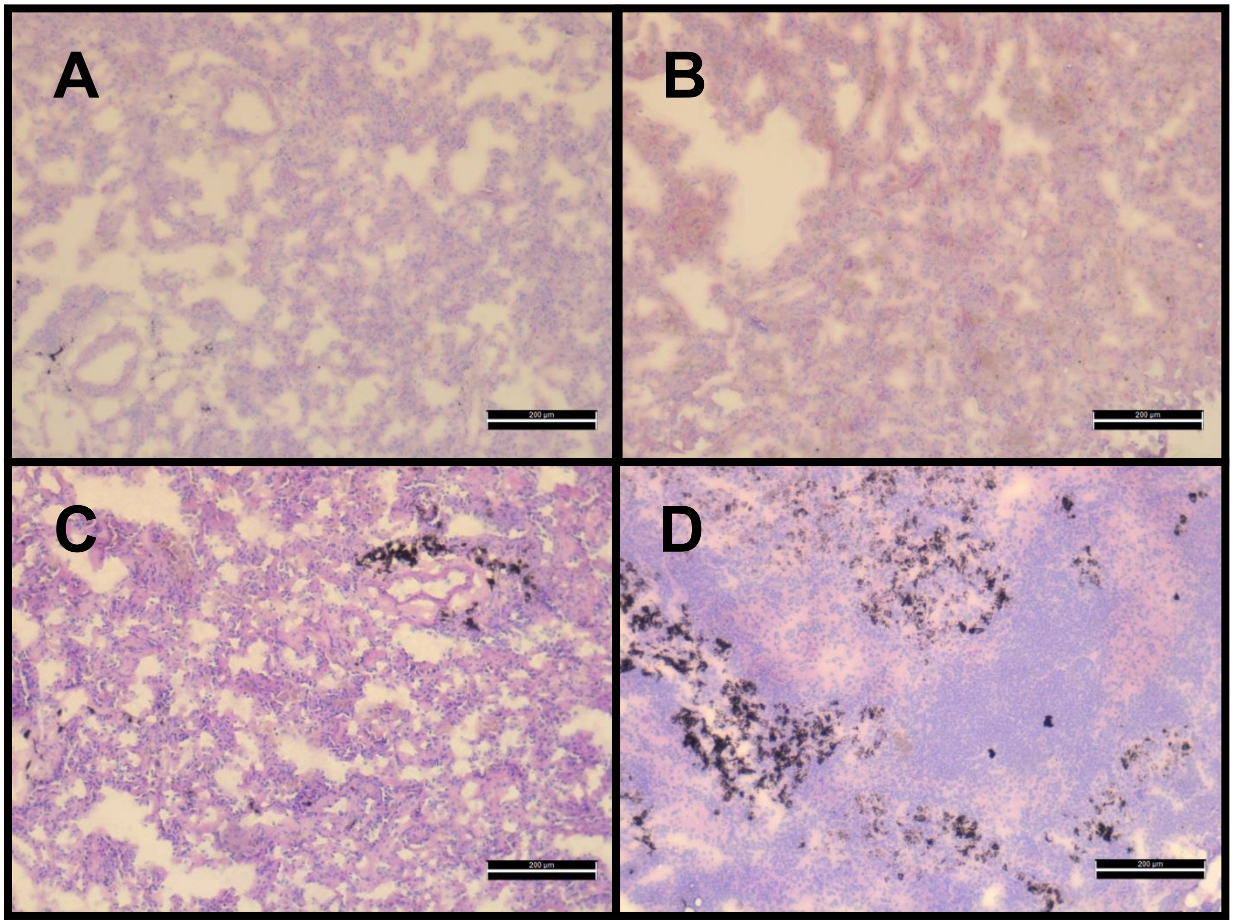


**A:** Normal lung, **B:** Lung adenocarcinoma, **C:** Reactive stroma (Tumor microenvironment, **D:** Lymph node

**Bar:** 200 μM

**Patient ID: 105**

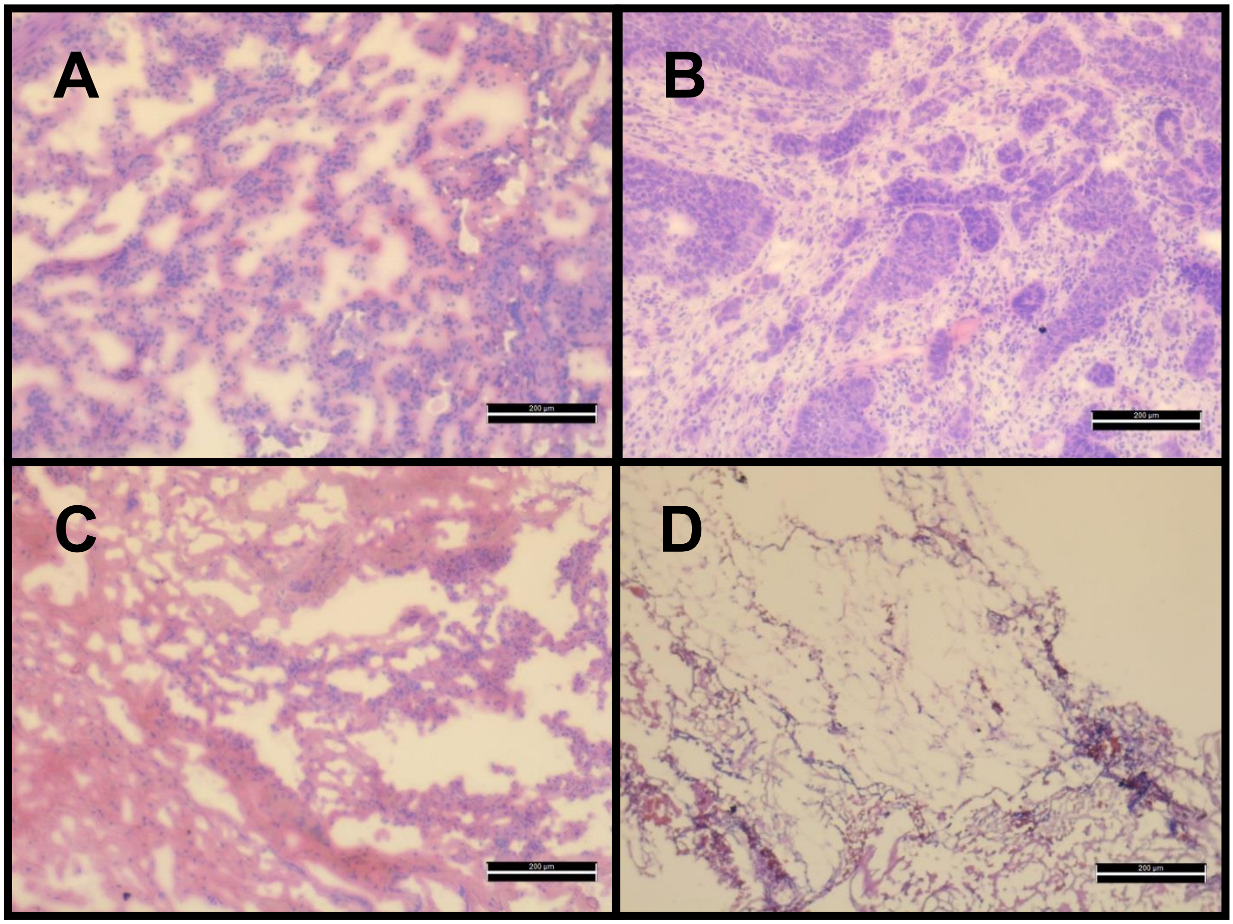


**A:** Normal lung, **B:** Lung adenocarcinoma, **C:** Reactive stroma (Tumor microenvironment, **D:** Lymph node

**Bar:** 200 μM

**Patient ID: 120**

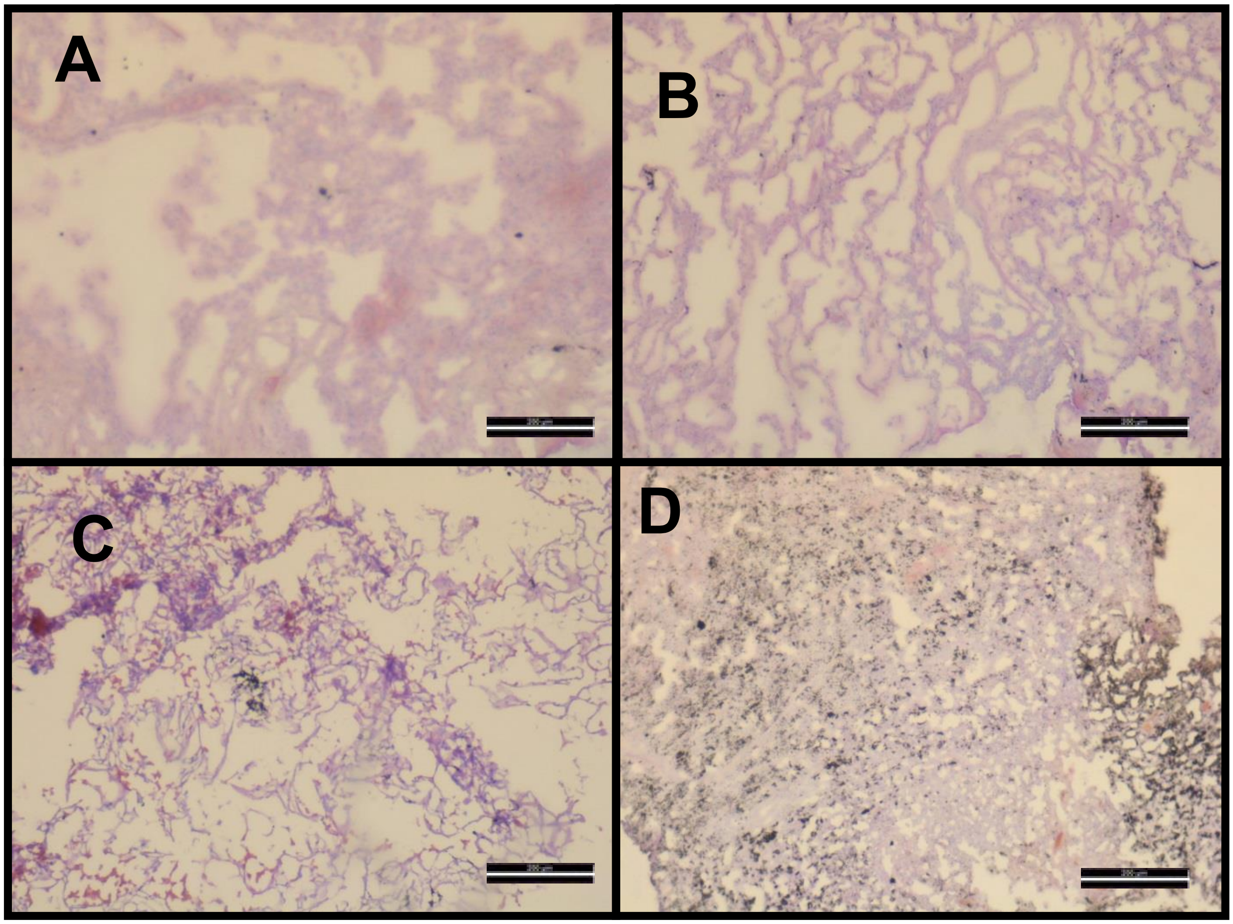


**A:** Normal lung, **B:** Lung adenocarcinoma, **C:** Reactive stroma (Tumor microenvironment, **D:** Lymph node

**Bar:** 200 μM

**Patient ID: 121**

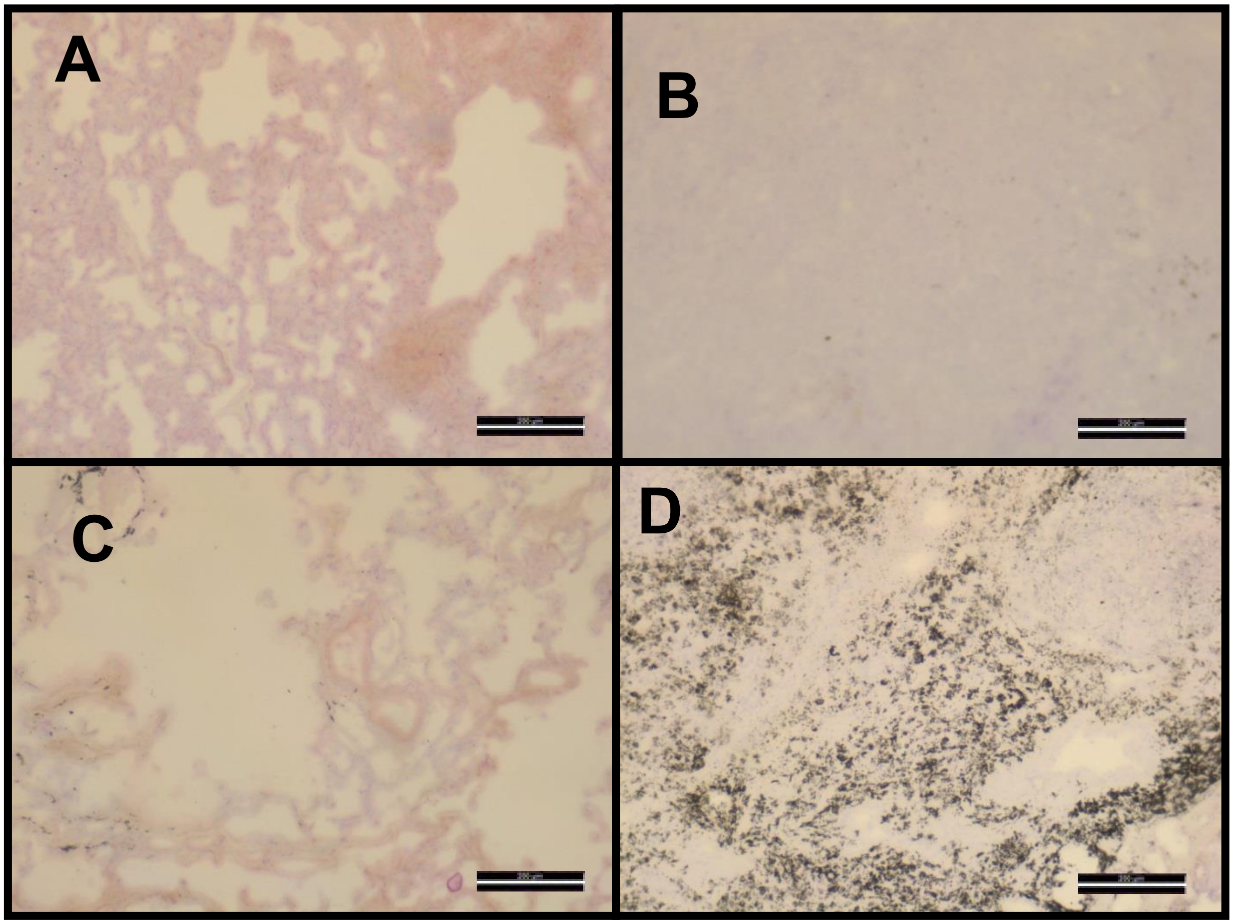


**A:** Normal lung, **B:** Large cell lung carcinoma, **C:** Reactive stroma (Tumor microenvironment, **D:** Lymph node

**Bar:** 200 μM

**Patient ID: 123**

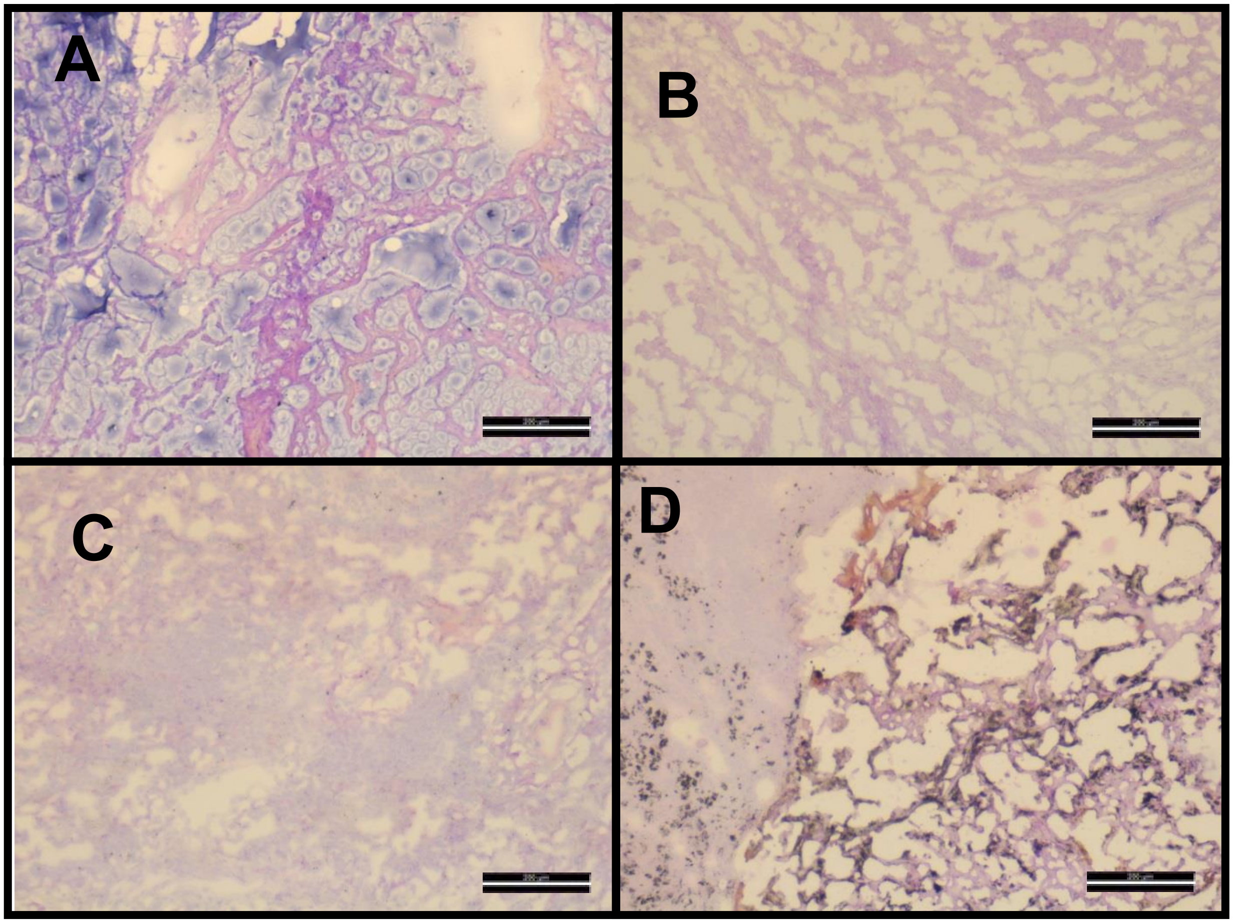


**A:** Normal lung, **B:** Lung adenocarcinoma, **C:** Reactive stroma (Tumor microenvironment, **D:** Lymph node

**Bar:** 200 μM

**Patient ID: 125**

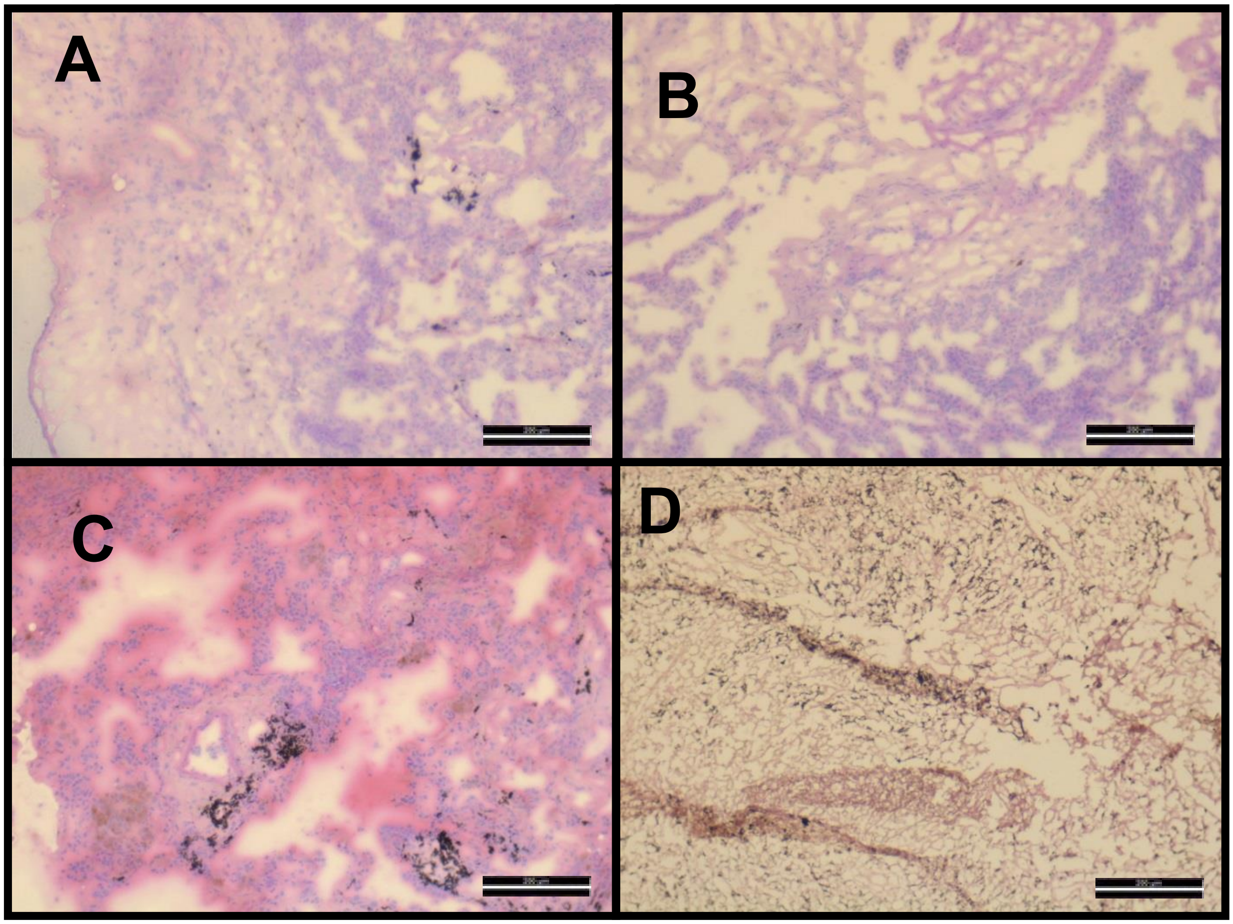


**A:** Normal lung, **B:** Lung adenocarcinoma, **C:** Reactive stroma (Tumor microenvironment, **D:** Lymph node

**Bar:** 200 μM

**Patient ID: 129**

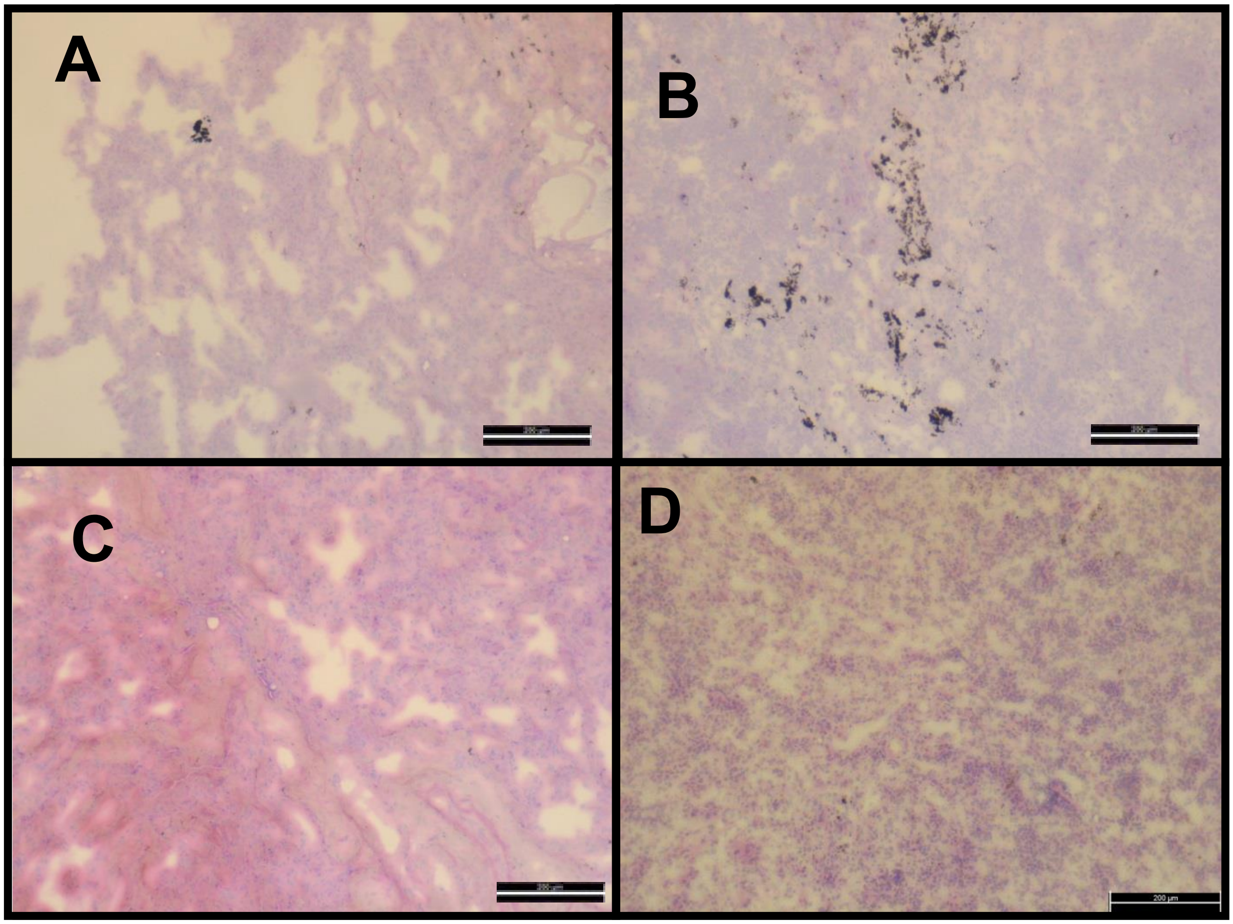


**A:** Normal lung, **B:** Lung adenocarcinoma, **C:** Reactive stroma (Tumor microenvironment, **D:** Lymph node

**Bar:** 200 μM

**Patient ID: 133**

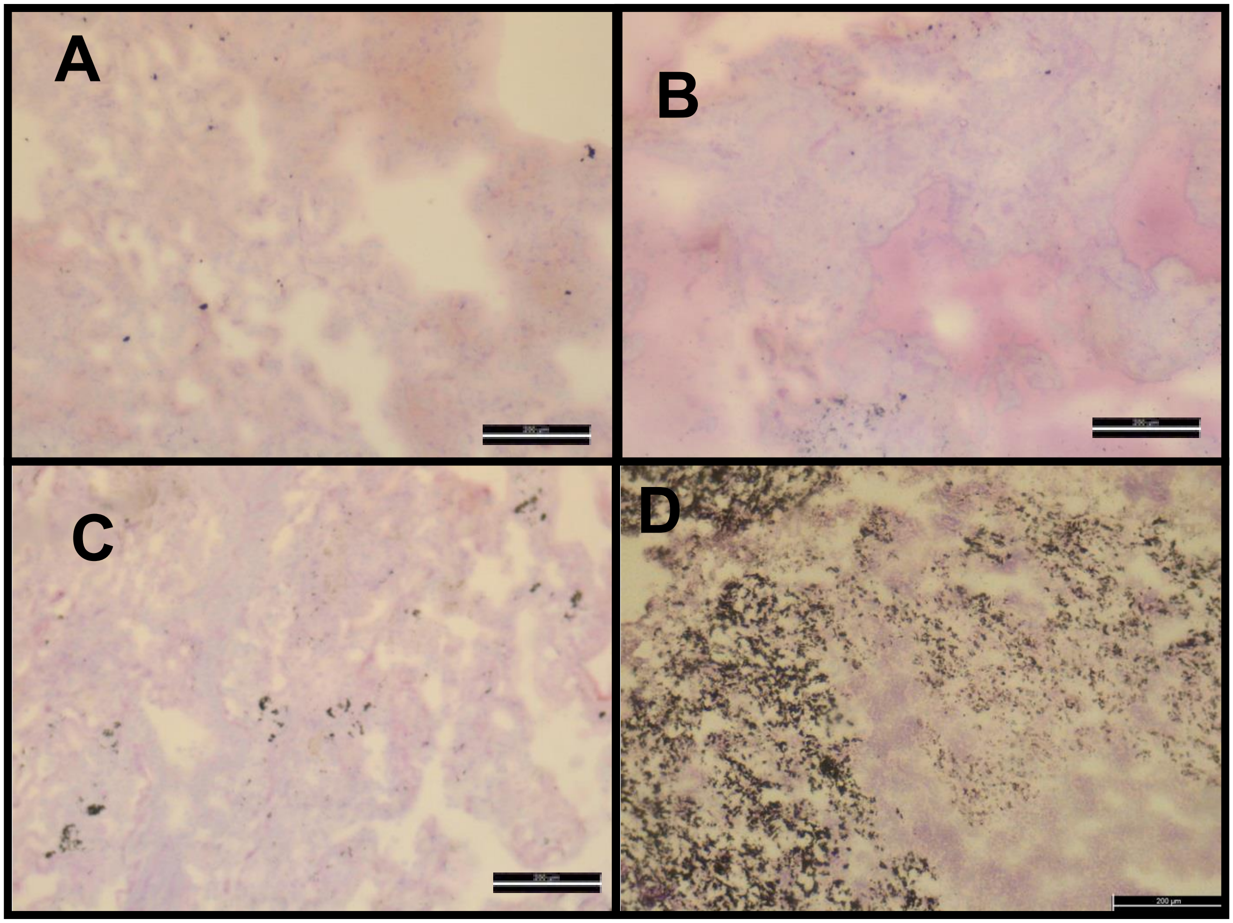


**A:** Normal lung, **B:** Lung adenocarcinoma, **C:** Reactive stroma (Tumor microenvironment, **D:** Lymph node

**Bar:** 200 µM

**Patient ID: 135**

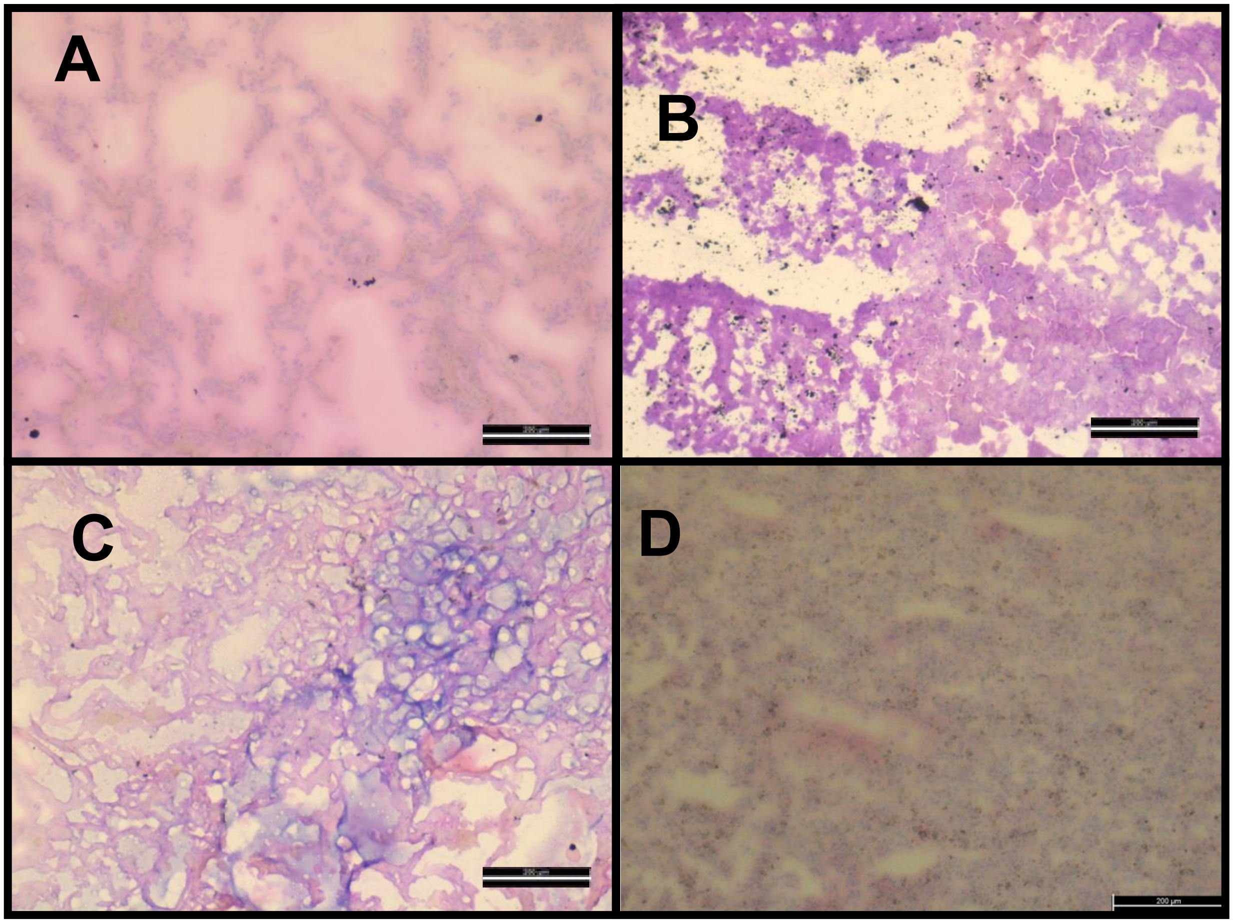


**A:** Normal lung, **B:** Large cell lung carcinoma, **C:** Reactive stroma (Tumor microenvironment, **D:** Lymph node

**Bar:** 200 µM

**Patient ID: 139**

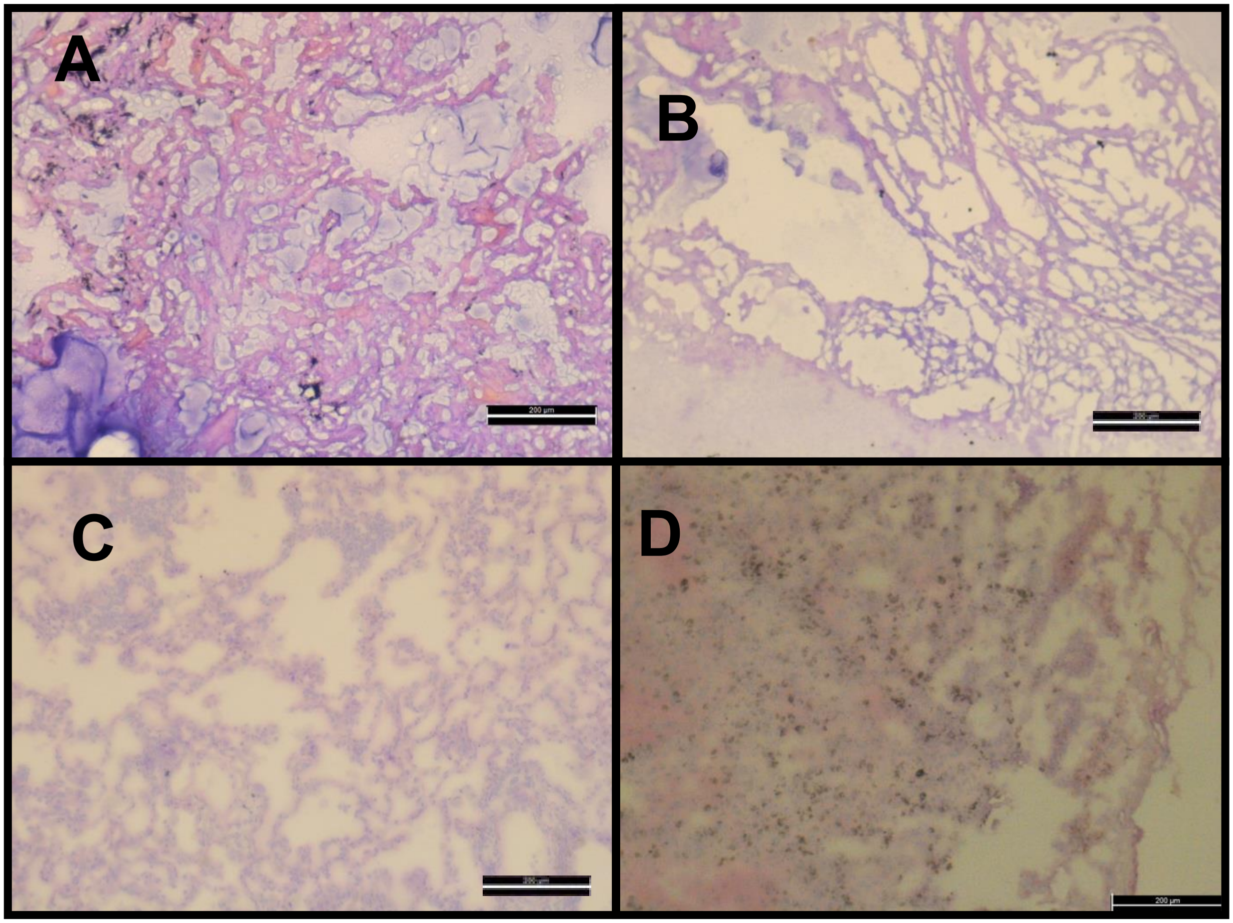


**A:** Normal lung, **B:** Lung adenocarcinoma, **C:** Reactive stroma (Tumor microenvironment, **D:** Lymph node

**Bar:** 200 µM

**Patient ID: 147**

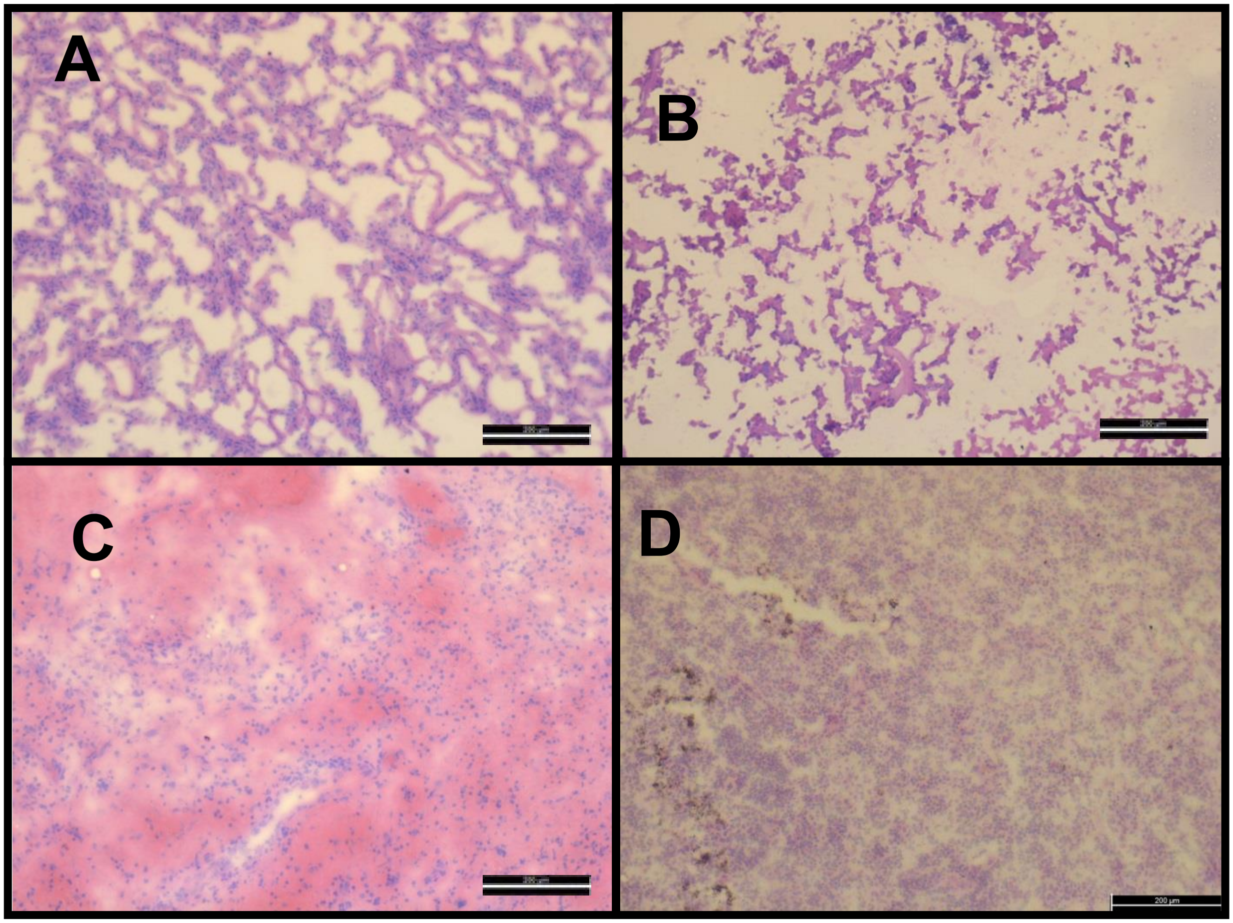


**A:** Normal lung, **B:** Large cell lung carcinoma, **C:** Reactive stroma (Tumor microenvironment, **D:** Lymph node

**Bar:** 200 µM

**Patient ID: 168**

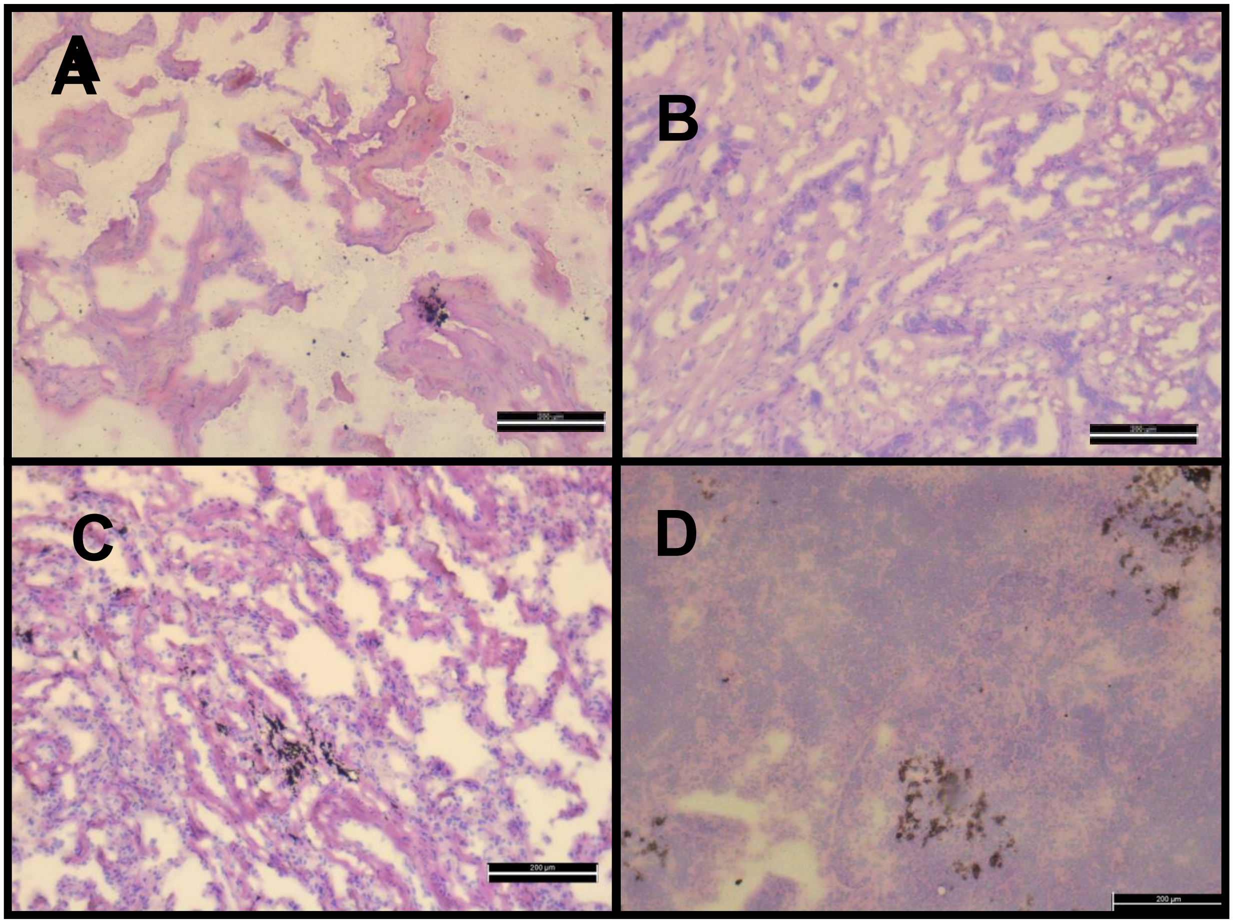


**A:** Normal lung, **B:** Large cell lung carcinoma, **C:** Reactive stroma (Tumor microenvironment, **D:** Lymph node

**Bar:** 200 µM

**Patient ID: 179**

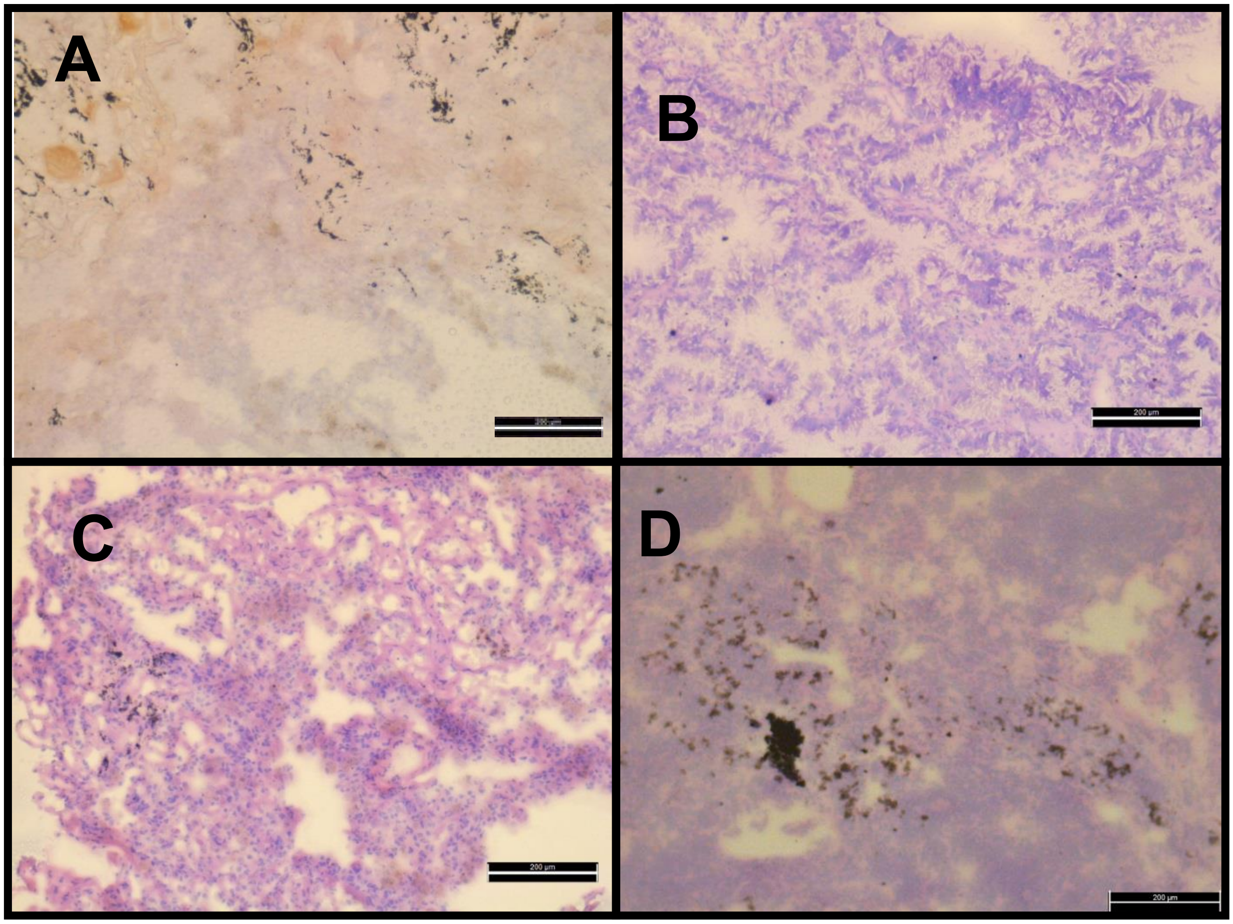


**A:** Normal lung, **B:** Lung adenocarcinoma, **C:** Reactive stroma (Tumor microenvironment, **D:** Lymph node

**Bar:** 200 μM
