# Supplementary material for: LUNGBANK: a novel biorepository strategy tailored for comprehensive multiomics analysis and P-medicine applications in lung cancer
Source: Turk J Biol. 2024 May 28;48(3):203–17. doi: 10.55730/1300-0152.2696 (PMC11265891; doi:10.55730/1300-0152.2696)
Supplement: Supplementary file 4 [file Supplementary_Data-4-QC.docx]

**Quality Control Measures for RNA**

**Table 1a.** Descriptive statistics – Concentration by Qubit

| **Concentration by Qubit (ng/µL)** | **N** | **T** | **R** | **L** | **B** |
| --- | --- | --- | --- | --- | --- |
| **Mean** | 504.14 | 1130.42 | 557.50 | 791.80 | 120.37 |
| **Standard Error** | 34.29 | 99.27 | 41.13 | 65.27 | 11.26 |
| **Median** | 421.00 | 955.00 | 495.50 | 668.00 | 89.60 |
| **Mode** | 442.00 | 510.00 | 534.00 | 232.00 | 121.00 |
|  | 351.00 | 1491.00 | 491.00 | 328.00 | 115.00 |
|  |  | 840.00 | 836.00 | 378.00 |  |
|  |  | 1227.00 |  | 840.00 |  |
|  |  |  |  | 600.00 |  |
| **Standard Deviation** | 290.93 | 842.30 | 348.99 | 550.00 | 84.29 |
| **Sample Variance** | 84642.04 | 709477.62 | 121792.34 | 302501.70 | 7105.27 |
| **Kurtosis** | 0.44 | 5.10 | 2.00 | 6.71 | 4.67 |
| **Skewness** | 0.96 | 1.79 | 1.22 | 2.09 | 1.96 |
| **Range** | 1300.00 | 4737.00 | 1766.00 | 3220.00 | 431.40 |
| **Minimum** | 89.00 | 13.00 | 50.00 | 140.00 | 27.60 |
| **Maximum** | 1389.00 | 4750.00 | 1816.00 | 3360.00 | 459.00 |
| **Sum** | 36298.00 | 81390.50 | 40140.00 | 56218.00 | 6740.50 |
| **Count** | 72.00 | 72.00 | 72.00 | 71.00 | 56.00 |

**Table 1b.** Concentration by Qubit for all samples

| **Concentration-Qubit fluorometer (ng/µL)** | | | | | |
| --- | --- | --- | --- | --- | --- |
| **Patient ID** | **N** | **T** | **R** | **L** | **B** |
| **1** | 190 | 2421 | 385 | 1040 | 85 |
| 2 | 267 | 960 | 231 | 232 | 89,5 |
| 3 | 407 | 4000 | 500 | 1140 | 87 |
| 4 | 442 | 1976 | 944 | 3360 | 245 |
| 5 | 944 | 1620 | 1590 | 1156 | 107 |
| 8 | 163 | 752 | 572 | 636 | 138 |
| 10 | 704 | 510 | 896 | 1272 | 49,2 |
| 11 | 556 | 2010 | 918 | 1160 | NA |
| 12 | 260 | 1520 | 1330 | 522 | NA |
| 13 | 426 | 1110 | 790 | 792 | 280 |
| 14 | 320 | 934 | 368 | 588 | 67,7 |
| 16 | 359 | 1660 | 318 | 736 | 192 |
| 17 | 804 | 1491 | 797 | 1568 | 121 |
| 18 | 432 | 1960 | 788 | 712 | NA |
| 20 | 351 | 282 | 117 | 168 | NA |
| 21 | 332 | 1120 | 185 | 328 | 54,7 |
| 22 | 628 | 1404 | 292 | 261 | 87,4 |
| 23 | 416 | 1500 | 1230 | 1320 | 88,4 |
| 24 | 358 | 222 | 534 | 1200 | 89,7 |
| 25 | 584 | 491 | 856 | 682 | 230 |
| 26 | 1041 | 812 | 866 | 1420 | 79,5 |
| 27 | 520 | 161 | 240 | 352 | 121 |
| 28 | 624 | 1734 | 1816 | 1800 | 180 |
| 30 | 413 | 824 | 305 | NA | NA |
| 31 | 250 | 840 | 50 | 356 | NA |
| 32 | 272 | 535 | 415 | 716 | 175 |
| 33 | 304 | 668 | 297 | 203 | NA |
| 34 | 532 | 1750 | 224 | 229 | 144 |
| 35 | 500 | 1764 | 205 | 440 | 136 |
| 37 | 300 | 382 | 188 | 312 | 96 |
| 38 | 776 | 1227 | 580 | 546 | 459 |
| 39 | 185 | 840 | 556 | 378 | 190 |
| 40 | 278 | 1328 | 399 | 516 | 115 |
| 41 | 208 | 550 | 742 | 1452 | 137 |
| 42 | 578 | 2940 | 634 | 694 | NA |
| 43 | 399 | 428 | 148 | 205 | NA |
| 44 | 117 | 319 | 130 | 376 | 181 |
| 46 | 237 | 804 | 308 | 580 | NA |
| 47 | 276 | 664 | 396 | 482 | 337 |
| 48 | 240 | 520 | 367 | 1173 | NA |
| 49 | 1048 | 1144 | 442 | 1100 | 68 |
| 51 | 204 | 510 | 282 | 498 | 165 |
| 52 | 846 | 13 | 596 | 666 | 105 |
| 54 | 427 | 307 | 802 | 393 | 58,8 |
| 55 | 402 | 2106 | 718 | 894 | 27,6 |
| 56 | 168 | 1491 | 1060 | 978 | 42,9 |
| 57 | 806 | 287 | 482 | 336 | NA |
| 58 | 362 | 1170 | 550 | 668 | NA |
| 61 | 700 | 950 | 491 | 1398 | 40,5 |
| 63 | 89 | 2650 | 128 | 378 | 67,6 |
| 67 | 115 | 2140 | 191 | 328 | NA |
| 68 | 918 | 1584 | 299 | 515 | 78,7 |
| 70 | 236 | 93 | 174 | 232 | 37,4 |
| 71 | 478 | 708 | 770 | 912 | 83 |
| 76 | 382 | 386 | 534 | 855 | NA |
| 78 | 442 | 1616 | 636 | 700 | 57,5 |
| 81 | 283 | 368 | 166 | 140 | NA |
| 90 | 698 | 4750 | 927 | 2538 | 115 |
| 102 | 752 | 1134 | 570 | 554 | 154 |
| 104 | 750 | 900 | 836 | 840 | 48 |
| 105 | 1030 | 414,5 | 400 | 1065 | 65 |
| 120 | 376 | 993 | 218 | 964 | 61,2 |
| 121 | 902 | 1240 | 1203 | 1000 | 345 |
| 123 | 1389 | 244 | 654 | 566 | 77,3 |
| 125 | 1300 | 1695 | 414 | 574 | 143 |
| 129 | 477 | 1600 | 323 | 600 | 125 |
| 133 | 510 | 453 | 477 | 1030 | 84,7 |
| 135 | 351 | 1576 | 458 | 1900 | 33 |
| 139 | 234 | 1400 | 766 | 600 | 72,5 |
| 147 | 936 | 1227 | 584 | 318 | 85,2 |
| 168 | 740 | 866 | 491 | 880 | 67,5 |
| 170 | 192 | 547 | 836 | 840 | 35 |
| 179 | 952 | 216 | 540 | 895 | 120 |

**Table 2a.** Descriptive statistics – Concentration by Nanodrop

| **Concentration-Nanodrop** | **N** | **T** | **R** | **L** | **B** |
| --- | --- | --- | --- | --- | --- |
| **Mean** | 574.12 | 1263.72 | 616.74 | 917.05 | 143.38 |
| **Standard Error** | 34.17 | 87.03 | 37.62 | 71.66 | 13.78 |
| **Median** | 483.61 | 1105.00 | 567.00 | 731.07 | 120.00 |
| **Mode** | 737.00 | #N/A | #N/A | #N/A | 69.00 |
|  |  |  |  |  | 85.00 |
|  |  |  |  |  | 96.00 |
|  |  |  |  |  | 120.00 |
|  |  |  |  |  | 78.00 |
| **Standard Deviation** | 291.98 | 743.62 | 321.40 | 608.09 | 104.06 |
| **Sample Variance** | 85253.15 | 552974.49 | 103298.74 | 369774.80 | 10828.29 |
| **Kurtosis** | 3.02 | 0.32 | 1.08 | 3.40 | 7.89 |
| **Skewness** | 1.45 | 0.76 | 1.11 | 1.65 | 2.55 |
| **Range** | 1500.22 | 3578.08 | 1579.25 | 3158.00 | 545.00 |
| **Minimum** | 159.78 | 21.92 | 84.75 | 37.00 | 34.00 |
| **Maximum** | 1660.00 | 3600.00 | 1664.00 | 3195.00 | 579.00 |
| **Sum** | 41910.61 | 92251.92 | 45022.12 | 66027.39 | 8172.89 |
| **Count** | 73 | 73 | 73 | 72 | 57 |

**Table 2b.** Concentration by Nanodrop for all samples

| **Concentration - Nanodrop spectrophotometer (ng/µL)** | | | | | |
| --- | --- | --- | --- | --- | --- |
| **Patient ID** | **N** | **T** | **R** | **L** | **B** |
| 1 | 266,73 | 2421 | 332,73 | 764,49 | 90 |
| 2 | 297,62 | 947,45 | 245,21 | 225,96 | 40 |
| 3 | 424,54 | 2719,11 | 524,35 | 947,32 | 127 |
| 4 | 423,87 | 1696,7 | 665,78 | 2495,31 | 192 |
| 5 | 734,62 | 1248,58 | 1308,44 | 1314 | 107 |
| 8 | 208,17 | 725,55 | 575,64 | 643,35 | 137 |
| 10 | 707,46 | 590,38 | 886,8 | 1166,07 | 69 |
| 11 | 675,31 | 2190,08 | 948,64 | 1170,98 | NA |
| 12 | 365,26 | 1462,81 | 1183,36 | 539,92 | NA |
| 13 | 421,48 | 1440,75 | 652,75 | 877,56 | 269 |
| 14 | 438,75 | 916,58 | 407,44 | 607,52 | 85 |
| 16 | 306,32 | 1911,32 | 318 | 697,1 | 176 |
| 17 | 853,55 | 1388,4 | 864,22 | 1402,18 | 128 |
| 18 | 483,61 | 2059,59 | 805,92 | 722,39 | NA |
| 20 | 473,79 | 624,9 | 471,34 | 396,35 | NA |
| 21 | 459,48 | 1030,5 | 229,13 | 425,48 | 66 |
| 22 | 611,75 | 1224,88 | 324,62 | 281,54 | 94,48 |
| 23 | 605 | 1750 | 1450 | 1350 | 96 |
| 24 | 400 | 350 | 731 | 1550 | 96 |
| 25 | 555,94 | 504 | 737 | 616,52 | 194 |
| 26 | 981,6 | 814,51 | 885,81 | 1457,75 | 120 |
| 27 | 554,04 | 179,43 | 263,5 | 433,6 | 239 |
| 28 | 658 | 1926,93 | 1226,98 | 1513 | 206 |
| 30 | 479,67 | 846,47 | 368,97 | NA | NA |
| 31 | 306,68 | 927,44 | 84,75 | 466,22 | NA |
| 32 | 361,94 | 2082,4 | 570,67 | 985,83 | 183 |
| 33 | 369,8 | 715,78 | 370,25 | 234,66 | NA |
| 34 | 697 | 1988,51 | 256,75 | 277,5 | 156 |
| 35 | 540,58 | 1861,77 | 361,91 | 490,98 | 172 |
| 37 | 234,17 | 492,93 | 352,75 | 454,47 | 110 |
| 38 | 752,06 | 1288,9 | 682,47 | 644,64 | 579 |
| 39 | 258,14 | 1056,87 | 653,88 | 473,46 | 223 |
| 40 | 359,97 | 1320,23 | 368,9 | 574,26 | 136 |
| 41 | 245,91 | 564,91 | 727,84 | 1244,55 | 135 |
| 42 | 634,66 | 2556,25 | 655,93 | 735,13 | NA |
| 43 | 409,26 | 479,65 | 174,19 | 231,11 | NA |
| 44 | 159,78 | 337,53 | 299,55 | 379,84 | 196 |
| 46 | 271,48 | 714,64 | 328,55 | 564,46 | NA |
| 47 | 293,9 | 678,72 | 423,8 | 486,8 | 393 |
| 48 | 306,24 | 596,12 | 441,29 | 1321,56 | NA |
| 49 | 926,7 | 1065,55 | 459,63 | 998,21 | 70 |
| 51 | 416,15 | 590,15 | 360,99 | 1080,38 | 170 |
| 52 | 971,97 | 21,92 | 602,1 | 760,78 | 120 |
| 54 | 461,28 | 483,82 | 798,63 | 475,8 | 62 |
| 55 | 455,47 | 2973,78 | 642,97 | 898,68 | 34 |
| 56 | 194,45 | 1327 | 1051,55 | 892,8 | 60,26 |
| 57 | 840,06 | 352,46 | 585,55 | 459,09 | NA |
| 58 | 788,3 | 1232 | 594 | 609 | NA |
| 61 | 732,9 | 971,67 | 547,99 | 1508,79 | 41 |
| 63 | 285 | 2314 | 368 | 1145 | 72 |
| 67 | 355 | 1868 | 251 | 727 | NA |
| 68 | 1085 | 2156 | 328 | 1788 | 83 |
| 70 | 526 | 294 | 477 | 588 | 78 |
| 71 | 604 | 669 | 711 | 2974 | 91 |
| 76 | 897 | 860 | 1103 | 1913 | NA |
| 78 | 442 | 1364 | 586 | 672 | 63 |
| 81 | 429 | 796 | 458 | 445 | NA |
| 90 | 737 | 3600 | 923 | 3195 | 125 |
| 102 | 804 | 1080 | 588 | 509 | 155 |
| 104 | 737 | 901 | 765 | 943 | 69 |
| 105 | 555 | 1982 | 479 | 1144 | 78 |
| 120 | 433 | 1317 | 307 | 994 | 68 |
| 121 | 682,2 | 1610 | 1142 | 1064 | 536 |
| 123 | 1161 | 265 | 616 | 616 | 85 |
| 125 | 1660 | 1940 | 480 | 627 | 86 |
| 129 | 534 | 1757 | 475 | 907 | 154 |
| 133 | 579 | 503 | 507 | 1257 | 139 |
| 135 | 472 | 1541 | 567 | 1993 | 175 |
| 139 | 349 | 1660 | 1664 | 818 | 280 |
| 147 | 1054 | 1105 | 999 | 292 | 93 |
| 168 | 838 | 1017 | 519 | 575 | 78,35 |
| 170 | 730 | 2835 | 1350 | 37 | 163,5 |
| 179 | 1587 | 1167 | 554,6 | 1957 | 129,3 |

**Table 3a.** Desriptive statistics-RNA purity – 280/260 ratio

| **280/260 ratio** | **N** | **T** | **R** | **L** | **B** |
| --- | --- | --- | --- | --- | --- |
| **Mean** | 2.0845 | 2.0859 | 2.0803 | 2.0840 | 2.0065 |
| **Standard Error** | 0.0070 | 0.0089 | 0.0087 | 0.0079 | 0.0135 |
| **Median** | 2.1000 | 2.1100 | 2.1000 | 2.1000 | 2.0200 |
| **Mode** | 2.12 | 2.12 | 2.09 | 2.10 | 2.01 |
|  |  |  |  | 2.09 |  |
| **Standard Deviation** | 0.0597 | 0.0756 | 0.0746 | 0.0672 | 0.1016 |
| **Sample Variance** | 0.0036 | 0.0057 | 0.0056 | 0.0045 | 0.0103 |
| **Kurtosis** | 8.3615 | 5.1898 | 9.9411 | 9.6423 | 5.9525 |
| **Skewness** | -2.1591 | -2.1861 | -2.7623 | -2.6533 | 0.0530 |
| **Range** | 0.4100 | 0.3700 | 0.4700 | 0.4100 | 0.7400 |
| **Minimum** | 1.7900 | 1.8000 | 1.7000 | 1.7500 | 1.6800 |
| **Maximum** | 2.2000 | 2.1700 | 2.1700 | 2.1600 | 2.4200 |
| **Sum** | 152.1700 | 152.2700 | 151.8600 | 150.0500 | 114.3680 |
| **Count** | 73 | 73 | 73 | 72 | 57 |

**Table 3b.** RNA purity – 280/260 ratio for all samples

| **Nanodrop spectrophotometer - 280/260 ratio** | | | | | |
| --- | --- | --- | --- | --- | --- |
| **Patient ID** | **N** | **T** | **R** | **L** | **B** |
| 1 | 2,07 | 2,04 | 2,09 | 2,14 | 2,01 |
| 2 | 2,08 | 2,17 | 2,09 | 2,02 | 2,08 |
| 3 | 2,12 | 2,1 | 2,15 | 2,14 | 2,09 |
| 4 | 2,12 | 2,13 | 2,13 | 2,11 | 2,04 |
| 5 | 2,14 | 2,14 | 2,14 | 2,13 | 2,01 |
| 8 | 2,07 | 2,07 | 2,05 | 2,1 | 2,03 |
| 10 | 2,15 | 2,13 | 2,14 | 2,15 | 2,08 |
| 11 | 2,16 | 2,14 | 2,17 | 2,16 | NA |
| 12 | 2,12 | 2,13 | 2,14 | 1,75 | NA |
| 13 | 2,11 | 2,14 | 2,14 | 2,15 | 2,06 |
| 14 | 2,11 | 2,12 | 2,11 | 2,12 | 2,01 |
| 16 | 2,12 | 2,15 | 2,13 | 2,15 | 2,15 |
| 17 | 2,09 | 2,09 | 2,08 | 2,07 | 2,06 |
| 18 | 2,05 | 2,08 | 2,07 | 2,09 | NA |
| 20 | 2,11 | 2,17 | 2,12 | 2,05 | NA |
| 21 | 2,11 | 2,15 | 2,09 | 2,11 | 2,04 |
| 22 | 2,1 | 2,13 | 2,11 | 2,12 | 2,01 |
| 23 | 2,01 | 2,1 | 2,15 | 2,02 | 1,9 |
| 24 | 1,9 | 1,8 | 2,13 | 2,02 | 2,01 |
| 25 | 2,11 | 2,14 | 2,11 | 2,12 | 2,04 |
| 26 | 2,12 | 2,13 | 2,14 | 2,14 | 1,81 |
| 27 | 2,1 | 2,1 | 2,14 | 2,1 | 2,05 |
| 28 | 2,12 | 2,12 | 2,12 | 2,14 | 2,03 |
| 30 | 2,12 | 2,12 | 2,09 | NA | NA |
| 31 | 2,08 | 2,14 | 1,96 | 2,09 | NA |
| 32 | 2,11 | 2,12 | 2,13 | 2,14 | 2 |
| 33 | 2,1 | 2,12 | 2,1 | 2,09 | NA |
| 34 | 2,12 | 2,13 | 2,11 | 2,02 | 2 |
| 35 | 2,1 | 2,14 | 2,09 | 2,1 | 2,04 |
| 37 | 2,1 | 2,12 | 2,12 | 2,11 | 2,01 |
| 38 | 2,13 | 2,12 | 2,11 | 2,13 | 2,09 |
| 39 | 2,09 | 2,14 | 2,12 | 2,11 | 2,03 |
| 40 | 2,09 | 2,12 | 2,13 | 2,08 | 1,99 |
| 41 | 2,1 | 2,11 | 2,12 | 2,12 | 2,02 |
| 42 | 2,12 | 2,11 | 2,12 | 2,12 | NA |
| 43 | 2,1 | 2,11 | 2,06 | 2,08 | NA |
| 44 | 2,03 | 2,11 | 2,08 | 2,08 | 2,03 |
| 46 | 2,07 | 2,13 | 2,1 | 2,1 | NA |
| 47 | 2,11 | 2,12 | 2,1 | 2,13 | 2,04 |
| 48 | 2,09 | 2,13 | 2,1 | 2,14 | NA |
| 49 | 2,1 | 2,11 | 2,09 | 2,1 | 1,8 |
| 51 | 2,07 | 1,99 | 1,97 | 2,09 | 2,02 |
| 52 | 2,07 | 1,92 | 2,08 | 2,1 | 2,01 |
| 54 | 2,07 | 2,07 | 2,09 | 2,06 | 1,84 |
| 55 | 2,03 | 2,08 | 2,12 | 2,12 | 2,01 |
| 56 | 2,11 | 2,1 | 2,12 | 2,1 | 2,01 |
| 57 | 2,11 | 2,11 | 2,1 | 2,09 | NA |
| 58 | 2,1 | 2,13 | 2,11 | 2,13 | NA |
| 61 | 2,1 | 2,12 | 2,13 | 2,13 | 2,02 |
| 63 | 2,12 | 2,08 | 2,08 | 2,12 | 2,01 |
| 67 | 2,08 | 1,9 | 2 | 2,09 | NA |
| 68 | 2,02 | 2,03 | 2,05 | 2,03 | 1,78 |
| 70 | 2,1 | 2,11 | 2,11 | 2,03 | 1,86 |
| 71 | 2,03 | 2,04 | 1,96 | 2,02 | 1,96 |
| 76 | 2,12 | 2,12 | 2,13 | 2,13 | NA |
| 78 | 2,07 | 2,09 | 2,09 | 2,08 | 1,9 |
| 81 | 2,2 | 2,13 | 2,1 | 2,03 | NA |
| 90 | 2,08 | 1,87 | 2,1 | 1,9 | 2 |
| 102 | 2,08 | 2,08 | 2,07 | 2,02 | 2,01 |
| 104 | 2,08 | 2,16 | 2,08 | 2,09 | 1,99 |
| 105 | 2,14 | 2,14 | 2,09 | 2,13 | 1,89 |
| 120 | 1,79 | 1,8 | 1,7 | 1,85 | 1,68 |
| 121 | 2,02 | 2,01 | 2,11 | 2,1 | 2,11 |
| 123 | 2,14 | 2,06 | 1,85 | 2,14 | 2,01 |
| 125 | 2,2 | 2,01 | 1,96 | 2,02 | 2,02 |
| 129 | 2,06 | 2,1 | 2,03 | 2,09 | 2,03 |
| 133 | 2,02 | 2,01 | 2 | 2,06 | 2,03 |
| 135 | 2 | 2,02 | 2,06 | 2,12 | 2,02 |
| 139 | 2,07 | 2,07 | 2,08 | 2,1 | 2,42 |
| 147 | 1,98 | 2,01 | 2,01 | 2,01 | 2,03 |
| 168 | 2,12 | 2,14 | 2,06 | 2,11 | 2,04 |
| 170 | 1,96 | 2,06 | 2,08 | 2,09 | 2,07 |
| 179 | 2,08 | 2,04 | 1,87 | 2,03 | 2,04 |

**Table 4a.** Desriptive statistics-RNA purity – 260/230 ratio

| **260/230 ratio** | **N** | **T** | **R** | **L** | **B** |
| --- | --- | --- | --- | --- | --- |
| **Mean** | 1.8621 | 1.8621 | 1.9158 | 1.9644 | 1.7493 |
| **Standard Error** | 0.0290 | 0.0290 | 0.0334 | 0.0282 | 0.0365 |
| **Median** | 1.8700 | 1.8700 | 2.0100 | 2.0400 | 1.8000 |
| **Mode** | 1.80 | 1.80 | 2.15 | 2.11 | 1.91 |
|  |  |  | 2.01 |  |  |
| **Standard Deviation** | 0.2474 | 0.2474 | 0.2853 | 0.2395 | 0.2754 |
| **Sample Variance** | 0.0612 | 0.0612 | 0.0814 | 0.0573 | 0.0759 |
| **Kurtosis** | 1.1062 | 1.1062 | 1.3191 | 2.2821 | 4.0894 |
| **Skewness** | -0.9315 | -0.9315 | -1.2535 | -1.4639 | -1.8733 |
| **Range** | 1.2300 | 1.2300 | 1.3500 | 1.1400 | 1.3000 |
| **Minimum** | 1.0300 | 1.0300 | 0.9200 | 1.1100 | 0.8000 |
| **Maximum** | 2.2600 | 2.2600 | 2.2700 | 2.2500 | 2.1000 |
| **Sum** | 135.9300 | 135.9300 | 139.8500 | 141.4400 | 99.7100 |
| **Count** | 73 | 73 | 73 | 72 | 57 |

**Table 4b.** RNA purity – 260/230 ratio for all samples

| **Nanodrop spectrophotometer - 260/230 ratio** | | | | | |
| --- | --- | --- | --- | --- | --- |
| **Patient ID** | **N** | **T** | **R** | **L** | **B** |
| 1 | 1,8 | 2,09 | 1,5 | 1,98 | 1,8 |
| 2 | 1,27 | 2,13 | 1,66 | 1,62 | 0,8 |
| 3 | 1,63 | 2,13 | 1,55 | 1,98 | 1,81 |
| 4 | 1,35 | 1,85 | 1,94 | 2,16 | 1,6 |
| 5 | 1,94 | 1,8 | 2,05 | 2,23 | 1,8 |
| 8 | 1,84 | 1,93 | 1,84 | 1,77 | 1,91 |
| 10 | 2,26 | 2,18 | 2,26 | 2,11 | 1,73 |
| 11 | 1,59 | 2,14 | 1,29 | 2,02 | NA |
| 12 | 1,79 | 1,99 | 2,15 | 1,11 | NA |
| 13 | 1,03 | 2,12 | 1,91 | 1,68 | 1,87 |
| 14 | 1,96 | 2,17 | 1,98 | 2,15 | 1,95 |
| 16 | 1,91 | 2,05 | 2,11 | 1,41 | 2,05 |
| 17 | 1,39 | 1,82 | 2,16 | 2,19 | 1,26 |
| 18 | 1,87 | 1,95 | 1,87 | 2,04 | NA |
| 20 | 1,83 | 2,2 | 2,12 | 1,75 | NA |
| 21 | 2,05 | 1,97 | 1,72 | 1,96 | 1,79 |
| 22 | 1,77 | 2,13 | 2,08 | 1,59 | 1,38 |
| 23 | 1,8 | 1,95 | 1,96 | 1,98 | 1,7 |
| 24 | 1,89 | 1,8 | 1,73 | 1,85 | 1,63 |
| 25 | 2,07 | 1,83 | 2,09 | 1,79 | 1,71 |
| 26 | 2,08 | 1,94 | 2,2 | 2,13 | 1,56 |
| 27 | 2,02 | 2,11 | 2,19 | 2,14 | 1,9 |
| 28 | 1,79 | 2,21 | 2,15 | 2,22 | 1,57 |
| 30 | 1,84 | 2,14 | 2,03 | NA | NA |
| 31 | 1,88 | 2,13 | 1,7 | 1,93 | NA |
| 32 | 1,52 | 2,18 | 1,44 | 2,01 | 1,9 |
| 33 | 1,69 | 2,09 | 1,44 | 1,19 | NA |
| 34 | 1,8 | 2,25 | 1,2 | 1,67 | 1,8 |
| 35 | 1,98 | 2,09 | 0,92 | 2,07 | 1,91 |
| 37 | 1,91 | 2,12 | 2,09 | 2,09 | 1,91 |
| 38 | 2,19 | 2,21 | 2,18 | 2,12 | 1,99 |
| 39 | 1,71 | 2,02 | 2,17 | 1,65 | 1,92 |
| 40 | 1,87 | 2,18 | 2,14 | 2,07 | 1,86 |
| 41 | 1,46 | 2,04 | 2,11 | 2,14 | 1,98 |
| 42 | 1,87 | 2,16 | 2,03 | 2,05 | NA |
| 43 | 2,02 | 2,08 | 1,54 | 1,93 | NA |
| 44 | 1,28 | 2,1 | 1,8 | 1,89 | 1,91 |
| 46 | 1,94 | 2,21 | 1,63 | 2,07 | NA |
| 47 | 2,09 | 2,13 | 1,95 | 1,66 | 1,92 |
| 48 | 1,84 | 2,02 | 1,83 | 2,09 | NA |
| 49 | 2,16 | 2,06 | 1,67 | 2,11 | 2,1 |
| 51 | 1,6 | 1,54 | 1,46 | 2,16 | 1,9 |
| 52 | 1,88 | 1,01 | 2,05 | 2,11 | 1,86 |
| 54 | 1,79 | 2,08 | 2,17 | 1,64 | 1,65 |
| 55 | 1,67 | 2,12 | 2,17 | 2,13 | 1,93 |
| 56 | 1,7 | 1,48 | 2,12 | 2,14 | 0,81 |
| 57 | 2,16 | 1,97 | 2,01 | 2 | NA |
| 58 | 1,9 | 2,24 | 2,1 | 1,92 | NA |
| 61 | 1,52 | 2,03 | 2,13 | 2,21 | 2,01 |
| 63 | 2,11 | 2,14 | 1,79 | 2,06 | 2,01 |
| 67 | 1,62 | 2 | 2,01 | 2,03 | NA |
| 68 | 2,01 | 1,86 | 2,02 | 2,04 | 1,42 |
| 70 | 2,2 | 2,12 | 1,99 | 1,61 | 1,58 |
| 71 | 1,86 | 1,98 | 1,98 | 2,01 | 1,78 |
| 76 | 2,13 | 2,19 | 2,19 | 2,13 | NA |
| 78 | 1,7 | 2,19 | 2,01 | 2,15 | 1,61 |
| 81 | 2 | 2,2 | 1,87 | 1,6 | NA |
| 90 | 1,78 | 1,9 | 2,15 | 2,02 | 1,72 |
| 102 | 2,1 | 2,13 | 1,86 | 1,71 | 1,65 |
| 104 | 1,8 | 1,62 | 2,15 | 2,25 | 1,56 |
| 105 | 2,06 | 2,03 | 1,98 | 2,13 | 1,65 |
| 120 | 1,81 | 1,8 | 1,68 | 1,89 | 1,65 |
| 121 | 1,85 | 1,78 | 2,1 | 2,18 | 1,66 |
| 123 | 2,18 | 1,64 | 1,27 | 2,15 | 1,78 |
| 125 | 1,78 | 1,69 | 1,56 | 1,89 | 2,01 |
| 129 | 2,11 | 2,21 | 1,96 | 2,07 | 1,98 |
| 133 | 2,1 | 2,15 | 2,08 | 2,22 | 2,01 |
| 135 | 2,03 | 1,98 | 2,26 | 2,14 | 1,98 |
| 139 | 2,17 | 2,2 | 2,27 | 2,24 | 0,98 |
| 147 | 2,03 | 2,08 | 2,1 | 1,91 | 1,98 |
| 168 | 2,19 | 2,18 | 2,01 | 2,19 | 1,87 |
| 170 | 1,92 | 2,07 | 2,24 | 1,8 | 1,89 |
| 179 | 2,19 | 2,14 | 1,73 | 2,11 | 1,76 |

**Table 5a.** Desriptive statistics – RNA Integrity Number

| **RIN** | **N** | **T** | **R** | **L** | **B** |
| --- | --- | --- | --- | --- | --- |
| **Mean** | 7.42 | 7.37 | 7.20 | 7.46 | 6.36 |
| **Standard Error** | 0.07 | 0.10 | 0.09 | 0.07 | 0.13 |
| **Median** | 7.40 | 7.40 | 7.30 | 7.55 | 6.30 |
| **Mode** | 7.20 | 7.00 | 7.20 | 7.90 | 7.30 |
|  | 7.90 | 6.60 |  |  |  |
| **Standard Deviation** | 0.62 | 0.83 | 0.73 | 0.63 | 0.96 |
| **Sample Variance** | 0.38 | 0.68 | 0.53 | 0.39 | 0.93 |
| **Kurtosis** | -0.20 | 1.01 | 1.92 | -0.94 | 0.73 |
| **Skewness** | -0.05 | -0.43 | -0.81 | -0.29 | -0.23 |
| **Range** | 2.80 | 4.70 | 4.40 | 2.50 | 5.20 |
| **Minimum** | 6.00 | 4.40 | 4.80 | 6.10 | 3.70 |
| **Maximum** | 8.80 | 9.10 | 9.20 | 8.60 | 8.90 |
| **Sum** | 541.50 | 537.80 | 525.70 | 536.90 | 362.30 |
| **Count** | 73.00 | 73.00 | 73.00 | 72.00 | 57.00 |

**Table 5b.** RNA Integrity Number for all samples

| **RNA Integrity Number (RIN)** | | | | | |
| --- | --- | --- | --- | --- | --- |
| **Patient ID** | **N** | **T** | **R** | **L** | **B** |
| 1 | 8,30 | 7,00 | 7,40 | 7,00 | 6,10 |
| 2 | 6,90 | 7,70 | 7,20 | 6,80 | 3,70 |
| 3 | 7,10 | 8,40 | 6,60 | 8,10 | 7,60 |
| 4 | 7,20 | 7,60 | 7,60 | 7,60 | 6,20 |
| 5 | 7,20 | 7,10 | 7,20 | 6,50 | 5,30 |
| 8 | 7,00 | 7,30 | 7,70 | 6,90 | 6,30 |
| 10 | 8,00 | 6,70 | 7,20 | 7,70 | 7,00 |
| 11 | 7,40 | 7,60 | 7,10 | 7,40 | NA |
| 12 | 7,60 | 8,60 | 7,40 | 8,10 | NA |
| 13 | 7,60 | 8,10 | 6,40 | 6,40 | 6,40 |
| 14 | 6,70 | 6,70 | 7,70 | 8,20 | 7,00 |
| 16 | 7,00 | 6,90 | 6,30 | 6,60 | 6,50 |
| 17 | 7,90 | 8,50 | 7,80 | 7,70 | 5,40 |
| 18 | 6,80 | 8,50 | 7,20 | 7,60 | NA |
| 20 | 7,20 | 8,10 | 7,10 | 7,40 | NA |
| 21 | 7,20 | 7,00 | 7,50 | 6,50 | 4,10 |
| 22 | 7,50 | 7,50 | 7,60 | 7,30 | 5,40 |
| 23 | 8,30 | 7,70 | 9,20 | 8,40 | 6,30 |
| 24 | 7,60 | 7,20 | 6,80 | 7,10 | 5,00 |
| 25 | 6,70 | 7,00 | 7,10 | 7,00 | 5,10 |
| 26 | 7,00 | 6,50 | 7,00 | 7,50 | 5,90 |
| 27 | 7,90 | 8,10 | 7,70 | 6,70 | 6,00 |
| 28 | 7,20 | 6,90 | 8,30 | 7,30 | 4,60 |
| 30 | 6,50 | 4,40 | 5,90 | NA | NA |
| 31 | 7,20 | 7,70 | 6,60 | 6,50 | NA |
| 32 | 7,00 | 7,20 | 7,30 | 7,20 | 8,90 |
| 33 | 6,40 | 5,80 | 5,50 | 6,70 | NA |
| 34 | 7,10 | 7,40 | 7,30 | 6,40 | 5,80 |
| 35 | 6,00 | 7,00 | 6,40 | 6,40 | 5,50 |
| 37 | 7,90 | 7,60 | 6,90 | 6,60 | 6,00 |
| 38 | 7,20 | 8,30 | 7,60 | 8,20 | 5,70 |
| 39 | 7,90 | 8,20 | 8,00 | 7,50 | 6,20 |
| 40 | 7,20 | 6,60 | 6,20 | 6,60 | 6,30 |
| 41 | 7,80 | 6,80 | 7,80 | 7,80 | 6,50 |
| 42 | 8,80 | 9,10 | 8,00 | 8,10 | NA |
| 43 | 6,60 | 7,40 | 7,10 | 7,00 | NA |
| 44 | 8,40 | 8,80 | 8,10 | 7,60 | 6,20 |
| 46 | 6,90 | 7,80 | 6,90 | 7,70 | NA |
| 47 | 8,00 | 8,00 | 6,80 | 7,70 | 5,70 |
| 48 | 7,30 | 8,80 | 8,00 | 7,80 | NA |
| 49 | 7,50 | 8,20 | 8,00 | 7,90 | 7,30 |
| 51 | 7,30 | 6,60 | 7,40 | 7,90 | 6,80 |
| 52 | 7,30 | 6,90 | 7,20 | 7,00 | 6,80 |
| 54 | 8,30 | 6,60 | 7,30 | 8,40 | 5,80 |
| 55 | 8,00 | 8,00 | 8,00 | 8,20 | 6,30 |
| 56 | 8,50 | 6,80 | 7,40 | 7,90 | 7,30 |
| 57 | 7,30 | 7,10 | 7,20 | 7,50 | NA |
| 58 | 6,50 | 7,00 | 7,70 | 7,40 | NA |
| 61 | 8,30 | 7,50 | 7,70 | 8,00 | 7,30 |
| 63 | 7,90 | 7,90 | 8,00 | 7,40 | 6,70 |
| 67 | 8,00 | 6,40 | 6,80 | 7,60 | NA |
| 68 | 7,60 | 7,90 | 7,30 | 7,90 | 4,90 |
| 70 | 8,10 | 7,40 | 7,70 | 8,20 | 6,60 |
| 71 | 7,90 | 7,20 | 7,60 | 8,40 | 6,90 |
| 76 | 6,50 | 8,30 | 7,80 | 8,30 | NA |
| 78 | 7,00 | 6,10 | 7,30 | 7,70 | 6,70 |
| 81 | 7,10 | 8,60 | 7,60 | 8,10 | NA |
| 90 | 7,90 | 7,50 | 7,30 | 8,40 | 6,70 |
| 102 | 7,80 | 7,80 | 6,40 | 7,20 | 6,50 |
| 104 | 7,60 | 6,50 | 7,00 | 7,90 | 7,60 |
| 105 | 7,40 | 8,40 | 6,90 | 7,50 | 5,90 |
| 120 | 6,20 | 6,30 | 4,80 | 6,80 | 5,90 |
| 121 | 6,80 | 6,50 | 7,00 | 7,10 | 6,80 |
| 123 | 7,70 | 7,80 | 8,10 | 7,70 | 5,80 |
| 125 | 7,00 | 6,90 | 6,10 | 7,40 | 7,30 |
| 129 | 7,90 | 6,60 | 7,20 | 6,50 | 7,00 |
| 133 | 8,10 | 7,60 | 7,20 | 7,90 | 7,40 |
| 135 | 7,40 | 6,50 | 5,90 | 7,90 | 7,70 |
| 139 | 7,40 | 7,70 | 7,50 | 8,10 | 6,60 |
| 147 | 6,60 | 6,80 | 6,80 | 6,50 | 7,70 |
| 168 | 7,50 | 6,10 | 5,10 | 6,10 | 7,30 |
| 170 | 6,00 | 7,20 | 7,00 | 7,90 | 5,90 |
| 179 | 8,80 | 8,30 | 7,90 | 8,60 | 8,10 |

**N:** Normal lung tissue, **T:** Primary lung tumor, **R:** Reactive stroma (Tumor microenvironment), **L:** Lymph node**, B:** Blood**, RIN:** RNA Integrity Number, **Q:** Qubit, **ND:** Nanodrop
